# Increased somatic mutation burdens in normal human cells due to defective DNA polymerases

**DOI:** 10.1038/s41588-021-00930-y

**Published:** 2021-09-30

**Authors:** Philip S. Robinson, Tim H. H. Coorens, Claire Palles, Emily Mitchell, Federico Abascal, Sigurgeir Olafsson, Bernard C. H. Lee, Andrew R. J. Lawson, Henry Lee-Six, Luiza Moore, Mathijs A. Sanders, James Hewinson, Lynn Martin, Claudia M. A. Pinna, Sara Galavotti, Raheleh Rahbari, Peter J. Campbell, Iñigo Martincorena, Ian Tomlinson, Michael R. Stratton

**Affiliations:** 1 Wellcome Sanger Institute, Hinxton, UK; 2 Department of Paediatrics, University of Cambridge, Cambridge, UK; 3 Institute of Cancer and Genomic Sciences, University of Birmingham, Birmingham, UK; 4 Hereditary Gastrointestinal Cancer Genetic Diagnosis Laboratory, Department of Pathology, The University of Hong Kong, Queen Mary Hospital, Pokfulam, Hong Kong; 5 Department of Haematology, Erasmus University Medical Centre, Rotterdam, the Netherlands; 6 Edinburgh Cancer Research Centre, IGMM, University of Edinburgh, Edinburgh, UK

**Keywords:** Genomics, Gastrointestinal cancer, Ageing

## Abstract

Mutation accumulation in somatic cells contributes to cancer development and is proposed as a cause of aging. DNA polymerases Pol ε and Pol δ replicate DNA during cell division. However, in some cancers, defective proofreading due to acquired *POLE*/*POLD1* exonuclease domain mutations causes markedly elevated somatic mutation burdens with distinctive mutational signatures. Germline *POLE*/*POLD1* mutations cause familial cancer predisposition. Here, we sequenced normal tissue and tumor DNA from individuals with germline *POLE*/*POLD1* mutations. Increased mutation burdens with characteristic mutational signatures were found in normal adult somatic cell types, during early embryogenesis and in sperm. Thus human physiology can tolerate ubiquitously elevated mutation burdens. Except for increased cancer risk, individuals with germline *POLE*/*POLD1* mutations do not exhibit overt features of premature aging. These results do not support a model in which all features of aging are attributable to widespread cell malfunction directly resulting from somatic mutation burdens accrued during life.

## Abstract (web summary)

Healthy tissues from individuals with germline mutations in *POLE* or *POLD1* show increased mutational burden, suggesting that normal cells are capable of tolerating high mutation rates.

## Main

Replication of the genome is required at each cell division. It is effected by DNA polymerases synthesizing a new DNA strand with a sequence dictated by a template strand. Low error rates are ensured by the fidelity of base incorporation, proofreading capabilities of the polymerases and surveillance by the DNA mismatch repair machinery. DNA replication in humans is largely performed by the polymerases Pol ε and Pol δ, which undertake leading and lagging strand synthesis, respectively^1,2^.

Uniquely among nuclear polymerases, both Pol ε and Pol δ have proofreading activities mediated by their exonuclease domains, which identify and remove mismatched bases^1,3–5^. Somatically acquired heterozygous missense mutations in the *POLE* or *POLD1* exonuclease domains found in some human cancers cause defective proofreading and, consequently, high burdens of somatic mutations with distinctive mutational signatures^6–9^. Cancers with *POLE* exonuclease domain mutations show very high single-base substitution (SBS) mutation burdens whereas those with *POLD1* exonuclease domain mutations show less elevated SBS burdens but are often associated with microsatellite instability^8^. Mutations generated by proofreading-defective *POLE* and *POLD1* show marked replication strand bias consistent with their differential roles in leading and lagging strand synthesis^1,2,8^. Polymerases with these mutations also cause mutator phenotypes when engineered into yeast and mice^10–15^.

*POLE* and *POLD1* exonuclease domain mutations can also be inherited through the germline, causing a rare autosomal dominant familial cancer predisposition syndrome known as polymerase proofreading-associated polyposis (PPAP), characterized primarily by early-onset colorectal and endometrial tumors^16–18^. It is plausible that an increased somatic mutation rate underlies this cancer predisposition, and high somatic mutation loads have been reported in the small number of neoplasms analyzed from such individuals^16^. However, whether the mutation rate is elevated in normal cells, or in neoplastic cells only, is not known. If elevated in normal cells, the magnitude of the increase, whether it is raised over the whole lifespan, the range of tissues and fraction of cells in each tissue it affects, and the impact of subsequent neoplastic change are important questions to address in elucidating the pathogenesis of neoplastic transformation.

Accrual of somatic mutations has been proposed as the primary biological mechanism underlying aging^19–24^. This hypothesis is based on the premises that (1) mutations accumulate throughout life and (2) higher mutation loads cause widespread malfunction of cell biology^19,21,22,24^. Recent reports have confirmed that the somatic mutation burden in normal cells does increase during life in a more-or-less linear manner^25–33^, compatible with a causal role for somatic mutations in aging. However, somatic mutations could, in principle, accumulate without significant biological consequences. Thus, study of individuals with inherited *POLE* or *POLD1* exonuclease domain mutations could provide insight into the wider biological consequences of elevated mutation burdens and the pathogenesis of aging.

## Results

### Clinical information and samples

Fourteen individuals, aged between 17 and 72 years and each carrying one of four different germline exonuclease domain mutations in *POLE* or *POLD1* (*POLE* L424V (*n* = 8), *POLD1* S478N (*n* = 4), *POLD1* L474P (*n* = 1) and *POLD1* D316N (*n* = 1)), were studied. Eleven had a history of five or more colorectal adenomas, with age at first polyp diagnosis ranging from 15 to 58 years. Five were diagnosed with colorectal cancer, all before age 50 years, and all had a known family history of colorectal adenoma, colorectal cancer and/or other cancers. No other consistent phenotypic abnormalities were reported (Supplementary Table 1)^18^.

### Mutagenesis in normal intestinal stem cells

The epithelial cell population of an intestinal crypt is a clone derived from a single ancestral crypt stem cell that existed <10 years before sampling^34–37^. Somatic mutations found in the large majority of cells in a crypt, and thus with a high variant allele fraction (VAF), recapitulate the set of mutations present in that ancestral cell^28^. Thus, to investigate somatic mutation burdens and rates in normal cells from *POLE* and *POLD1* germline mutation carriers, 109 normal intestinal crypts (colorectum, *n* = 85; ileum, *n* = 10; duodenum, *n* = 14) were individually isolated by laser-capture microdissection from biopsy and surgical resection samples of 13 individuals and whole-genome sequenced (WGS) (median 33.5-fold coverage) (Methods, Supplementary Note and Supplementary Table 2).

**Fig. 1:**
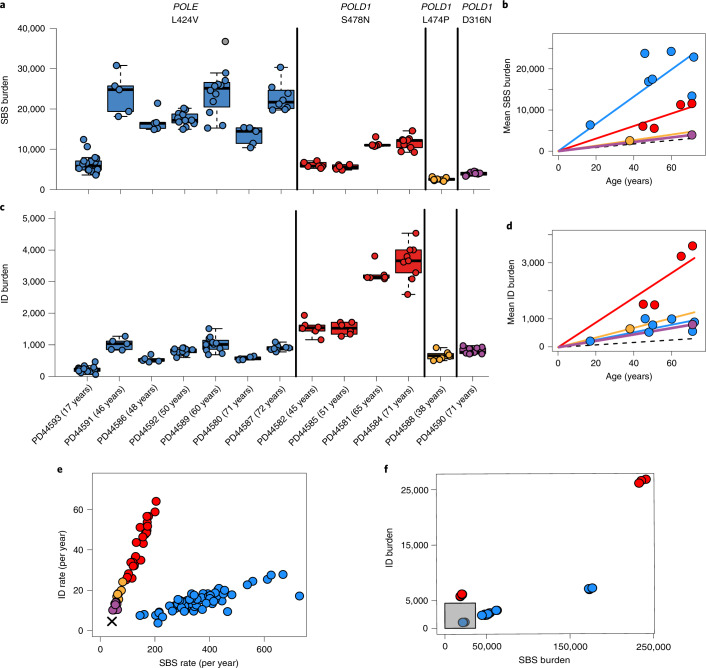
SBS and ID burdens in normal and neoplastic intestinal crypts from individuals with germline *POLE* or *POLD1* mutations. **a**, Genome-wide mutation burden per individual, with the specific germline mutation indicated at the top and color coded (blue, red, green and purple denote *POLE* L424V, *POLD1* S478N, *POLD1* L474P and *POLD1* D316N, respectively). For box-and-whisker plots, the central line, box and whiskers represent the median, interquartile range (IQR) from first to third quartiles, and 1.5 × IQR, respectively. **b**, Mean SBS burden versus age, showing regression lines for the four different germline mutations. The relationship between age and SBS burden in normal individuals is denoted by the dashed line. **c**, Genome-wide ID burden per individual. **d**, Relationship between age and ID burden. **e**, ID rate (per year) versus SBS rate (per year); the cross indicates the SBS and ID rate in normal individuals. **f**, ID and SBS burden in adenomatous samples from individuals with *POLE*/*POLD1* mutations. The gray box indicates the range of mutation burdens in normal intestinal crypts from individuals with *POLE*/*POLD1* mutations.

Somatic SBS burdens in the seven individuals with *POLE* L424V and four with *POLD1* S478N correlated with age, indicating that mutation accumulation is likely to be continuous through life, linear and at similar rates in each individual carrying the same mutation (linear mixed-effects model *R*^2^ = 0.87; Supplementary Note). Crypts from individuals with *POLE* L424V showed an average SBS mutation rate of 331 per year (linear mixed-effects model 95% confidence interval (CI) 259–403, *P* = 10^−12^) (Fig. 1a,b and Supplementary Note). The *POLD1* S478N germline mutations were associated with an SBS rate of 152 per year (linear mixed-effects model 95% CI 128–176*, P* = 10^−17^), and *POLD1* D316N and L474P were associated with an SBS rate of 58 per year (linear mixed-effects model 95% CI 51–65*, P* = 10^−22^). By comparison, intestinal crypts from healthy individuals acquire 49 SBS per year^28^ (linear mixed-effects model 95% CI 46–52, *P* = 10^−36^). Therefore increased somatic SBS rates are present in all normal intestinal cells of individuals with *POLE* or *POLD1* germline mutations (Fig. 1), although there are differences in mutation rates between *POLE* (~sevenfold higher than normal individuals) and *POLD1* (up to threefold higher) germline mutations, and between different *POLD1* mutations. Indeed, individuals with *POLD1* D316N and L474P exhibited relatively modest elevations of SBS rates (~1.2-fold). There was also evidence of differences in SBS rates between the seven individuals with *POLE* L424V, suggesting the existence of genetic and/or environmental modifiers of mutation rate (Fig. 1b and Supplementary Note).

Small insertion and deletion (ID) mutation rates in normal intestinal crypts were also elevated in individuals with germline *POLE*/*POLD1* mutations, with rates of 13 per year (*POLE* L424V), 44 per year (*POLD1* S478N) and 12 per year (*POLD1* D316N and *POLD1* L474P) (linear mixed-effects model, 95% CI 10–16, 35–53, 9–16, *P* = 10^−10^, *P* = 10^−13^ and *P* = 10^−9^, respectively). These are all substantially above the expected rate of one per year in individuals without *POLE* or *POLD1* mutations^28^ (Fig. 1d and Supplementary Note).

Thus, in the normal intestinal crypt, *POLE* germline mutations generally confer greater increases in SBS mutation rates than *POLD1* mutations, whereas *POLD1* mutations confer elevations in ID mutation rates larger than or similar to *POLE* (Fig. 1e). These relative differences in SBS and ID mutation rates are consistent with previous findings in cancer genomes and experimental systems^6,8,11,38–40^. It is noteworthy that the SBS and/or ID mutation burdens in normal intestinal crypts from middle-aged and older individuals with germline polymerase mutations are higher than those observed in many human cancers^41^.

**Extended Data Fig. 1:**
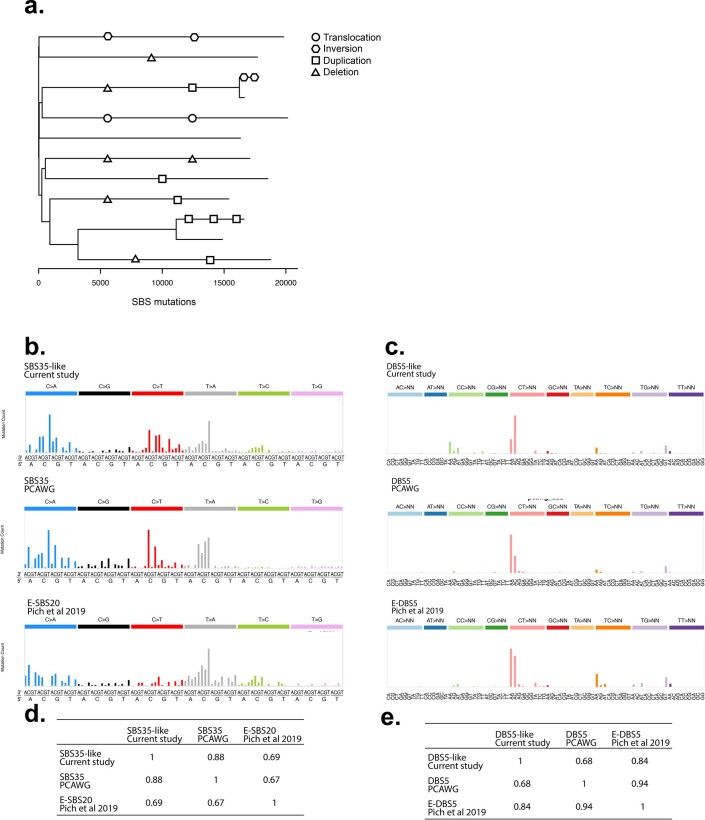
Mutational signatures and structural variants in normal tissue from an individual exposed to oxaliplatin chemotherapy. Mutational signatures identified in normal intestinal crypts from individual PD44592 who had previously undergone treatment with oxaliplatin. Distinctive SBS and DBS signatures were observed that have previously been associated with oxaliplatin treatment in cancer samples. Unexpectedly high numbers of structural variants were also observed in the normal intestinal crypts from this individual and may be due to chemotherapy exposure. **(a)** Phylogenetic tree from individual PD44592 with structural variants (SVs) mapped to branches. Shapes correspond to SV type; translocation (circle), inversion (hexagon), large duplication (square) and deletion (triangle). Ordering of SVs and positioning along branches is arbitrary **(b)** SBS signature SBS35-like displayed above the Pan-Cancer Analysis of Whole Genomes (PCAWG) reference signature SBS35 (PCAWG)^9^ and E-SBS20^48^ **(c)** Doublet Base Substitution (DBS) signature displayed above PCAWG reference signature DBS5 and E-DBS5 from Pich et al 2019^48^. **(d-e)** Cosine similarity matrices for SBS and DBS signatures.

**Extended Data Fig. 2:**
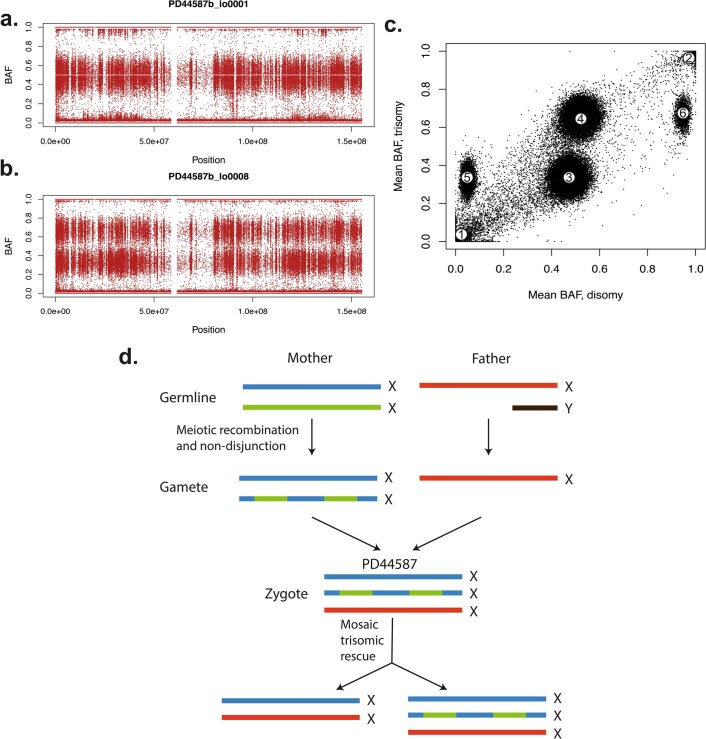
Trisomy X (47XXX) with mosaic trisomic rescue in individual PD44587 identified by lineage tracing of somatic mutations. B-allele frequencies (BAF) of SNP sites for intestinal crypts with two copies of the X-chromosome **(a)** or three **(b)**. Seven crypts exhibited the disomy, whereas one crypt and the majority of blood showed the trisomy. **(c)** The mean BAF of SNPs for samples with a disomic profile versus those with a trisomic profile. SNPs clustered in six distinct groups: those absent from all samples (marked with ‘1’), homozygously present across all samples (2), heterozygous in disomy but in one out of three copies in trisomy (3), heterozygous in disomy but on two copies in trisomy (4), absent from disomy but on one copy in trisomy (5) or homozygous in disomy but on two copies in trisomy (6). The last two clusters are inconsistent with an acquired gain of chromosome X, as they constitute bringing in novel germline SNPs or omission of those previously homozygotic. **(d)** Therefore, this profile can only be explained by a zygote which possessed three copies of X, one of which was mosaically lost in the crypt lineage.

With the exception of one individual who had been treated with oxaliplatin for colorectal cancer and one with an incidental finding of mosaic trisomy of the X chromosome (47 XXX) (Extended Data Figs. 1a and 2), copy number changes and rearrangements were rare, occurring at a prevalence similar to normal crypts from normal individuals (Supplementary Table 2). Reductions in telomere length with age also occurred at rates similar to those of normal crypts from healthy individuals (Supplementary Table 2 and Supplementary Note).

**Extended Data Fig. 3:**
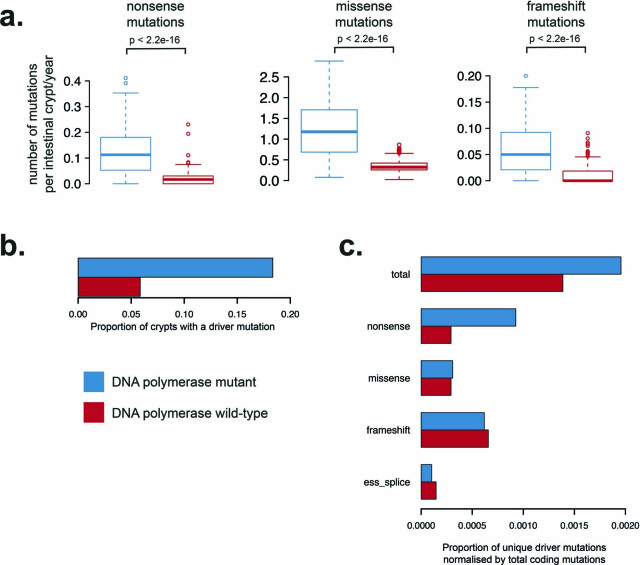
Protein coding mutations due to mutational signatures of defective DNA polymerases. **(a)** Mutation burden per intestinal crypt per year of life. For box and whisker plots, the central line, box and whiskers represent the median, inter-quartile range (IQR) from 1^st^ to 3^rd^ quartiles and 1.5 times the IQR. Elevated burdens of nonsense, missense and frameshift mutations are observed in crypts due to DNA polymerase associated mutational signatures. P-values result from two-sided Wilcoxon rank sum test. **(b)** Comparison of driver mutation burden of normal crypts from individuals with a germline DNA polymerase mutation (blue) and wild-type crypts (red). SBS mutations included are from n = 445 wild-type intestinal crypts^28^ and n = 109 DNA polymerase mutant crypts from the current cohort. **(c)** Comparison of driver mutations normalised by the total number of protein coding mutations. Normalised proportion of driver mutations is displayed on the x-axis. No statistically significant difference in the driver mutation burden between DNA polymerase mutant and DNA polymerase wild-type crypts is observed (Chi-squared test p > 0.05).

Elevated genome-wide mutation burdens are associated with increases in protein-coding mutations (Extended Data Fig. 3a). These include nonsense and frameshift changes that are likely to impair protein function (Extended Data Fig. 3a). There was a greater increase in nonsense mutations (~sevenfold) compared to missense or synonymous mutations (~four- and ~threefold, respectively), which is due to the specific mutational signatures present^42^. There was also an increase in potential cancer ‘driver’ mutation burden compared with normal crypts from normal individuals (20/109 versus 26/445, *P* = 0.00005, chi-squared test; Extended Data Fig. 3b and Supplementary Table 3). However, this was broadly compatible with the increase in overall load and the spectrum of coding mutations in the colon (Extended Data Fig. 3c).

Crypt-like structures from six colorectal adenomas and one carcinoma from individuals with germline *POLE* or *POLD1* mutations were also microdissected and sequenced. SBS and ID burdens were considerably higher than in normal crypts from the same individuals sampled at the same time (Fig. 1e,f), albeit with substantial variation between lesions. Therefore, increases in SBS and ID mutation rates are associated with conversion from a normal crypt to an adenoma or cancer crypt in individuals with *POLE* or *POLD1* germline mutations, a pattern similar to that observed in healthy individuals^43,44^.

### Mutational signatures

**Fig. 2:**
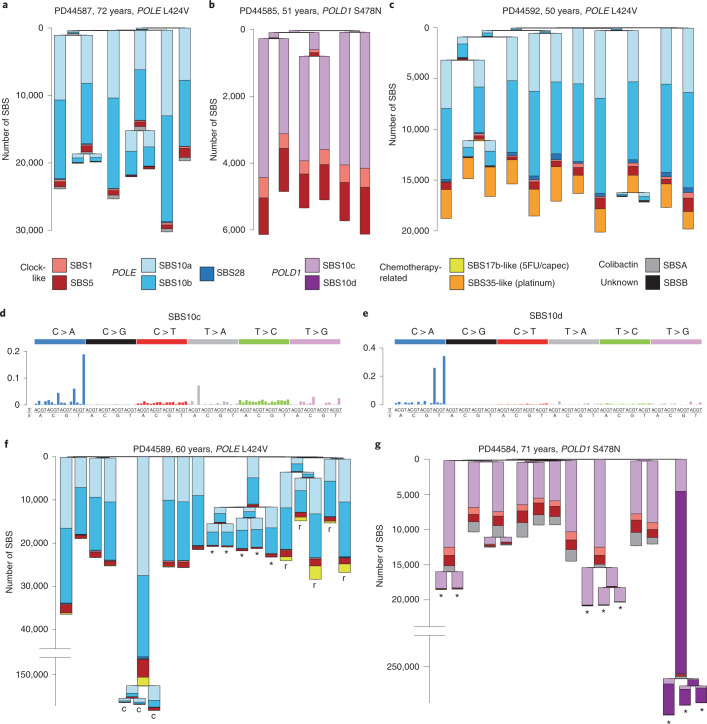
Phylogenies of intestinal crypts with mutational signature annotation. **a**,**b**, Phylogenies of microdissected intestinal crypts of PD44587 (*POLE* L424V) (**a**), exhibiting mainly SBS10a and SBS10b, and PD44585 (*POLD1* S478N) (**b**), exhibiting SBS10c. SBS1 and SBS5, normal signatures of aging, are also present. Signature exposures are color coded as indicated below the trees. Branch lengths correspond to SBS mutation burdens. **c**, In addition to mutagenesis due to *POLE* L424V, PD44592 shows widespread mutagenesis resulting from exposure to a platinum-based chemotherapeutic agent (SBS35-like). **d**,**e**, Probability distributions of SBS10c (**d**) and SBS10d (**e**), two novel signatures associated with *POLD1* mutagenesis. **f**, Phylogeny of PD44589 (*POLE* L424V) containing samples from driver-bearing adenomas (*) and a carcinoma (c). Note that the *y* axis is broken for scale, preserving the original signature proportions. This individual showed mutagenesis due to exposure to capecitabine (capec, SBS17b-like), which was localized to carcinoma samples and nearby normal rectum (marked with r). **g**, Phylogeny of PD44584 (*POLD1* S478N), with driver-bearing adenomas (*). One particular polyp showed extensive hypermutation (note broken *y* axis), largely due to SBS10d. 5FU/capec, capecitabine and fluorouracil. 5FU, 5-fluorouracil; capec, capecitabine.

**Extended Data Fig. 4:**
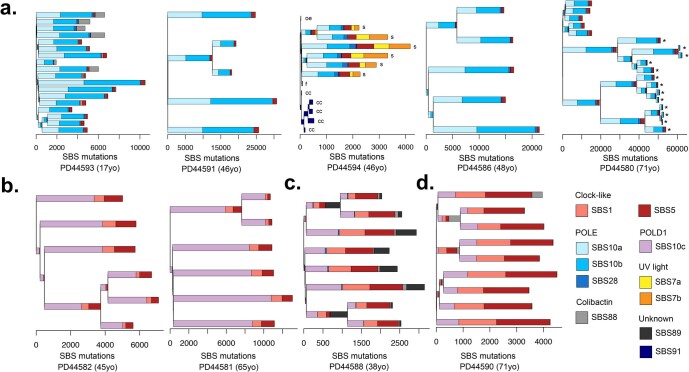
Phylogenetic trees constructed from somatic single base substitution (SBS) mutations annotated with mutational signature exposure. Phylogenetic trees generated from normal and neoplastic crypts displayed per individual. Mutational signature exposure per branch is displayed as stacked bar plots. Ordering of mutational signatures within branches is arbitrary. SBS burden is displayed on the x-axis. Each tip represents a normal intestinal crypt unless otherwise indicated microbiopsies from; ‘s’ skin, ‘cc’ cerebral cortex, ‘oe’ oesophagus, ‘f’ hair follicle and ‘*’ indicates adenomatous crypts. Trees are grouped according to germline DNA polymerase mutation; **(a)** POLE L424V, **(b)** POLD1 S478N, **(c)** POLD1 L474P and **(d)** POLD1 D316N.

**Extended Data Fig. 5:**
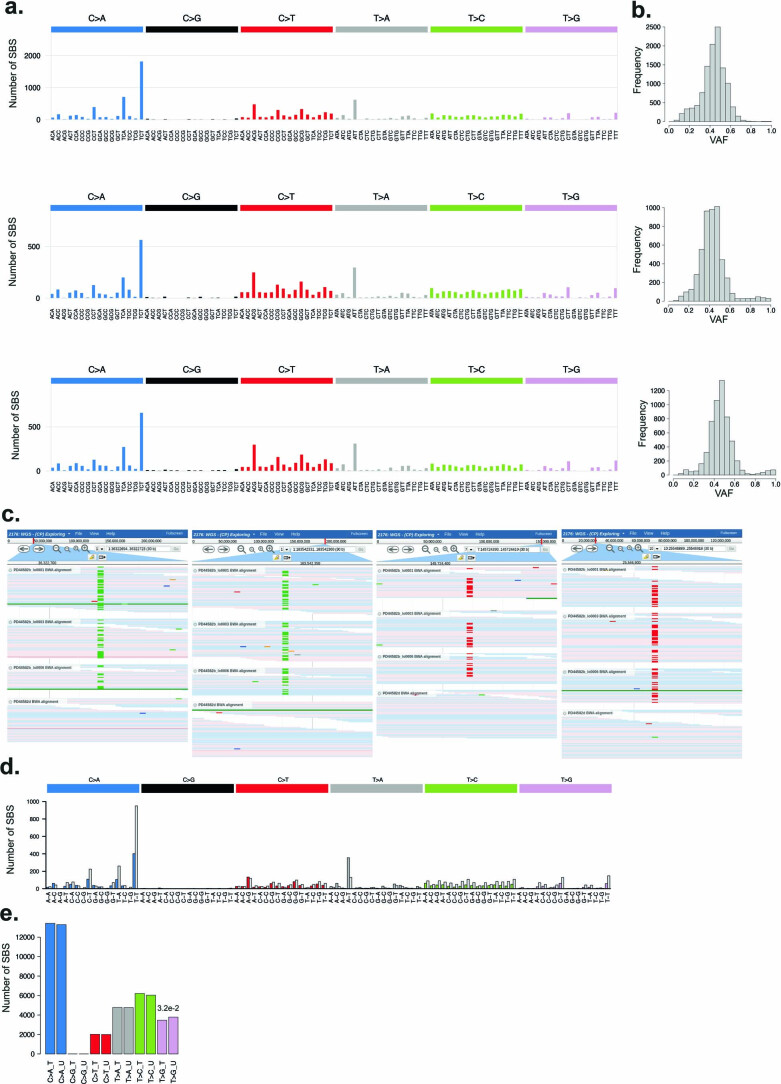
Characterisation of mutational signature SBS10c. **(a)** SBS mutational profiles for representative samples (PD44581b_lo0001, PD44585b_lo0009 and PD44582b_lo0001). **(b)** Histograms of Variant Allele Fraction (VAF) for SBS mutations in each sample. **(c)** JBrowse images showing SBS10c mutations validated across multiple samples. Matched normal samples are displayed at the bottom of each image **(d)** Mutational profile showing replication strand bias of SBS10c mutations from a representative sample. Coloured bars indicate the mutation count on the leading strand and light grey bars indicate the lagging strand. **(e)** Barplot of SBS mutations assigned to SBS10c on the transcribed (T) and un-transcribed (U) strands, p-values were calculated for each mutation type using a two-sided Poisson test, statistically significant p-values are displayed.

**Extended Data Fig. 6:**
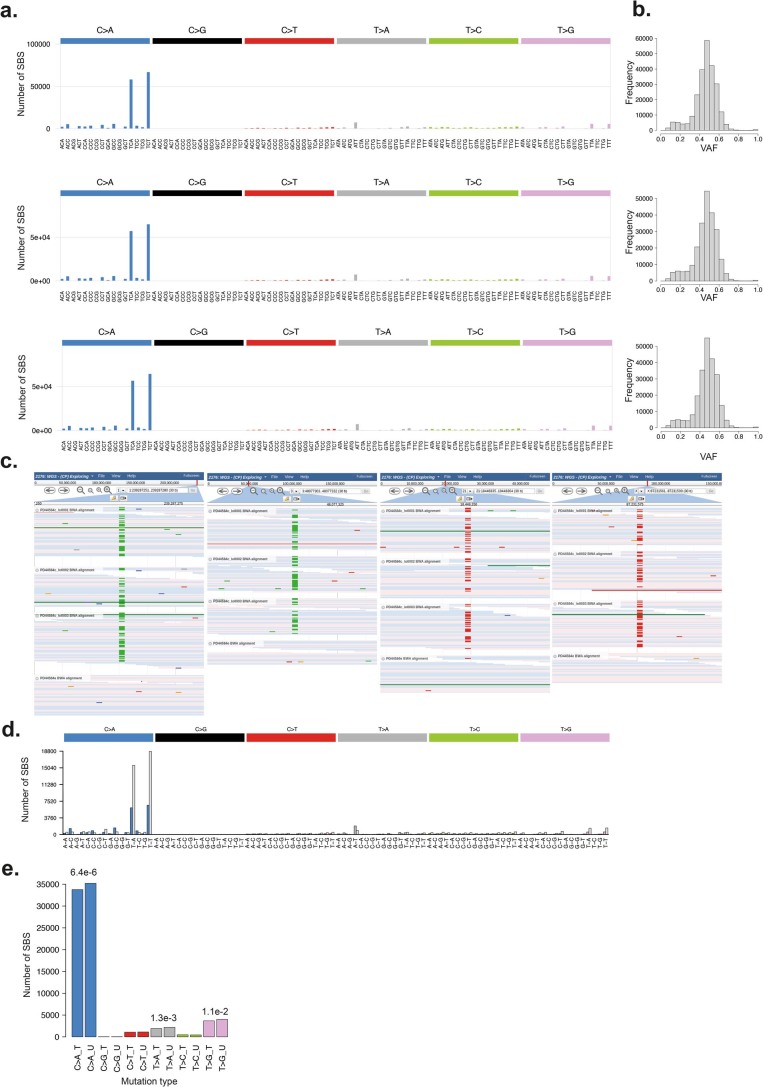
Characterisation of mutational signature SBS10d. **(a)** SBS mutational profiles for samples (PD44584c_lo0001, PD44584c_lo0002 and PD44584c_lo0003). **(b)** Histograms of Variant Allele Fraction (VAF) for SBS mutations in each sample. **(c)** JBrowse images showing clonal SBS10d mutations validated across multiple samples. **(d)** Mutational profile showing replication strand bias of SBS10d mutations from a representative sample. Coloured bars indicate leading strand and light grey bars indicate lagging strand. **(e)** Barplot of SBS mutations assigned to SBS10d on the transcribed (T) and un-transcribed (U) strands, p-values were calculated for each mutation type using a two-sided Poisson test, statistically significant p-values are displayed.

**Extended Data Fig. 7:**
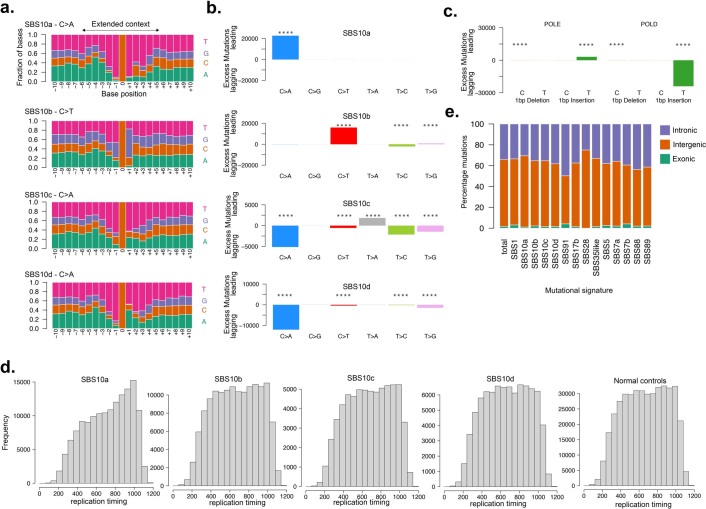
Characterisation of replication strand bias and extended sequence context of mutational signatures associated with germline DNA polymerase mutations. **(a)** Extended sequence context of mutations assigned to signatures SBS10a, SBS10b, SBS10c and SBS10d displayed with pyrimidine annotation. Plots show extended sequence context of C > A and C > T mutations in SBS10a and SBS10b respectively and C > A mutations in SBS10c and SBS10d. **(b)** Replication strand biases of SBS mutations indicated by excess mutations (annotated by pyrimidine base) on either the leading or lagging DNA strand. Biases are displayed according to mutations assigned to known signatures SBS10a and SBS10b and new signatures SBS10c and SBS10d. Only SBS mutations with an assignment probability >0.7 were included (Supplementary Methods). Strong replicative strand asymmetries are seen in known (SBS10a & SBS10b) signatures as well as new signatures; SBS10c and SBS10d. P-values were calculated using two-sided Poisson tests and corrected for multiple testing using the Benjamini-Hochberg method. Mutation types with statistically significant replication strand bias (adjusted p < 0.0001) are annotated with ‘****’. **(c)** ID replication strand asymmetries are displayed for single base insertions and deletions according to the affected polymerase gene; POLE (left) POLD1 (right). Replication strand bias is indicated by excess mutations with single base insertions on the leading strand in POLE mutant cells and on the lagging strand in POLD1 mutant cells. **(d)** Frequency of somatic SBS mutations assigned to each mutational signature SBS10a-d in replication timing bins. **(e)** Distribution of SBS mutations across different genomic regions; exonic, intronic and intergenic.

Eleven SBS mutational signatures were observed in normal intestinal crypts from individuals with *POLE* and *POLD1* germline mutations (Fig. 2a–c and Extended Data Fig. 4). Nine have been previously reported: SBS1, SBS5, SBS10a, SBS10b, SBS17b, SBS28, SBS35, SBS88 and SBS89 (https://cancer.sanger.ac.uk/cosmic/signatures)^9,28^. SBS1 (characterized by C>T substitutions at NCG trinucleotides and probably due to deamination of 5-methylcytosine) and SBS5 (of unknown etiology) are found in all normal intestinal crypts from healthy individuals, where they accumulate in a more-or-less linear manner with age^7,9,28,45^. SBS88 and SBS89 are found in normal intestinal crypts from some healthy individuals and are predominantly acquired during childhood^28,46^. SBS88 is likely due to colibactin, a mutagenic product of a strain of *Escherichia coli* sometimes present in the colon microbiome^47^. SBS10a, SBS10b and SBS28 were previously found in the subsets of colorectal, endometrial and other cancer types with somatically acquired *POLE* mutations^7,9^ (Fig. 2a). Two therapy-associated signatures were identified: an SBS35-like signature associated with platinum-based chemotherapy^9,48^ in an individual treated with oxaliplatin (Fig. 2c and Extended Data Fig. 1b–e), and a signature characterized predominantly by T>G mutations (SBS17b-like) in an individual treated with capecitabine^48,49^(Fig. 2f). Two previously unreported mutational signatures (SBS10c and SBS10d) were observed in normal and neoplastic crypts from individuals with germline *POLD1* mutations. Both were characterized predominantly by C>A substitutions: in SBS10c at ACC, CCA, CCT, TCA and TCT trinucleotides and in SBS10d at TCA and TCT trinucleotides (the mutated bases are underlined) (Fig. 2d,e, Extended Data Figs. 5–7 and Methods).

The increases in SBS burdens in normal intestinal crypts from *POLE* germline mutation carriers compared to healthy individuals were almost completely attributable to SBS10a, SBS10b and SBS28 mutations, and in *POLD1* mutation carriers to SBS10c mutations. By contrast, the estimated burdens of SBS1, SBS5, SBS88 and SBS89 found in normal intestinal crypts from *POLE*/*POLD1* germline mutation carriers were similar to those expected in normal individuals of the same age. Thus, defective *POLE*/*POLD1* proofreading appears not to substantially affect the rates of the mutational processes underlying SBS1, SBS5, SBS88 and SBS89. *POLE* and *POLD1* are responsible for leading and lagging strand DNA synthesis, respectively^1,2^. Consistent with these roles, there was marked replication strand bias of SBS10a and SBS10b somatic mutations in *POLE* germline mutation carriers, with the opposite bias of SBS10c and SBS10d in *POLD1* mutation carriers (Extended Data Fig. 7b).

The elevated mutation loads in adenoma and carcinoma crypts from *POLE*/*POLD1* germline mutation carriers compared to normal intestinal crypts from each individual were also predominantly due to increased burdens of SBS10a and 10b (in *POLE*-mutant cases) and SBS10c and SBS10d (in *POLD1*-mutant cases) (Fig. 2f,g). However, crypts from *POLE*-mutant polyps showed greater relative increases in SBS10a than SBS10b compared to histologically normal *POLE* crypts (Fig. 2f,g). The mechanisms underlying these marked accelerations in mutation rates during neoplastic change, and why they apply differentially to the processes underlying the different signatures, are unknown.

**Extended Data Fig. 8:**
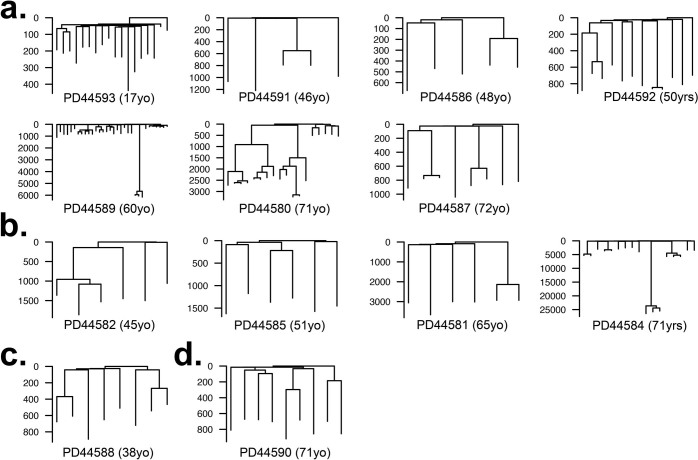
Phylogenetic trees constructed from somatic insertion and deletion (ID) mutations from normal and neoplastic intestinal stem cells. Phylogenetic trees generated from ID mutations. ID mutation burden is displayed on the y-axis. Each tree represents samples from a single individual. Trees are grouped according to germline DNA polymerase mutation **(a)** POLE L424V **(b)** POLD1 S478N **(c)** POLD1 L474P **(d)** POLD1 D316N.

Insertions and deletions in normal intestinal crypts from both *POLE* and *POLD1* germline mutation carriers were dominated by single T insertions at T homopolymer tracts, characteristic of signature ID1. ID1 mutations were also further increased in neoplastic crypts compared to normal crypts from each individual (Fig. 1c,f, Extended Data Fig. 8 and Supplementary Table 2).

**Extended Data Fig. 9:**
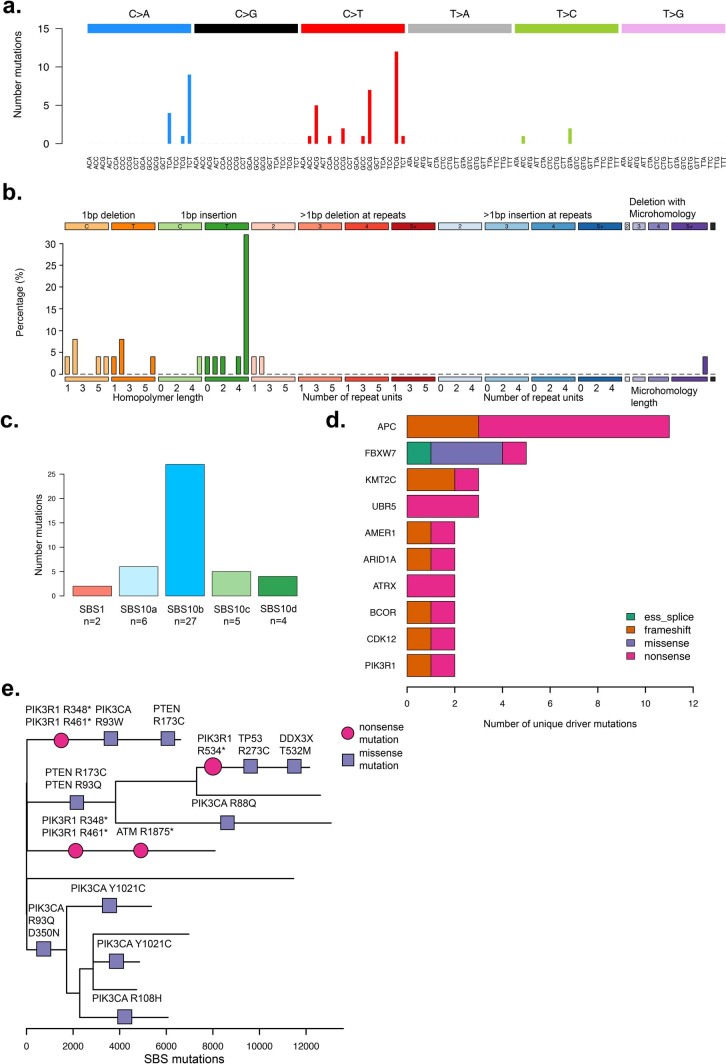
Driver mutation landscape in normal tissues from individuals with POLE and POLD1 germline mutations. Driver mutations in tissues from individuals with germline DNA polymerase mutations. **(a)** Trinucleotide mutational spectrum of SBS driver mutations from normal and neoplastic cells showing characteristic peaks associated with DNA polymerase mutational signatures. **(b)** ID mutation spectrum showing ID type of driver mutations are associated with ID1 mutational signature. **(c)** Frequency of SBS driver mutations from normal and neoplastic cells according to their assigned mutational signature. **(d)** Cancer driver mutations (top 10) identified in histologically normal intestinal crypts displayed in order of frequency, mutation class is indicated by the colour. **(e)** Phylogenetic tree of SBS mutations from endometrial glands from individual PD44589. SBS driver mutations are plotted on the tree, ordering of the drivers within each branch is arbitrary. Driver mutation class is represented by the symbol: nonsense mutations (circles) missense (squares). Gene name and protein change are displayed above each symbol.

Cancer driver mutations found in crypts from normal intestine, and colorectal neoplasms from individuals with *POLE*/*POLD1* germline mutations, showed SBS and ID mutational spectra similar to genome-wide spectra from normal intestinal crypts from these individuals (Extended Data Fig. 9a–c and Supplementary Table 3).

### Mutagenesis in other tissues

**Fig. 3:**
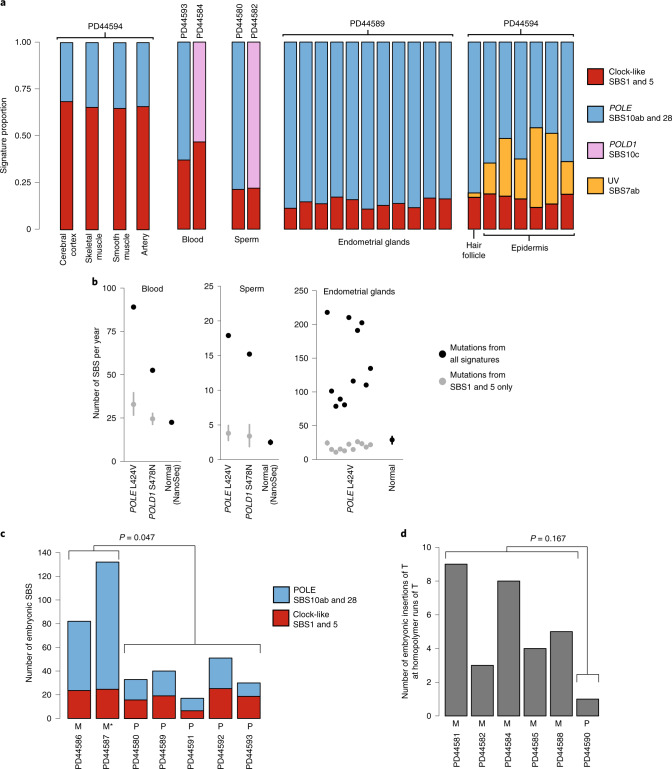
*POLE* and *POLD1* mutagenesis in other tissues. **a**, Signature contribution to mutational landscapes of various tissues in individuals with a *POLE* L424V (PD44594, PD44593, PD44580, PD44589) or *POLD1* S478N (PD44584, PD44582) germline mutation. Normal cerebral cortex, skeletal muscle, smooth muscle, artery, blood and sperm were sequenced using a modified duplex sequencing protocol, while other tissues were subjected to low-input WGS after laser-capture microdissection. Groups of mutational signatures are color coded as indicated. **b**, Estimated genome-wide total mutation rate per year for blood, sperm and endometrium (black dots), as well as yearly mutation burden due to SBS1 and SBS5 (gray dots with 95% CI). Mutation rates of normal controls for blood, sperm and endometrium^31^ are displayed for reference. **c**, Early embryonic SBS in individuals with a *POLE* L424V germline mutation, with a contribution from *POLE* signatures (blue, SBS10a, SBS10b and SBS28) and normal signatures (red, SBS1 and SBS5). M indicates that the mutation was inherited maternally, P paternally; M* indicates presumed maternal inheritance based on pedigree. *P* value is the result of a two-sided Wilcoxon rank-sum test on total counts of mutations attributed to SBS10a, SBS10b and SBS28. **d**, Early embryonic insertions of T at homopolymers of T (indicative of *POLD1* mutagenesis) in individuals with *POLD1* germline mutations (S478N: PD44581, PD44582, PD44584, PD44585; L474P: PD44588; D316N: PD44590). Again, P and M indicate paternal and maternal inheritance, respectively. *P* value is the result of a two-sided Wilcoxon rank-sum test on total counts.

Cancers of the colorectum and endometrium are the predominant types associated with germline and somatic *POLE*/*POLD1* mutations^16–18,50^. Similar to intestinal crypts, endometrial glands are clones derived from a single, recent ancestral stem cell, and whole-genome sequencing of a gland reveals the mutations present in that ancestral cell^31,51^. Eleven endometrial glands dissected from a 60-year-old individual carrying a *POLE* L424V germline mutation showed elevated rates of SBS (148 versus 29 per year in healthy individuals) (Fig. 3b) and ID (6 versus <1 per year in normal individuals) (Supplementary Table 2). SBS10a and SBS10b were responsible for the increase in SBS rate compared to healthy individuals (Fig. 3a,b). Somatic driver mutations in cancer genes are common in normal human endometrium^31,32,52^ and were found in a similar repertoire of genes in all but one gland from this individual (Extended Data Fig. 9e and Supplementary Table 3).

To further investigate mutagenesis in other cell types and embryonic germ layers, we microdissected and sequenced fragments of various tissues from a 46-year-old individual with a germline *POLE* L424V mutation (PD44594). In skin epithelium, aging- and UV-related signatures were accompanied by substantial contributions from SBS10a and SBS10b. The VAFs of mutations generated from the remaining tissues indicated that they contained many cell clones, and thus mutation burdens and mutational signatures were difficult to assess (Supplementary Table 2). For these, we used a modified duplex sequencing protocol technique, termed NanoSeq^53^, to investigate the mutational processes present. By sequencing single DNA molecules at low error rates, this method allows quantification of mutation burdens and signatures from tissues in which cells derived from many progenitors are intimately mixed, and clonal units for sequencing cannot be dissected (Methods). In all tissues we subjected to this method (smooth muscle, skeletal muscle, arterial tunica and cerebral cortex), aging-related signatures were accompanied by SBS10a and SBS10b. Therefore, in all evaluable tissues from this individual, SBS10a and SBS10b were clearly observed, indicating that these tissues carry elevated mutation burdens (Fig. 3a).

Next, we assessed the mutational processes in blood and in sperm from four individuals—two with *POLE* L424V and two with *POLD1* mutations. All samples showed elevated total SBS rates compared with normal controls (Fig. 3a,b). Estimated yearly mutation rates due to SBS1 and SBS5 in blood and sperm were, however, consistent with normal controls^53^ and previous estimates^54,55^, and the excess mutation burdens were, for the most part, due to SBS10a and SBS10b (*POLE-*mutant individuals) and SBS10c (*POLD1-*mutant individuals) (Fig. 3a,b).

Age-related clonal hematopoiesis (ARCH) is a common condition caused by a characteristic set of somatically acquired driver mutations^56,57^. To investigate the effect on ARCH of the elevated mutation rates caused by germline *POLE* and *POLD1* mutations, 22 blood samples from 14 individuals were sequenced to ~10,000-fold coverage for ARCH-associated driver mutations^58^. No evidence of these was observed at the standard 2% VAF threshold. The results are consistent with previous observations that hematological malignancies are not part of the clinical spectrum observed in these individuals^16–18^ and support the broader clinical findings that, despite the elevated genome-wide mutation rate, individuals with *POLE* and *POLD1* mutations do not show an increased frequency of age-related phenotypes.

In summary, mutational signatures associated with *POLE*/*POLD1* exonuclease domain mutations were found in all cells from all tissues examined and across the three germ layers. The elevation of burden was variable among tissues, being higher in intestinal crypts and endometrial glands than in other tissues. The results from sperm indicate that the elevated mutation rate extends beyond somatic tissues into the germline.

### Mutagenesis during early embryogenesis

Somatic mutations accumulate throughout development, from the first cell division onwards^59–62^. A mutation arising in an early embryonic cell may be present in a substantial proportion of adult cells and in multiple different tissues^59^. Early embryonic mutations can be detected in whole-genome sequences of highly polyclonal adult tissue samples as mutations with relatively high VAF^59,60,62^. Using this approach, putative early embryonic mutations were identified from whole-genome sequences of whole-blood samples. The embryonic mutational spectra of some *POLE-*mutant individuals exhibited significantly larger exposures to SBS10a, SBS10b and SBS28 (*P* < 0.05, Wilcoxon rank-sum test), whereas others were dominated by SBS1 and SBS5, the signatures normally responsible for early embryonic mutations^62^ (Fig. 3c). Similarly, in *POLD1-*mutant cases, the number of early embryonic single-base pair (bp) insertions was highly elevated in some, but not all, individuals (Fig. 3d). This heterogeneity reflects the inheritance pattern of the germline mutation and is probably a consequence of the maternal to zygotic transition of gene expression^63^. When a germline *POLE*/*POLD1* mutation is paternally inherited, any effect on mutagenesis is delayed until zygotic genome activation, thus sparing the early embryo for the first few cell divisions. When maternally inherited, however, the defective proofreading polymerase is present in the cytoplasm of the ovum and *POLE*/*POLD1* mutagenesis therefore occurs immediately after fertilization, leading to a high prevalence of such mutations in early embryogenesis. These results indicate that mutagenesis due to defective *POLE*/*POLD1* proofreading is present at the earliest stages of life.

### Differential mutation burdens across the genome

**Fig. 4:**
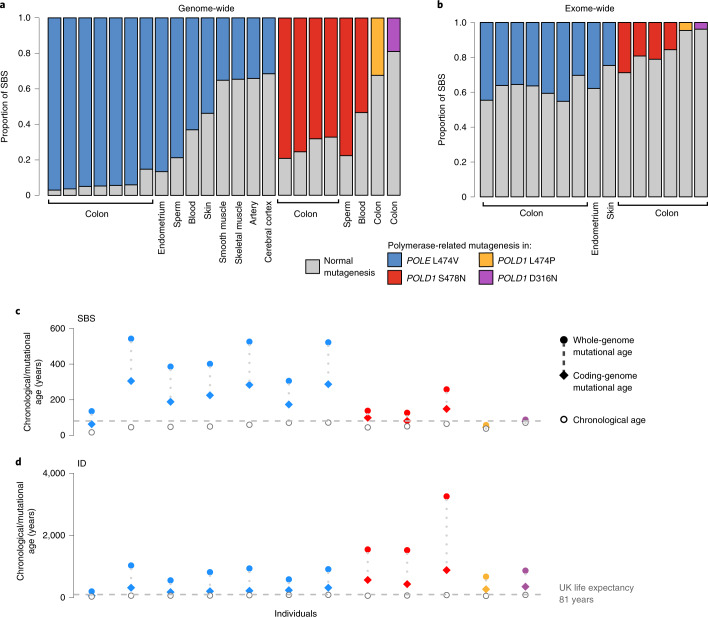
Genome- and coding-sequence-wide increase in mutation burdens. **a**, Genome-wide proportion of mutations due to *POLE* and *POLD1* germline mutations across various normal tissues. Colored bars indicate mutagenesis due to *POLE* (SBS10a, SBS10b, SBS28) or *POLD1* (SBS10c, SBS28) mutational signatures, with normal signatures in gray (SBS1, SBS5 and, for skin, SBS7a and SBS7b). **b**, Protein-coding exome proportion of mutations due to *POLE* and *POLD1* germline mutations across various normal tissues, showing a much lower increase in polymerase-related mutational signatures (Wilcoxon signed-rank test *P* = 6.1 × 10^−5^). **c**,**d**, Mutational and chronological ages of histologically normal intestinal crypts per individual. Mutational ages are calculated based on the expected rate of mutation accumulation in wild-type intestinal crypts^28^, enabling the calculation of both SBS and ID mutational age. Plots show SBS (**c**) and ID (**d**) mutational ages across the whole genome (filled dots) and coding genome (filled diamonds). Germline mutation is color coded: blue, red, green and purple denote *POLE* L424V, *POLD1 S478N, POLD1* L474P and *POLD1* D316N, respectively. Individuals’ chronological ages are indicated by unfilled circles, and UK life expectancy is displayed as a dashed horizontal line.

We compared the distribution of somatic mutations across the genome to the mutation burden in the protein-coding exome in individuals with germline *POLE*/*POLD1* mutations (Fig. 4a,b). Mutation burdens due to the various forms of SBS10 were heavily biased towards intronic, intergenic and late-replicating regions (Extended Data Fig. 7d,e), proportionately sparing protein-coding exons. This relatively constrained increase in mutation burdens in protein-coding exons may conceivably mitigate the biological consequences of elevated somatic mutation rates due to *POLE*/*POLD1* germline mutations. Nevertheless, differential burdens between tissues were maintained and mutation rates in coding regions were increased in colon and endometrium more than in skin (Fig. 4b).

## Discussion

This study shows that multiple normal cell types from *POLE*/*POLD1* exonuclease domain germline mutation carriers demonstrate the mutational signatures and elevated somatic SBS and ID mutation rates characteristic of defective proofreading by these polymerases. The results are consistent with the presence of elevated mutation rates in all cells of all types throughout life.

The extent of the elevation in mutation rate appears greater in intestinal and endometrial epithelium than in the other cell types analyzed. The basis for this variation is not understood, but may reflect different stem cell division rates. It may also, at least in part, explain the predilection for colorectal and endometrial cancer observed in these individuals.

The somatic mutation theory of aging proposes that the increasing somatic mutation burdens in normal cells, continuously accrued over a lifetime, have increasingly detrimental effects on cell function and thus engender the set of phenotypic features collectively termed aging^19–24^. The mutation burdens observed in cells from *POLE*/*POLD1* mutation carriers are higher than those in normal individuals of the same ages. Therefore, *POLE*/*POLD1* mutation carriers have elevated ‘mutational ages’ (Fig. 4c,d). The biological consequences of this generalized elevated mutational age appear, however, to be relatively limited. Other than the increase in incidence of colorectal, endometrial and other neoplasms, phenotype information from more than 100 *POLE*/*POLD1* mutation carriers does not obviously reveal features of premature aging or early onset of age-related, non-neoplastic disease, and many survive into the late decades of the standard human lifespan^16–18^ (Supplementary Table 1). Therefore, the rare natural experiment of germline *POLE*/*POLD1* exonuclease domain mutations leading to elevated mutation rates does not support a simple somatic mutation theory of aging. The results are, moreover, similar to those obtained in mice with engineered germline *POLE* and *POLD1* exonuclease domain mutations^10,11^.

Important cautions, however, should temper this conclusion. First, more comprehensive measurement of somatic mutation burdens across cell types in *POLE*/*POLD1* germline mutation carriers is indicated. The varying degrees of mutation rate elevation between cell types potentially leaves some—which could be particularly influential in generating the aging phenotype—relatively protected. Second, *POLE*/*POLD1* exonuclease domain germline mutation carriers show burdens of somatic copy number changes, rearrangements and telomere erosion similar to normal individuals (Supplementary Table 2 and Supplementary Note). If aging depends on these mutation classes, it would not be accelerated in *POLE*/*POLD1* mutation carriers. Finally, additional factors may mitigate the impact of elevated mutation burdens in *POLE*/*POLD1* exonuclease domain carriers. For example, a disproportionately small fraction of the mutation burdens due to SBS10a,b,c,d falls in coding regions of the genome, potentially reducing its biological impact^64^.

Nevertheless, the results indicate that many normal human cell types throughout life tolerate high SBS and ID mutation rates and therefore that the direct effects of somatic mutation accumulation may not underlie all components of the progressive biological dysfunction termed aging.

## Methods

### Ethical approval and study participants

This research complies with all relevant ethical regulations. Patients were recruited as part of the CORGI 2 study, United Kingdom Research Ethics Committee (REC) no. 17/SC/0079. Additional sample collection was undertaken under approval from the following committees: London, Westminster; North East, Newcastle and North Tyneside 1; and NRES Committee East of England, Cambridge South (REC nos. EC04/015, 16/NE/003 and 07-MRE05-44, respectively). Informed consent was obtained from all participants and no monetary compensation was offered for their participation. A complete list of study participants and tissue samples is summarized in Supplementary Tables 1 and 2.

### DNA extraction from bulk samples

Frozen whole blood underwent DNA extraction using the Gentra Puregene Blood Kit (Qiagen). Briefly, 1–2 ml of frozen blood was thawed, lysed in RBC lysis solution and centrifuged. Cell pellet was resuspended in cell lysis solution and incubated at 37 °C for 2 h. RNA and protein were degraded using RNase A solution and protein precipitation solution, respectively. DNA was precipitated with isopropanol, and was extracted from semen samples using β-mercaptoethanol followed by phenol chloroform extraction^65^.

### Tissue preparation

Tissues were embedded in Optimal Cutting Temperature compound, and frozen histological sections were cut at 30 µm, mounted on polyethylene naphthalate (PEN) slides and fixed in 70% ethanol for 5 min, followed by two washes with PBS for 1 min each. Slides were manually stained in hematoxylin and eosin (H&E) using a conventional staining protocol. A subset of samples (PD44594c–h and PD44589f) were fixed in PAXgene Tissue FIX (Qiagen) according to the manufacturer’s instructions. Fixed tissue samples were embedded in paraffin using a Tissue-Tek tissue-processing machine (Sakura). No formalin was used in the preparation, storage, fixation or processing of samples. Processed tissue blocks were embedded in paraffin wax, sectioned to 10-µm thickness and mounted onto PEN slides (Leica). Tissue slides were stained using a standard H&E protocol. Slides were temporarily coverslipped and scanned on a NanoZoomer S60 Slide Scanner (Hamamatsu); images were viewed using NDP.View2 software (Hamamatsu).

### Laser-capture microdissection

Laser-capture microdissection was undertaken using a LMD7000 microscope (Leica) into a skirted 96-well PCR plate. Cell lysis was undertaken using 20 µl of proteinase-K PicoPure DNA Extraction kit (Arcturus), and samples were incubated at 65 °C for 3 h followed by proteinase denaturation at 75 °C for 30 min. Thereafter samples were stored at −20 °C before DNA library preparation.

### Intestinal crypt isolation

Crypts from one tissue block (PD44593e) were isolated using EDTA chelation. In brief, dissected mucosa was incubated in an EDTA solution and gently agitated, resulting in dissociation of intestinal crypts from the underlying components of the intestinal epithelium. Crypts were then separated under a light microscope and placed in ATL buffer (Qiagen) containing 10% (v/v) proteinase K and digested overnight at 56 °C. DNA extraction was performed using the QiaAMP DNA micro kit (Qiagen) as per the manufacturer’s instructions; DNA was then stored at −20 °C.

### Low-input DNA library preparation and sequencing

DNA library preparation of microdissected tissue samples was undertaken as previously described, using a bespoke low-input enzymatic-fragmentation-based library preparation method^28,29,31,66^. This method was employed as it allows for high-quality DNA library preparation from a very low starting quantity of material (100–500 cells). DNA library concentration was assessed after library preparation and used to guide the choice of samples to take forward to DNA sequencing. Minimum library concentration was 5 ng µl^–1^, and libraries with >15 ng µl^–1^ were preferentially chosen; 150-bp, paired-end Illumina reads were prepared with Unique Dual Index barcodes (Illumina).

DNA sequencing was undertaken on a NovaSeq 6000 platform using the XP kit (Illumina). Samples were multiplexed in pools of 6–24, then sequenced to achieve a coverage of ≥30×.

### Mutation calling and postprocessing filters

Sequencing reads were aligned to NCBI human genome GRCh37 and aligned using Burrows–Wheeler alignment (BWA-MEM). SBS were called using the algorithm cancer variants through expectation maximization^67^. Mutations were called using an unmatched normal synthetic bam file to retain early embryonic and somatic mutations. Postprocessing filters were applied to remove low-input, library-preparation-specific artifacts and germline mutations using a previously described method^30,31,62,66^. Filters applied: (1) common single-nucleotide polymorphisms (SNPs) were removed by filtering against a panel of 75 unmatched normal samples^68^; (2) to remove mapping artifacts, mutations were required to have a minimum median read alignment score of mutant reads ≥140, and fewer than half of the reads supporting the mutation should be clipped (clipped median = 0); (3) a filter was utilized to remove overlapping reads resulting from relatively short insert size, which could lead to double counting of variant reads; and (4) a filter was used to remove cruciform DNA structures that can arise during the low-input library preparation method.

Next, we applied multiple filters to remove germline variants and potential artifacts whilst retaining bona fide embryonic and somatic variants. This approach has been detailed in previous publications, and the code for these filters can be found at https://github.com/TimCoorens/Unmatched_NormSeq. Mutations were aggregated per patient, and a read pile-up was performed using an in-house algorithm (cgpVAF) to tabulate the read count of mutant and reference reads per sample for each mutation locus. Germline mutations were filtered out using an exact binomial test, which is used to distinguish germline from somatic variants and utilizes aggregate read counts from all samples of the same patient^30,62^. In brief, the read depth across all samples from that individual was calculated (median in this study, ~340×). This high coverage yields a very precise estimate of the true VAF of each mutation. While the VAF estimates of the earliest embryonic single-nucleotide variants (SNVs) and germline variants from samples sequenced at 30× might overlap, VAFs from the aggregate coverage from that individual (~340×) are distinguishable using statistical testing. To achieve this, the beta-binomial test was applied using the overdispersion parameter (*ρ*) threshold for genuine variants of *ρ* > 0.1.

Phylogenetic trees were created using MPBoot (v.1.1.0 bootstrapped, 1,000) and mutations were mapped to branches using maximum-likelihood assignment.

Indels were called using Pindel^69^ utilizing the same synthetic unmatched normal sample employed in SBS mutation calling. ID calls were filtered to remove those with a quality score of <300 (‘Qual’; sum of mapping qualities of the supporting reads) and a read depth of <15. Thereafter, ID filtering was performed in a similar manner to that of SBS, to remove germline variants and library preparation/sequencing artifacts.

### Copy number alteration calling

Somatic copy number variants (CNVs) were called using the algorithm allele‐specific copy number analysis of tumors (ASCAT)^70^ (https://github.com/Crick-CancerGenomics/ascat) in the ascatNGS package^71^. Bulk (blood or, in one case, tissue) samples were used as matched normals. ASCAT was initially run with default parameters. To reduce the number of false-positive calls that arise in normal tissue samples, a segmentation penalty was applied in the ASCAT ‘aspcf’ step. Optimum performance was observed with a penalty value of 100, which was subsequently applied to all samples. Copy number calls were further filtered to remove artifacts. Copy number (CN) calls <2 MB were excluded. Samples with a goodness-of-fit of <95% were excluded. CN calls at specific recurrent breakpoints were removed. Sharing of CNVs between samples from different tissue blocks and across individuals that violated phylogenetic structures implied from SBS and ID phylogenetic trees was treated as artifactual and removed from analysis. Similarly, any recurrent copy number calls with identical breakpoints that were observed across different individuals were also removed. CNV calls were manually verified by visualization of reads in JBrowse^72^.

### Structural variant calling

Whole-genome sequences were analyzed for somatic structural variants (SVs) using the algorithm breakpoints via assembly (BRASS)^73^; paired blood samples were used as controls. If no blood sample was available, a tissue sample was used that was phylogenetically distant to the sample under analysis. SV calls were filtered using an in-house algorithm in a multistage process using bespoke software (https://github.com/MathijsSanders/AnnotateBRASS). Finally, all SV calls were manually inspected to confirm somatic variants. SV calls in L1 transposon donor regions and fragile sites were excluded from the final SV analysis.

### Mutational signature analysis

The R package HDP (https://github.com/nicolaroberts/hdp), based on the hierarchical Dirichlet process^74^, was used to extract mutational signatures. Analysis of mutational signatures using this package has previously been applied to normal tissues^28–31^. In brief, this nonparametric Bayesian method models categorical count data using the hierarchical Dirichlet process. A hierarchical structure is established using patients as the first tier (parent nodes) and individual samples as the second tier (dependent nodes). Uniform Dirichlet priors were applied across all samples. The algorithm creates a mutation catalog for each sample and infers the distribution of signatures in any one sample using a Gibbs sampler. We performed mutational signatures analysis per branch, counting each branch of the phylogenetic tree as a distinct sample to avoid double counting of mutations. Since the Markov chain Monte Carlo process scales linearly with the number of counts, we randomly subsampled each branch to a maximum of 2,500 total substitutions. Branches with <100 mutations were excluded from the mutational signature extraction. No reference signatures were included as priors.

To assess the contribution of each mutational process, mutational signatures were refitted to all mutation counts of branches of phylogenies using the R package sigfit (https://github.com/kgori/sigfit)^75^. To avoid overfitting, a limited subset of reference mutational signatures were included per patient corresponding to the HDP signatures identified in that individual. In the case of SBS10d, it was fitted only to branches in which an exposure had originally been reported.

Further details of mutational signature extraction, validation and mutational signature assignment are included in the Supplementary Note.

### Cancer driver mutations

Cancer driver mutations were identified using two methods aiming to identify genes and mutations in this cohort that are subject to positive selection. Firstly, to identify mutations in cancer genes under positive selection in an unbiased manner, we ran a modified dNdS method^76^. To avoid double counting of mutations, only unique mutations (SBS and ID) mapped to branches of the phylogenetic trees were analyzed. dndscv was run using the following parameters: max_coding_muts_per_sample=5000 and max_muts_per_gene_per_sample=20. The mutational processes associated with defective DNA polymerases have a well-reported extended sequence context bias^8,76^ that alters the expected probability of observing a mutation in specific trinucleotide nucleotide contexts. To account for this bias, a modified dNdS method was applied. Global dNdS values for the expected number of each mutation type were replaced with corrected values taking into account the observed mutation subtype (synonymous, missense, nonsynonymous and splice site) totals. A generalized negative binomial linear model was applied to each mutation subtype accounting for the biased distribution observed. *P* values were combined using Fisher’s method, and multiple testing correction was performed with the Benjamini–Hochberg method. Genes with qval < 0.05 were considered to be under positive selection.

A second phase of cancer gene mutation analysis was undertaken, identifying mutations in this cohort that are codified in cancer mutation databases and exhibit characteristic traits of cancer driver mutations, an approach previously employed in the study of normal tissues^30,31^. In this phase of the analysis we sought to identify the spectrum and frequency of cancer driver mutations in this cohort. Somatic mutations (SBS and ID) were collated per sample from all tissues. Analysis was restricted to protein-coding regions, and mutations were filtered using lists of known cancer genes; mutations in samples from intestinal epithelium were filtered using a list of 90 genes associated with colorectal cancer^28^; samples from all other tissues, including blood, were filtered using a pan-cancer list of 369 driver genes^76^. Genes were then characterized according to their predominant molecular behavior: dominant, recessive or intermediate (those demonstrating aspects of both types of behavior) using the COSMIC Cancer Gene Census^77^. All candidate mutations were annotated using the cBioportal MutationMapper database (https://www.cbioportal.org/mutation_mapper). Mutations meeting the following criteria were considered to be driver mutations: truncating mutations (those causing a shortened RNA transcript, nonsense, essential splice site, splice region and frameshift ID) in recessively acting genes; known activating hotspot mutations in dominant (and recessive) genes; and, lastly, mutations in neither of the above categories but characterized by the MutationMapper database as being ‘likely oncogenic’ were also included in the final driver mutation catalog. We also sought to compare the frequency of driver mutations in histologically normal crypts with *POLE* and *POLD1* mutations to those from individuals not carrying DNA polymerase mutations. Somatic mutations from 445 normal intestinal crypts^28^ were annotated and filtered using the above criteria. Comparison was made with normal intestinal crypts from this cohort of individuals with *POLE* and *POLD1* germline mutations (Extended Data Fig. 3).

### Embryonic variant calling

Whole-genome sequencing of bulk blood samples was used to identify early embryonic SBS and ID mutations. Since bulk blood represents a very polyclonal tissue, variants found in blood reflect those generated in the first few cell divisions of life^62^. Variant counts from blood samples were included in the germline and artifact filtering, as described above. For SBS, a minimum VAF of 0.15 was required to be included in the embryonic set. Of the remaining SBS, 205 out of a total 385 (53%) were shared with intestinal samples, confirming they must have arisen before gastrulation. For ID, we set the minimum to 0.1 to reflect the higher levels of noise accompanying indel calling and variant read counting. For indels, this amounted to 28 out of 30 (93%) mutations.

To investigate the role of *POLE* mutagenesis in the early embryo, we used the mutational signature contribution to the observed SBS counts, given the highly elevated SBS mutation rate. We fitted SBS1, SBS5, SBS10a, SBS10b and SBS28 to patient-specific embryonic counts using SigFit. SBS1 and SBS5 reflect the normal background mutagenesis already present in the embryo^60,62^, while the other signatures are caused by defective *POLE*.

For *POLD1* mutagenesis, we quantified the number of insertions of T at homopolymers of T, the characteristic peak in ID1 and the one dominating the indel landscape in patients with *POLD1*. We used insertions rather than SBS because of the relatively modest increase in SBS mutation rate, but a much higher increase in the rate of insertion acquisition.

### Modified duplex sequencing

DNA from bulk blood and sperm samples from four individuals with germline *POLE* and *POLD1* mutations was extracted as outlined above. Samples from normal healthy controls were obtained and processed using the following method. Whole blood was diluted with PBS, and mononuclear cells (MNC) were isolated using lymphoprep (STEMCELL Technologies) density gradient centrifugation. The red blood cell and granulocyte fraction of the blood was then removed. The MNC fraction was depleted of red blood cells by lytic steps involving three incubations at room temperature for 20, 10 and 10 min, respectively, with RBC lysis buffer (BioLegend). DNA was extracted from sperm samples from a 21-year-old donor.

Our modified duplex sequencing method, called NanoSeq, relies on blunt-end restriction enzymes to fragment the genome to avoid errors associated with the filling of 5' overhangs and the extension of internal nicks during end repair after sonication. Our modified method has error rates <1 × 10^–8^ (ref. ^53^).

Sperm NanoSeq libraries from *POLE*/*POLD1* mutants (median insert sizes of 289 bp) were sequenced as 150-bp paired-end reads on NovaSeq, resulting in 2.06–2.32 × 10^–8^ paired-end reads (20–23× coverage) and 2.5–3.2 × 10^9^ duplex calls (~1× effective coverage). Multiple replicates of sperm (*n* = 7) and blood/granulocyte (*n* = 6) NanoSeq libraries from healthy donors were sequenced to higher depth, resulting in 1.5–1.6 × 10^9^ paired-end reads (150–160× coverage) and 1.5–2.5 × 10^10^ bp calls (5–8× effective coverage).

Given the uneven frequencies of trinucleotides in the digested genome, the strong filtering of common SNP sites (typically occurring at CpG) and the strong dependence of mutation rates on trinucleotide contexts, our estimates of mutation burdens are normalized and projected onto genomic trinucleotide frequencies.

Let *t* denote the count of a given trinucleotide of type *i* = 1,…,32. The frequency of each trinucleotide is calculated separately for the genome $f_i^g$and for the NanoSeq experiment $f_i^e$ where:

$$f_i = \frac{{t_i}}{{\mathop {\sum }\nolimits_{i = 1}^{32} t_i}}$$

The ratio of genomic to experimental frequencies for a given trinucleotide is:

$$r_i = \frac{{f_i^g}}{{f_i^e}}$$

There are *j* = 1,…,classes of substitution where the mutated base is a pyrimidine. Let *s*_*ij*_ denote the count of substitution *j* in trinucleotide context *i*, giving a total of 96 substitution classes. Each substitution count is corrected as follows:

$$s_{ij}^\prime = s_{ij}r_i$$

The corrected substitution counts provide a substitution profile projected onto the human genome, and are also used to calculate the corrected mutation burden:

$$\beta ^\prime = \frac{{\mathop {\sum }\nolimits_{i = 1}^{32} \mathop {\sum }\nolimits_{j = 1}^6 s_{ij}^\prime }}{{\mathop {\sum }\nolimits_{i = 1}^{32} t_i}}$$

### Sequencing for ARCH-related variants in blood

Twenty-two blood samples were subjected to deep targeted sequencing (median coverage ~10,000×) using a gene panel of known drivers of clonal hematopoiesis^58^. Samples were sequenced on Illumina Hiseq4000 lanes using 75-bp paired-end reads. Sequencing reads were aligned to the human reference genome (GRCh37d5) using the BWA aligner. ShearwaterML^27^ was used to call somatic SNVs. This algorithm was developed to detect subclonal mutations in deep-sequencing data, by modeling the error rate at each site using information from the panel of normal unrelated samples. The normal panel we employ comprises data from 310 previously sequenced normal individuals (with no identifiable ARCH mutations) aged 42–89 years. Postprocessing filtering was performed as previously described, with a requirement for variants to have at least two supporting reads in both directions^27^. Germline variants were excluded by removal of those with VAF > 0.42. Probable false positives were removed by exclusion of variants with VAF < 0.005. Further filtering restricted analysis to variants causing nonsynonymous protein-coding changes or introducing a stop codon.

### Mutation burden in coding regions and mutational age calculations

The mutational ages shown in Fig. 4c,d were calculated by assessing the per-block mutation rate for normal intestinal crypts across the whole-genome and coding regions; average mutation rates were then calculated per individual for the whole-genome and coding regions. These calculations were repeated for individuals from a cohort of wild-type crypts^28^, generating an expected mutation rate for the whole-genome and coding regions. The relative increase of the observed mutation rate in individuals with DNA polymerase mutations versus wild-type crypts was used to generate mutational age.

### Telomere length estimation

Telomere attrition is a hallmark of cellular aging and is accelerated in certain disease processes. To assess the length of telomeres in the tissue samples in this cohort, we undertook estimation using two established methods from next-generation sequencing data.

Telomerecat is a ploidy-agnostic method of telomere length estimation (to bp resolution) from next-generation sequencing data that have been benchmarked across human and animal studies in normal tissues and cancers^78^. This method has been employed in previous studies of somatic mutations in normal mutations^28–30^. We generated telomere length estimates for all samples using 100 simulator runs (parameter -N100). Results for most, but not all, samples were plausible and showed a positive correlation with those from a second telomere length content algorithm (TelomereHunter). Approximately 30% of samples returned zero values for telomere length; similar observations have been made in other datasets sequenced on the Illumina NovaSeq platform. Results of the algorithm based on sequencing data generated by Illumina X10 and other sequencing platforms do not demonstrate this pattern, and can be relied upon. For this analysis we favored TelomereHunter, which is a well-established method used in tumor sequencing analyses^79^, shows good concordance with other methods of telomere length estimation^80^ and is reliable across all tested samples sequenced on the Illumina NovaSeq platform.

Telomere content measurements were generated by running TelomereHunter using default parameters across all histologically normal crypts in this cohort (*n* = 109) and normal crypts from a previous study that do not have DNA proofreading polymerase germline mutations (*n* = 445)^28^. To assess age-related telomere attrition in normal tissues, we fitted a linear mixed-effects model to assess the effect of age and also to test whether telomere attrition is greater in crypts with a DNA proofreading polymerase mutation. Age was fitted as a fixed effect and patient as a random effect; an additional dichotomous genotype variable was added as a fixed effect. We assume a similar length at birth, and thus fitted a fixed intercept and assessed the difference in slope between samples from this cohort and nonpredisposed crypts. We compared the model fit using analysis of variance and the difference between models using a chi-squared test. *P-*value thresholds of >0.05 were used (Supplementary Note)

### Research involving human gametes

Samples containing human gametes (sperm samples), collected under informed research consent from individuals enrolled in the CORGI v.2.0 study, were included in this manuscript. The CORGI v.2.0 study is approved by the United Kingdom NHS Research Ethics Committee (no. 17/SC/0079). Sperm samples were studied using genome sequencing. All experiments were conducted in accordance with the relevant international and national standards. No modification or cloning of gametes was performed.

### Reporting Summary

Further information on research design is available in the Nature Research Reporting Summary linked to this article.

## Online content

Any methods, additional references, Nature Research reporting summaries, source data, extended data, supplementary information, acknowledgements, peer review information; details of author contributions and competing interests; and statements of data and code availability are available at 10.1038/s41588-021-00930-y.

## Supplementary information

Supplementary Information Supplementary Code, Methods and Figs. 1–7.

Reporting Summary

Supplementary Tables Supplementary Table 1: Clinical summary and phenotypic characteristics of individuals with germline *POLE* and *POLE* exonuclease domain mutations. Summary of phenotypic features and disease burden in all individuals in this cohort, as well as those published to date. Supplementary Table 2: Summary of samples including DNA polymerase germline mutation, sequencing method and mutation burden. Supplementary Table 3: Cancer driver mutations identified in this cohort. Cancer driver mutations identified across all cell types studied in this cohort.

Peer Review Information

## Data Availability

DNA sequencing data are deposited in the European Genome-Phenome Archive (EGA) with accession code EGAD00001006212. DNA sequencing data from the modified duplex sequencing are deposited in the EGA with accession code EGAS00001004066. Somatic mutations and mutational signature data from this cohort are available online (https://github.com/TimCoorens/Polymerase). All other data are available from the authors on request. The cBioPortal MutationMapper database was accessed at: https://www.cbioportal.org/mutation_mapper?standaloneMutationMapperGeneTab=ATM.

## Code availability

Code required to reproduce the analyses in this paper is available online. Mutation-calling algorithms are available through GitHub (https://github.com/cancerit). All custom code used in this study is available online (https://github.com/TimCoorens/Polymerase). Code for statistical analyses is provided in Supplementary Note.
