# Supplementary material for: Increased somatic mutation burdens in normal human cells due to defective DNA polymerases
Source: Nat Genet. 2021 Sep 30;53(10):1434–42. doi: 10.1038/s41588-021-00930-y (PMC8492474; doi:10.1038/s41588-021-00930-y)
Supplement: Supplementary file 1 — Supplementary Code, Methods and Figs. 1–7. [file 41588_2021_930_MOESM1_ESM.pdf]

nature portfolio

https://doi.org/10.1038/s41588-021-00930-y

Supplementary information

Increased somatic mutation burdens in normal human cells due to defective DNA polymerases

In the format provided by the authors and unedited

SUPPLEMENTARY INFORMATION
CONTENTS

Supplementary Code

- Supplementary code and statistical analysis to accompany the manuscript

Supplementary Methods

- Mutational signature extraction
- Validation of mutational signatures
- HDP combined mutational signature extraction
- Validation and extension of mutational signature analysis
- Mutational signature assignment

Supplementary Information Fig. 1 | HDP signature components

Supplementary Information Fig. 2 | HDP signature component deconvolution

Supplementary Information Fig. 3 | Comparison of HDP and Sigprofiler signature components

Supplementary Information Fig. 4 | Cosine similarity of HDP and SigProfiler signature components

Supplementary Information Fig. 5 | HDP combined signature extraction

Supplementary Information Fig. 6 | Characterisation of mutational signature SBS91

Supplementary Information Fig. 7 | Protein structure and mutation site of POLD1 and POLE polymerases

Supplementary Code

Increased somatic mutation burdens in normal human cells due to defective DNA polymerases

Robinson et al.

24/05/2021

Contents

1.Mixed-effects linear modelling of single-base substitution (SBS) burden in non-neoplastic intestinal stem cells 4
2.Mixed-effects linear modelling of insertion / deletion (ID) burden in non-neoplastic intestinal stem cells 10
3.Hypothesis Testing - Interindividual Variability & heteroscedasticity 13
4.Assessment of telomere attrition in wild-type and DNA polymerase mutant intestinal crypts 15

This analysis has two aims: 1. To assess the rate of accumulation of SBS and ID mutation in normal intestinal crypts in individuals with DNA polymerase germline mutations 2. To assess the differences in SBS and ID mutation rates between individuals with different germline mutations

This analysis evaluates the rate of accumulation of SBS and ID mutations in histologically normal crypts from individuals with germline mutations in POLE and POLD1 exonuclease domains and compares them to individuals with no germline predisposition (Lee-Six et al 2019). Studies in experimental systems inducing hypermutator phenotypes have shown that different germline mutations in the same gene can alter the rate of accumulation of mutations. Consistent with this observation we will model the accumulation of mutations in normal crypts from individuals with germline polymerase mutations allowing for different mutation rates in each grouping. We categorise data into three groups: POLE L424V n=7, POLD1 S478N n=4, POLD1other (L474P and D316N) n=2.

Analysis from these data and evidence from studies in mice suggest that the mutational processes of defective DNA polymerases are continuous and accumulate linearly with age. We therefore seek as a primary aim to define the differences in the rate of accumulation of mutations due to the underlying mutational processes that result from these germline mutations.

Model parameters encoded are: 1. Age - chronological age in years from birth at the time of tissue sampling 2. Site - anatomical site (small or large bowel) 3. Germline Mutation - this is a categorical variable that encodes WT, POLD1 and POLE exonuclease domain mutation carriers 4. Patient - this is encoded as a random effect interacting with germline mutation

To better understand the degree to which the SBS rate is elevated in affected individuals, we include whole genome sequencing data from a cohort of 445 normal intestinal crypts from a previously published study (Lee-Six et al 2019). There are two methodological differences between the current and previous study; 1. Sequencing coverage was greater in current study (wild-type (WT) cohort ~17x median of the POLE/D1 cohort ~33.5x), 2. Mutation filtering was slightly different. To resolve these differences we have; 1. normalised the mutation burden in all crypts according to the sensitivity in each sample (a function of median coverage and median VAF) 2. re-filtered the WT cohort data using the same approach adopted in the current study (Methods). Since we correct for coverage before modelling, we do not include it as a parameter for modelling.

First we load and explore the data and its categorisation.

```r
suppressWarnings(library(nlme))
suppressWarnings(library(MuMIn))
options(stringsAsFactors = F)

df_all <- read.delim("mutation_rate_summary2020-06-09.txt",
    header = T, sep = "")
df_all$germline_mutation[df_all$patient %in% c("PD44588",
    "PD44590")] <- "POLD1other"
df_all$germline_mutation[df_all$germline_mutation == "POLD1"] <- "POLD1S478N"
df_all$sitenew = ifelse(df_all$site %in% c("right", "left",
    "transverse"), "large", "small")
```

```r
df_all$sitenew <- factor(df_all$sitenew, c("large", "small"))
df_all$germline_mutation <- factor(df_all$germline_mutation,
    levels = c("WT", "POLE", "POLD1S478N", "POLD1other"))
df_all$coverage <- scale(df_all$coverage)
df_all <- df_all[df_all$patient != "O337", ]  #Very high chemotherapy related mutation burden
head(df_all)
```

```
##         sample patient age germline_mutation  site sbstotal indeltotal
## 1 HLS_1C_30_B5     HLS  60                WT right     4734        218
## 2 HLS_1C_30_D5     HLS  60                WT right     4306        158
## 3 HLS_1C_30_G5     HLS  60                WT right     3959        188
## 4 HLS_1C_30_H5     HLS  60                WT right     3627        115
## 5 HLS_2C_30_D6     HLS  60                WT right     3790        124
## 6 HLS_2C_30_E6     HLS  60                WT right     4444        163
##   median_vaf median_indelvaf    coverage sensitivity sbstotal_corr
## 1     0.4167          0.4615 -0.08931167   0.9554499      4954.734
## 2     0.3889          0.4348 -0.47757499   0.8659166      4972.766
## 3     0.4091          0.4286 -0.12863389   0.9460467      4184.783
## 4     0.4286          0.5238 -0.74982461   0.8382143      4327.056
## 5     0.4545          0.5263 -0.84017848   0.8410742      4506.142
## 6     0.4375          0.5000 -0.65287957   0.8778454      5062.395
##   sensitivity_indel indeltotal_corr sitenew
## 1         0.9935051        219.4251   large
## 2         0.9832130        160.6976   large
## 3         0.9865349        190.5660   large
## 4         0.9968977        115.3579   large
## 5         0.9968893        124.3869   large
## 6         0.9950350        163.8133   large
```

The normal intestinal stem cells studied in this cohort are from different anatomical sites including the small and large intestine. We see small differences in the mutation rate between these sites in WT intestinal crypts and observe different mutational processes (Lee-Six et al 2019). Furthermore individuals with germline polymerase mutations are predisposed to both large and small bowel tumours albeit clinical data suggests at different rates. Since we have limited numbers of small bowel crypts in this cohort we are interested in inclusion in the model to improve fit but are cautious about interpretation of any differences that a model may identify. Below are tables summarising the number of individual crypts represented across different anatomical sites grouped by germline mutation. For the purpose of modelling sites have been grouped into large and small intestine.

```r
table(df_all$germline_mutation, df_all$site)
```

```
##
##              duodenum ileum left right transverse
##   WT                0    21  119   159        134
##   POLE             14    10   21     4         16
##   POLD1S478N        0     0   15     6          6
##   POLD1other        0     0   11     3          3
```

```r
table(df_all$germline_mutation, df_all$sitenew)
```

```
##
##              large small
##   WT           412    21
##   POLE          41    24
##   POLD1S478N    27     0
##   POLD1other    17     0
```

Examining the raw-data with linear regression lines fitted, we can see evidence of increasing mutation burden with age and

differential mutation rates in crypts with the various germline mutations. ID mutation rates appear to differ between individuals with the POLD1 S478N, given the sparsity of the data it is challenging to fit a simple linear regression. We can however make a conservative assumption that mutation burden in the fertilised embryo (time point 0-9mths) is 0 and that it will be modestly higher in the intestinal stem cells at birth. When we fit the mixed effects model we assume the intercept passes near to / through the origin.

```
colvec = c(WT = "black", POLE = "dodgerblue", POLD1S478N = "red",
    POLD1other = "magenta")
plot(df_all$age, df_all$sbstotal_corr, col = colvec[df_all$germline_mutation],
    xlim = c(0, 80), xlab = "age (yrs)", ylab = "SBS mutations")
abline(lm(df_all$sbstotal_corr[df_all$germline_mutation ==
    "WT"] ~ df_all$age[df_all$germline_mutation == "WT"]),
    col = colvec["WT"])
abline(lm(df_all$sbstotal_corr[df_all$germline_mutation ==
    "POLE"] ~ df_all$age[df_all$germline_mutation == "POLE"]),
    col = colvec["POLE"])
abline(lm(df_all$sbstotal_corr[df_all$germline_mutation ==
    "POLD1S478N"] ~ df_all$age[df_all$germline_mutation ==
    "POLD1S478N"]), col = colvec["POLD1S478N"])
abline(lm(df_all$sbstotal_corr[df_all$germline_mutation ==
    "POLD1other"] ~ df_all$age[df_all$germline_mutation ==
    "POLD1other"]), col = colvec["POLD1other"])
legend("topleft", legend = c("WT", "POLE", "POLD1S478N",
    "POLD1other"), col = colvec, bty = "n", lty = 1, lwd = 3)
```

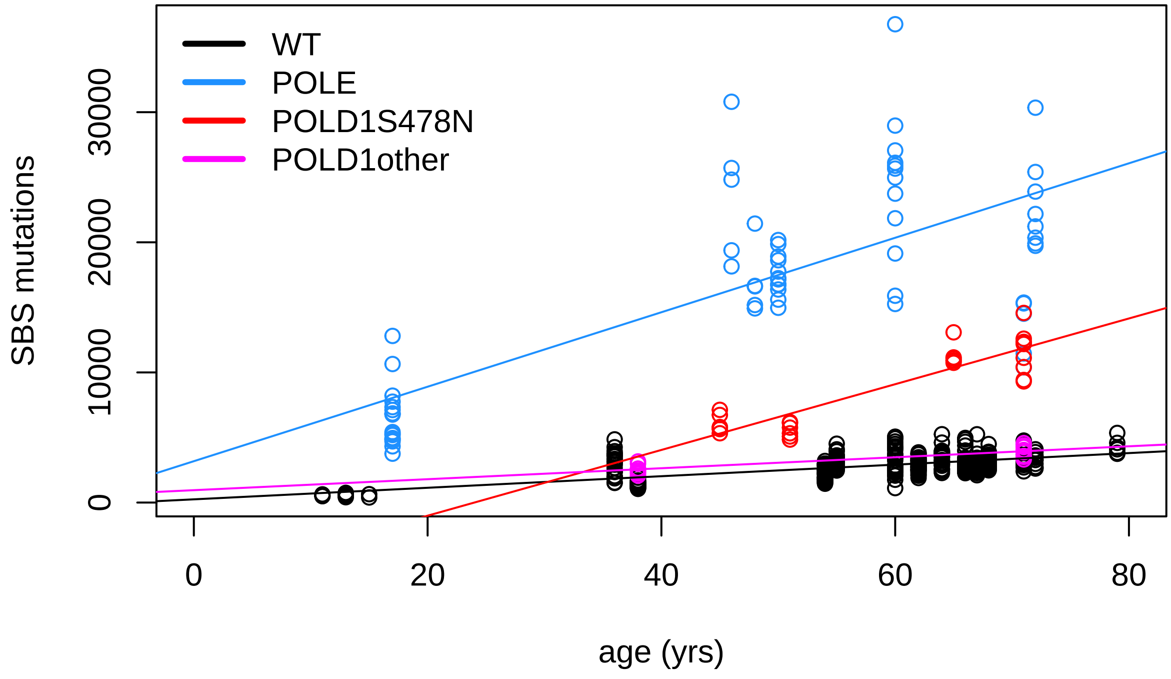


```
plot(df_all$age, df_all$indeltotal_corr, col = colvec[df_all$germline_mutation],
    xlim = c(0, 80), xlab = "age", ylab = "ID mutations")
abline(lm(df_all$indeltotal_corr[df_all$germline_mutation ==
    "WT"] ~ df_all$age[df_all$germline_mutation == "WT"]),
    col = colvec["WT"])
abline(lm(df_all$indeltotal_corr[df_all$germline_mutation ==
    "POLE"] ~ df_all$age[df_all$germline_mutation == "POLE"]),
    col = colvec["POLE"])
```

```r
abline(lm(df_all$indeltotal_corr[df_all$germline_mutation ==
    "POLD1S478N"] ~ df_all$age[df_all$germline_mutation ==
    "POLD1S478N"]), col = colvec["POLD1S478N"])
abline(lm(df_all$indeltotal_corr[df_all$germline_mutation ==
    "POLD1other"] ~ df_all$age[df_all$germline_mutation ==
    "POLD1other"]), col = colvec["POLD1other"])
legend("topleft", legend = c("WT", "POLE", "POLD1S478N",
    "POLD1other"), col = colvec, bty = "n", lty = 1, lwd = 3)
```

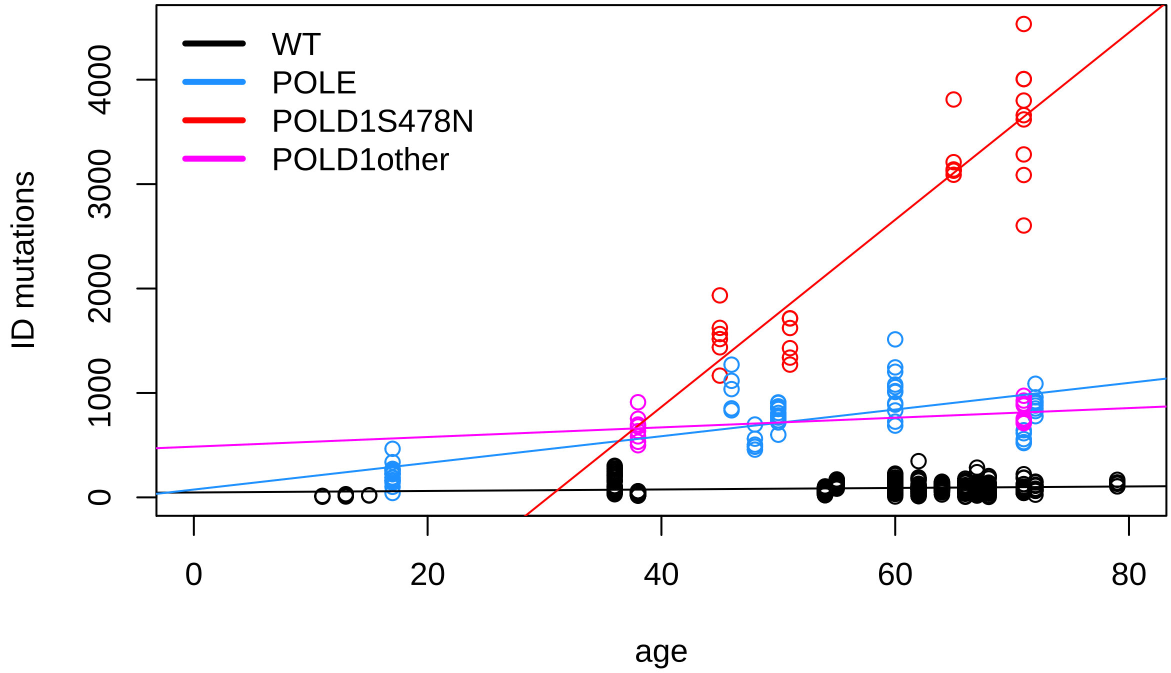

1.Mixed-effects linear modelling of single-base substitution (SBS) burden in non-neoplastic intestinal stem cells

Null model including age as the main function of sbstotal.

```r
model.age <- model.age <- lme(fixed = sbstotal_corr ~ age,
    random = list(patient = pdSymm(form = ~1)), data = df_all,
    method = "ML")

summary(model.age)
```

```
## Linear mixed-effects model fit by maximum likelihood
##  Data: df_all
##        AIC      BIC   logLik
##   9614.621 9631.802 -4803.31
##
## Random effects:
##  Formula: ~1 | patient
##         (Intercept) Residual
## StdDev:    5588.096 1337.055
##
## Fixed effects: sbstotal_corr ~ age
##                 Value Std.Error  DF   t-value p-value
```

```
## (Intercept) 3912.524 2996.1511 488 1.3058500  0.1922
## age           24.104   49.4751  52 0.4871981  0.6282
##  Correlation:
##     (Intr)
## age -0.967
##
## Standardized Within-Group Residuals:
##         Min          Q1         Med          Q3         Max
## -6.67538324 -0.24936262 -0.02519256  0.21787940  9.40384317
##
## Number of Observations: 542
## Number of Groups: 54
```

```r
intervals(model.age, which = "fixed")
```

```
## Approximate 95% confidence intervals
##
##  Fixed effects:
##                   lower       est.     upper
## (Intercept) -1963.55319 3912.52401 9788.6012
## age           -74.99147   24.10416  123.1998
## attr(,"label")
## [1] "Fixed effects:"
```

```r
model = model.age
plot(model$fitted[, 1], resid(model), xlab = "fitted", ylab = "residual")
abline(h = 0, lty = 3)
```

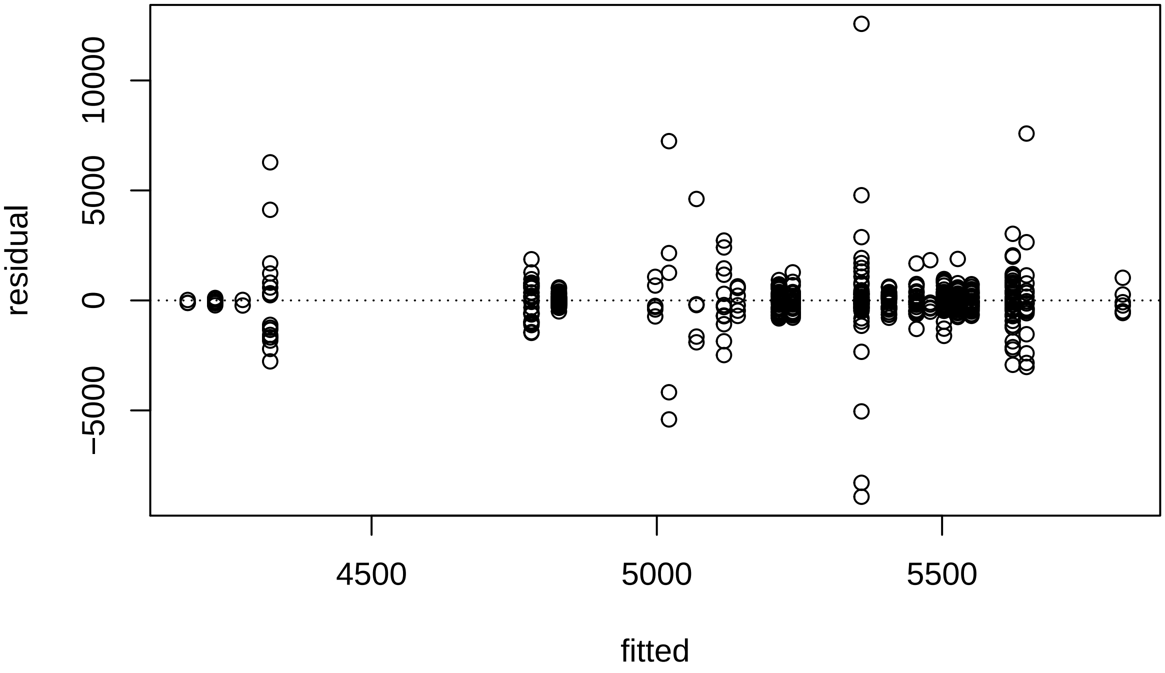

This null model has a very poor fit. Introduction of additional parameters, specifically the germline mutation may help to improve this.

Second model including an interaction term between age and germline mutation, assuming a continuous increase in SBS burden throughout life. The accumulation of mutations in intestinal stem cells is by default assumed to be linear based on data from Lee-Six et al 2019. This model provides a reasonable fit to the data.

```r
model.age.int.germ <- lme(fixed = sbstotal_corr ~ age:germline_mutation -
    1, random = list(patient = pdSymm(form = ~germline_mutation -
    1)), weights = varIdent(form = ~1 | germline_mutation),
    data = df_all, method = "ML")

summary(model.age.int.germ)
```

```
## Linear mixed-effects model fit by maximum likelihood
##  Data: df_all
##        AIC      BIC    logLik
##   8719.527 8796.842 -4341.763
##
## Random effects:
##  Formula: ~germline_mutation - 1 | patient
##  Structure: General positive-definite
##                               StdDev    Corr
## germline_mutationWT           502.7683 grm_WT g_POLE g_POLD
## germline_mutationPOLE        5023.4344  0.009
## germline_mutationPOLD1S478N 1341.0151  0.000  0.000
## germline_mutationPOLD1other  237.0724  0.000 -0.008  0.000
## Residual                     495.6003
##
## Variance function:
##  Structure: Different standard deviations per stratum
##  Formula: ~1 | germline_mutation
##  Parameter estimates:
##         WT       POLE POLD1S478N POLD1other
##  1.0000000  7.2326501  2.3315286  0.8028155
## Fixed effects: sbstotal_corr ~ age:germline_mutation - 1
##                                  Value Std.Error DF  t-value p-value
## age:germline_mutationWT          49.1491   1.36305 50 36.05812       0
## age:germline_mutationPOLE       331.1969  36.02109 50  9.19453       0
## age:germline_mutationPOLD1S478N 152.2589  12.02297 50 12.66400       0
## age:germline_mutationPOLD1other  58.0833   3.39675 50 17.09967       0
##  Correlation:
##                                 ag:_WT a:_POLE a:_POLD1S
## age:germline_mutationPOLE       0
## age:germline_mutationPOLD1S478N 0      0
## age:germline_mutationPOLD1other 0      0       0
##
## Standardized Within-Group Residuals:
##         Min          Q1         Med          Q3         Max
## -3.08905548 -0.53617340 -0.04966287  0.45715200  3.85996474
##
## Number of Observations: 542
## Number of Groups: 54
```

```r
intervals(model.age.int.germ, which = "fixed")
```

```
## Approximate 95% confidence intervals
##
##  Fixed effects:
##                                     lower      est.     upper
## age:germline_mutationWT          46.42144  49.14909  51.87674
```

```
## age:germline_mutationPOLE       259.11390 331.19692 403.27995
## age:germline_mutationPOLD1S478N 128.19933 152.25890 176.31846
## age:germline_mutationPOLD1other  51.28598  58.08333  64.88069
## attr(,"label")
## [1] "Fixed effects:"
```

```
model = model.age.int.germ
plot(model$fitted[, 1], resid(model), xlab = "fitted", ylab = "residual")
abline(h = 0, lty = 3)
```

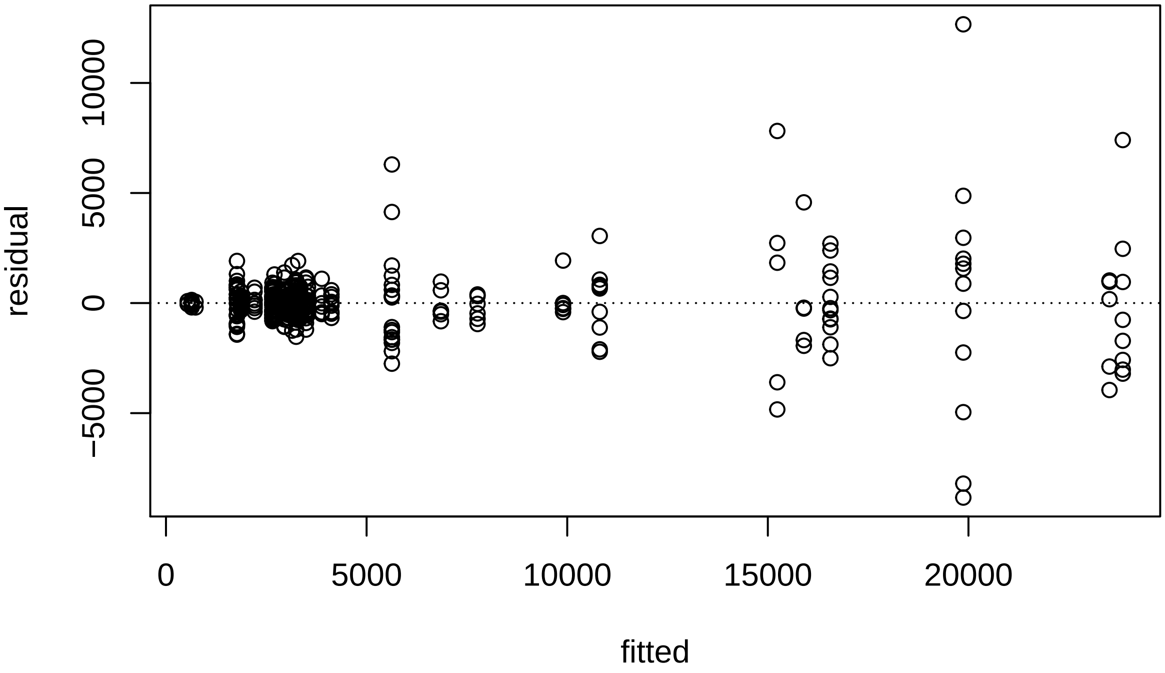

The second model appears to have a good fit to the data and there is no clear shape to the residuals, suggesting that we have introduced an appropriate number of parameters in the model. There is however some variance at higher fitted values suggesting that there may be some inter-individual and intra-individual heterogeneity that is not accounted for by the age and germline mutation. Inter-individual heterogeneity is modeled later on in this document.

Now we compare the model fit of the first (null) model as defined at the top of this section and the second, more complex model that includes germline_mutation as a grouping variable, fixed effect and interaction term with age.

```
anova(model.age, model.age.int.germ)
```

```
##                    Model df      AIC      BIC    logLik   Test  L.Ratio
## model.age              1  4 9614.621 9631.802 -4803.310
## model.age.int.germ     2 18 8719.527 8796.842 -4341.763 1 vs 2 923.0939
##                    p-value
## model.age
## model.age.int.germ  <.0001
```

Inclusion of an interaction between age and germline_mutation results in a more complex model with a statistically significant improvement in model fit.

So far we have not considered the impact of anatomical site on mutation burden and whether inclusion of this parameter can improve model fit. We know that the mutational processes in small and large bowel are different and that small bowel tumours are less common in individuals with germline polymerase mutations. We therefore wish to assess whether inclusion of site as a fixed effect improves the model.

```r
model.age.int.germ.site <- lme(fixed = sbstotal_corr ~ age:germline_mutation -
    1 + sitenew, random = list(patient = pdSymm(form = ~germline_mutation -
    1)), weights = varIdent(form = ~1 | germline_mutation),
    data = df_all, method = "ML")

summary(model.age.int.germ.site)
```

```
## Linear mixed-effects model fit by maximum likelihood
##  Data: df_all
##        AIC      BIC    logLik
##   8674.35 8760.255 -4317.175
##
## Random effects:
##  Formula: ~germline_mutation - 1 | patient
##  Structure: General positive-definite
##                              StdDev       Corr
## germline_mutationWT           524.9251748 grm_WT g_POLE g_POLD
## germline_mutationPOLE        4986.2363854 0.197
## germline_mutationPOLD1S478N 1432.5220988 0.000  0.000
## germline_mutationPOLD1other    0.1683105 0.000  0.043  0.017
## Residual                      466.3767117
##
## Variance function:
##  Structure: Different standard deviations per stratum
##  Formula: ~1 | germline_mutation
##  Parameter estimates:
##         WT       POLE POLD1S478N POLD1other
##  1.0000000  7.6439065  2.4773611  0.8277718
## Fixed effects: sbstotal_corr ~ age:germline_mutation - 1 + sitenew
##                                 Value Std.Error  DF   t-value p-value
## sitenewlarge                 609.6018 245.13202 487  2.486830  0.0132
## sitenewsmall                -228.8592 270.88514 487 -0.844857  0.3986
## age:germline_mutationWT       39.8067   4.09702  50  9.716004  0.0000
## age:germline_mutationPOLE    323.1615  36.07783  50  8.957344  0.0000
## age:germline_mutationPOLD1S478N  142.0805  13.42620  50 10.582333  0.0000
## age:germline_mutationPOLD1other   47.8132   4.37491  50 10.928958  0.0000
##  Correlation:
##                                 stnwlr stnwsm ag:_WT a:_POLE a:_POLD1S
## sitenewsmall                     0.898
## age:germline_mutationWT         -0.939 -0.847
## age:germline_mutationPOLE       -0.118 -0.110  0.110
## age:germline_mutationPOLD1S478N -0.304 -0.273  0.286  0.036
## age:germline_mutationPOLD1other -0.928 -0.834  0.872  0.109   0.282
##
## Standardized Within-Group Residuals:
##         Min          Q1         Med          Q3         Max
## -3.30809084 -0.53107172 -0.05940079  0.44902346  4.10050432
##
## Number of Observations: 542
## Number of Groups: 54
```

```r
intervals(model.age.int.germ.site, which = "fixed")
```

```
## Approximate 95% confidence intervals
```

```
##
##  Fixed effects:
##                                        lower        est.       upper
## sitenewlarge                        130.62820  609.60178 1088.57536
## sitenewsmall                       -758.15280 -228.85915  300.43450
## age:germline_mutationWT              31.62323   39.80666   47.99009
## age:germline_mutationPOLE           251.09926  323.16150  395.22373
## age:germline_mutationPOLD1S478N     115.26289  142.08053  168.89817
## age:germline_mutationPOLD1other      39.07471   47.81320   56.55169
## attr(,"label")
## [1] "Fixed effects:"
```

```r
model = model.age.int.germ.site
plot(model$fitted[, 1], resid(model), xlab = "fitted", ylab = "residual")
abline(h = 0, lty = 3)
```

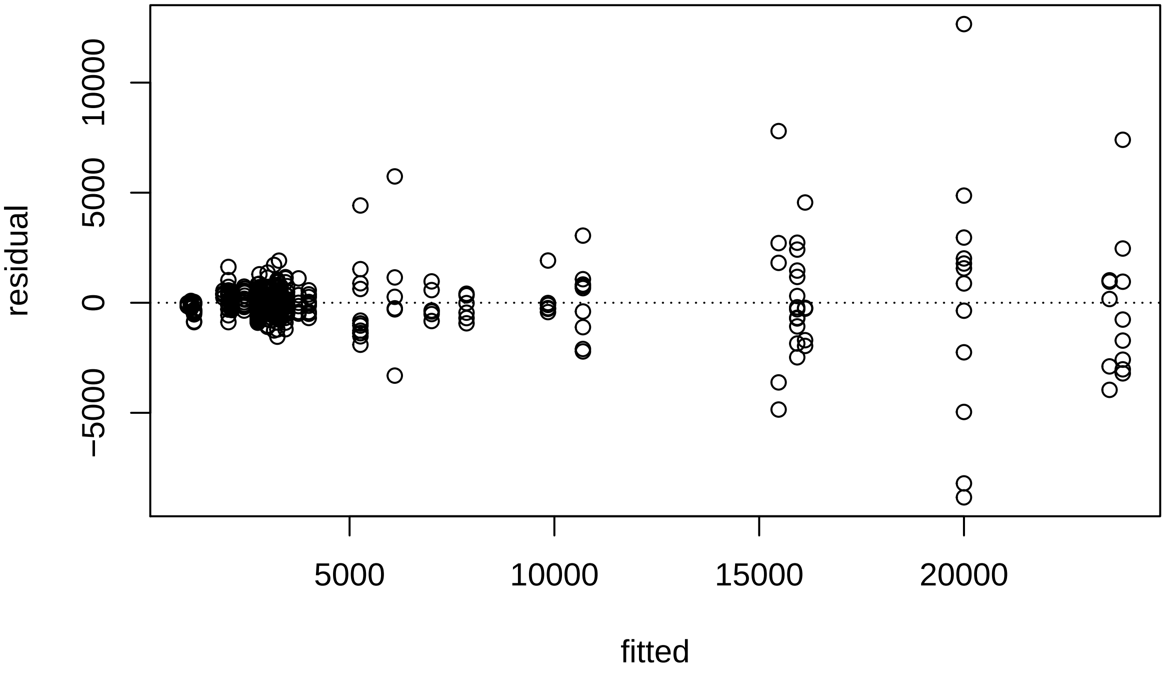

The addition of the anatomical site results demonstrates that small bowel crypts have fewer mutations than those from the large bowel when allowing for differences in age and germline_mutation. However, the effect size intervals are very broad and the p-value is non-significant. Inclusion of site as a fixed effect does however result in a statistically significant improvement in model fit. The very large effect intervals suggest that it is not necessarily an optimum fit. The simpler model which does not include site, appears to provide an acceptable model fit. We therefore proceed with model 2 (model.age.int.germ) as the default model.

```r
anova(model.age.int.germ, model.age.int.germ.site)
```

```
##                         Model df      AIC      BIC    logLik   Test
## model.age.int.germ          1 18 8719.527 8796.842 -4341.763
## model.age.int.germ.site     2 20 8674.350 8760.255 -4317.175 1 vs 2
##                          L.Ratio p-value
## model.age.int.germ
## model.age.int.germ.site 49.17695  <.0001
```

```r
print(paste0(" p-value = ", anova(model.age.int.germ, model.age.int.germ.site,
    test = T)$"p-value"[2]))
```

```
## [1] " p-value = 2.09585164651507e-11"
```

Visualisation of the interval / effect sizes of our default model

```
int <- intervals(model.age.int.germ, which = "fixed")
kf <- dim(int$fixed)[1]
par(oma = c(1, 4, 1, 1))
plot(int$fixed[, 2], kf:1, xlab = "SBS/year", ylab = "",
    xlim = c(0, 400), axes = F, col = colvec, lwd = 2)
axis(1, at = c("0", "100", "200", "300", "400"))
axis(2, kf:1, c("WT", "POLE", "POLD1 S478N", "POLD1other"),
    las = 2)
segments(int$fixed[, 1], kf:1, int$fixed[, 3], kf:1)
abline(v = 0)
abline(v = int$fixed[, 2], lty = 2, col = "grey")
```

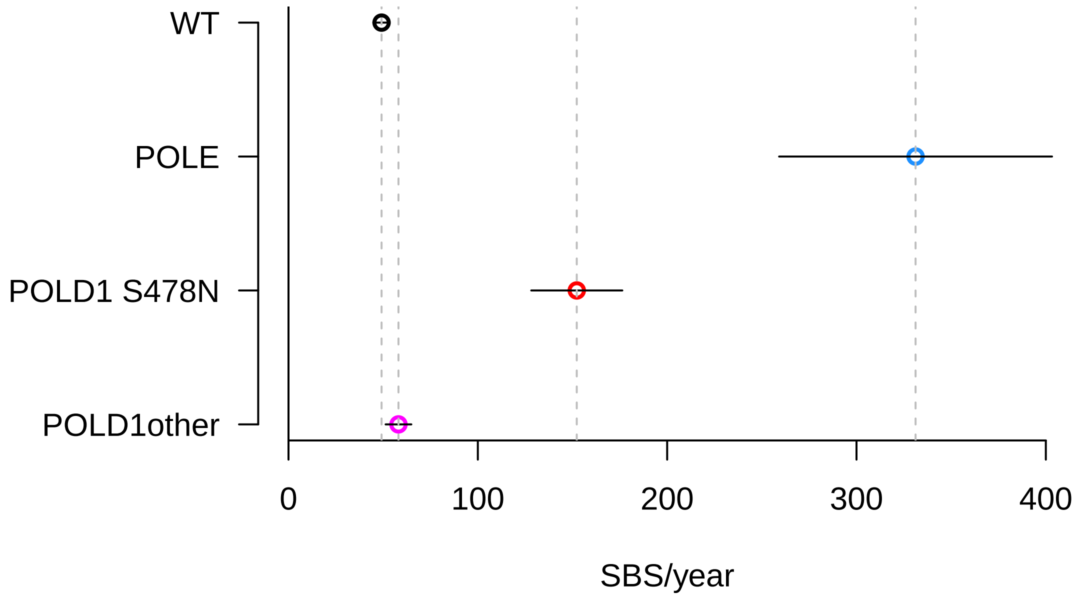

We accept the model "model.age.int.germ" as the default model and will use this to undertake hypothesis testing.

Finally we test the proportion of the data which is explained by the accepted model. Both the fixed effects (as indicated by the marginal value) and the combination of fixed and random effects (as indicated by the conditional value) show good R2 values for the fixed and combined fixed and random effects.

```
r.squaredGLMM(model.age.int.germ)
```

```
## Warning: 'r.squaredGLMM' now calculates a revised statistic. See the help
## page.

##            R2m       R2c
## [1,] 0.8686718 0.9909524
```

2.Mixed-effects linear modelling of insertion / deletion (ID) burden in non-neoplastic intestinal stem cells

We then explore the above models and their fit in the indel burden (ID) data.

First we explore a very simple model including age alone as a fixed effect.

```
model.indel.age <- lme(fixed = indeltotal_corr ~ age, random = list(patient = pdSymm(form = ~1)),
    weights = varIdent(form = ~1), data = df_all, method = "ML")
```

```r
summary(model.indel.age)
```

```
## Linear mixed-effects model fit by maximum likelihood
##  Data: df_all
##        AIC      BIC    logLik
##   6907.285 6924.466 -3449.642
##
## Random effects:
##  Formula: ~1 | patient
##         (Intercept) Residual
## StdDev:    698.4433 105.0795
##
## Fixed effects: indeltotal_corr ~ age
##                 Value Std.Error  DF   t-value p-value
## (Intercept) 224.86966  373.0887 488 0.6027244   0.547
## age           2.54717    6.1611  52 0.4134252   0.681
##  Correlation:
##     (Intr)
## age -0.967
##
## Standardized Within-Group Residuals:
##         Min          Q1         Med          Q3         Max
## -9.61858116 -0.24918020 -0.01492432  0.21505062  8.74696591
##
## Number of Observations: 542
## Number of Groups: 54
```

This model appears to lack the complexity to describe the data. Next we include an interaction term with germline mutation.

```r
model.indel.age.int.germ <- lme(fixed = indeltotal_corr ~
    age:germline_mutation, random = list(patient = pdSymm(form = ~germline_mutation -
    1)), weights = varIdent(form = ~1 | germline_mutation),
    data = df_all, method = "ML")

summary(model.indel.age.int.germ)
```

```
## Linear mixed-effects model fit by maximum likelihood
##  Data: df_all
##        AIC      BIC    logLik
##   6007.654 6089.264 -2984.827
##
## Random effects:
##  Formula: ~germline_mutation - 1 | patient
##  Structure: General positive-definite
##                              StdDev   Corr
## germline_mutationWT           35.0336 grm_WT g_POLE g_POLD
## germline_mutationPOLE        221.4797  0.000
## germline_mutationPOLD1S478N 512.4891  0.097  0.000
## germline_mutationPOLD1other 125.2573 -0.001  0.000  0.000
## Residual                      39.8169
##
## Variance function:
##  Structure: Different standard deviations per stratum
##  Formula: ~1 | germline_mutation
```

```
##  Parameter estimates:
##        WT      POLE POLD1S478N POLD1other
##  1.000000  3.423933  9.771265  2.966312
## Fixed effects: indeltotal_corr ~ age:germline_mutation
##                                   Value Std.Error  DF  t-value p-value
## (Intercept)                    30.06818 25.424700 488 1.182636  0.2375
## age:germline_mutationWT         1.02182  0.410888  49 2.486862  0.0163
## age:germline_mutationPOLE      12.95958  1.636106  49 7.920994  0.0000
## age:germline_mutationPOLD1S478N 43.84728  4.568253  49 9.598260  0.0000
## age:germline_mutationPOLD1other 12.26740  1.694656  49 7.238874  0.0000
##  Correlation:
##                                (Intr) ag:_WT a:_POLE a:_POLD1S
## age:germline_mutationWT         -0.972
## age:germline_mutationPOLE       -0.269  0.262
## age:germline_mutationPOLD1S478N -0.093  0.090  0.025
## age:germline_mutationPOLD1other -0.252  0.245  0.068   0.023
##
## Standardized Within-Group Residuals:
##         Min          Q1         Med          Q3         Max
## -3.20145516 -0.51395624 -0.04788394  0.46008182  4.54512306
##
## Number of Observations: 542
## Number of Groups: 54
```

Comparison of the first and second models demonstrates much improved model fit with the inclusion of a germline mutation interaction effect.

```r
anova(model.indel.age, model.indel.age.int.germ)
```

```
##                          Model df      AIC      BIC    logLik   Test
## model.indel.age              1  4 6907.285 6924.466 -3449.642
## model.indel.age.int.germ     2 19 6007.654 6089.264 -2984.827 1 vs 2
##                          L.Ratio p-value
## model.indel.age
## model.indel.age.int.germ 929.631  <.0001
```

```r
print(paste0(" p-value = ", anova(model.indel.age, model.indel.age.int.germ,
    test = T)$"p-value"[2]))
```

```
## [1] " p-value = 1.60137027168589e-188"
```

Effect sizes and accompanying 95% confidence intervals are displayed below:

```r
int <- intervals(model.indel.age.int.germ, which = "fixed")
int <- as.data.frame(int$fixed)
int = int[-1, ]
kf <- nrow(int)
par(oma = c(1, 4, 1, 1))
plot(int[, 2], kf:1, xlab = "ID/year", ylab = "", xlim = c(0,
    50), axes = F, col = colvec, lwd = 2)
axis(1, at = c("0", "10", "20", "30", "40", "50"))
axis(2, at = 4:1, labels = c("WT", "POLE", "POLD1S478N",
    "POLD1other"), las = 2)
segments(int[, 1], kf:1, int[, 3], kf:1)
abline(v = 0)
abline(v = int[, 2], lty = 2, col = "grey")
```

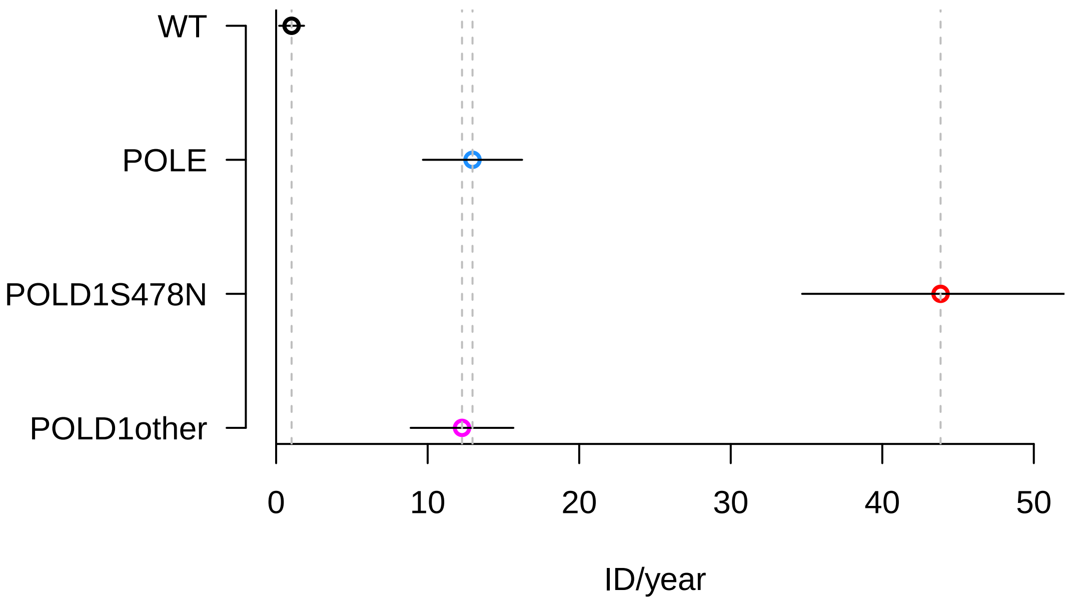

Finally we test the proportion of the data which is explained by the accepted model. Both the fixed effects (as indicated by the marginal value) and the combination of fixed and random effects (as indicated by the conditional value) show good R^2 values for the fixed and combined fixed and random effects.

```
r.squaredGLMM(model.indel.age.int.germ)
```

```
##            R2m       R2c
## [1,] 0.9404094 0.9957105
```

3.Hypothesis Testing - Interindividual Variability & heteroscedasticity

Here we undertake testing of two key hypotheses 1. Assessment of inter-individual variability in the cohort 2. Assessment of heteroscedasticity

```
# INTERINDIVIDUAL VARIABILITY - WHOLE COHORT

model.age.int.germ <- lme(fixed = sbstotal_corr ~ age:germline_mutation -
    1, random = list(patient = pdSymm(form = ~germline_mutation -
    1)), weights = varIdent(form = ~1 | germline_mutation),
    data = df_all, method = "ML")

model.age.int.germ.minus.btpt.het <- lme(fixed = sbstotal_corr ~
    age:germline_mutation - 1, random = list(patient = pdSymm(form = ~1)),
    weights = varIdent(form = ~1 | germline_mutation), data = df_all,
    method = "ML")

anova(model.age.int.germ.minus.btpt.het, model.age.int.germ)
```

```
##                                   Model df      AIC      BIC    logLik
## model.age.int.germ.minus.btpt.het     1  9 8739.284 8777.941 -4360.642
## model.age.int.germ                    2 18 8719.527 8796.842 -4341.763
##                                     Test  L.Ratio p-value
## model.age.int.germ.minus.btpt.het
## model.age.int.germ                1 vs 2 37.75659  <.0001
```

The above suggests that allowance for inter-individual heterogeneity is improving our model. Removal of this results in a worse model fit. The difference in model fit is statistically significant.

Next we test whether the inter-individual heterogeneity still exists with the WT cohort removed.

```
# INTERINDIVIDUAL VARIABILITY - REMOVAL OF WT COHORT

df_no_WT <- df_all[df_all$germline_mutation != "WT", ]


model.age.int.germ.with.btpt.het.no.WT <- lme(fixed = sbstotal_corr ~
    age:germline_mutation - 1, random = list(patient = pdSymm(form = ~germline_mutation -
    1)), weights = varIdent(form = ~1 | germline_mutation),
    data = df_no_WT, method = "ML")


model.age.int.germ.minus.btpt.het.no.WT <- lme(fixed = sbstotal_corr ~
    age:germline_mutation - 1, random = list(patient = pdSymm(form = ~1)),
    weights = varIdent(form = ~1 | germline_mutation), data = df_no_WT,
    method = "ML")


anova(model.age.int.germ.with.btpt.het.no.WT, model.age.int.germ.minus.btpt.het.no.WT)
```

```
##                                          Model df      AIC      BIC
## model.age.int.germ.with.btpt.het.no.WT       1 12 2013.578 2045.874
## model.age.int.germ.minus.btpt.het.no.WT      2  7 2017.457 2036.296
##                                             logLik   Test  L.Ratio p-value
## model.age.int.germ.with.btpt.het.no.WT   -994.7891
## model.age.int.germ.minus.btpt.het.no.WT -1001.7285 1 vs 2 13.87872  0.0164
```

When we exclude WT individuals from the analysis, there is a marginal (but non-significant) improvement in model fit in the model that incorporarates interindividual variability. This suggests that inter-individual heterogeneity may be occurring in polymerase mutant groupings and WT cohorts.

Next, we assess whether within-individual variability is different in individuals with polymerase mutations compared to those without i.e. testing for heteroscedasticity.

```
model.age.coverge.minus.heteroscedasticity <- lme(fixed = sbstotal_corr ~
    age, random = list(patient = pdSymm(form = ~germline_mutation -
    1)), data = df_all, method = "ML")

anova(model.age.coverge.minus.heteroscedasticity, model.age.int.germ,
    test = TRUE)
```

```
##                                            Model df      AIC      BIC
## model.age.coverge.minus.heteroscedasticity     1 13 9450.787 9506.625
## model.age.int.germ                             2 18 8719.527 8796.842
##                                               logLik   Test  L.Ratio
## model.age.coverge.minus.heteroscedasticity -4712.394
## model.age.int.germ                         -4341.763 1 vs 2 741.2601
##                                            p-value
## model.age.coverge.minus.heteroscedasticity
## model.age.int.germ                          <.0001
```

```
print(paste0(" p-value = ", anova(model.age.coverge.minus.heteroscedasticity,
    model.age.int.germ, test = T)$"p-value"[2]))
```

```
## [1] " p-value = 5.87418888791649e-158"
```

This comparison shows that a model allowing for within-individual heterogeneity results in an improved fit with a highly significant

p-value. Estimates for within individual standard deviation in mutation counts are 496 SBS mutations/crypt without polymerase mutations - WT, 3585 SBS mutations/crypt - POLE 424V, 1156 SBS mutations/crypt - POLD1S478N and 398 SBS mutations/crypt - POLD1 D316N and L474P.

```
c(WT = summary(model.age.int.germ)$sigma, (summary(model.age.int.germ)$sigma *
    coef(model.age.int.germ$modelStruct$varStruct, uncons = F)))
```

```
##         WT       POLE POLD1S478N POLD1other
##   495.6003  3584.5035  1155.5062   397.8756
```

4.Assessment of telomere attrition in wild-type and DNA polymerase mutant intestinal crypts

To assess whether telomere attrition is more accelerated in individuals carrying germline DNA polymerase exonuclease domain mutations, we analyse the telomere content of individual crypts (proxy for stem cells) in both normal wild-type crypts as well as those from this cohort. Comparison is made between the DNA polymerase cohort (considered as a whole) and normal controls (Lee-Six et al 2019, Nature) (see Methods for details of telomere length estimation).

Firstly we visualise this data and see a negative correlation between age and telomere content in normal crypts. This is likely attributable to the shortening of telomeres with each replicative cycle; increasing age exposes the intestinal stem cell to greater numbers of replicative cycles and hence greater telomere shortening. We do not observe any mutations activating genes associated with telomere length maintenance and make a conservative assumption that the telomere length maintenance mechanisms have similar levels of activity between samples across samples from different individuals and different germline states.

Using two mixed-effects models we assess model fit using a model incorporating genotype (POLE/D vs WT) and one without this categorical variable. This model suggests that model fit is better without the inclusion of the germline variable suggesting that the genotype is not a significant effect on telomere attrition.

```
joined <- read.delim("polymerase_normal_telomerehunter_length_for_model2.txt",
    header = T)
joined$patient = substr(joined$sample, 1, 7)

# genotype '1' = POLE/D and '0' = WT
plot(tel_content ~ age, data = joined[joined$genotype ==
    0, ], col = "grey", ylab = "Telomerehunter - content",
    xlim = c(0, 80), ylim = c(0, 900))
abline(lm(tel_content ~ age, data = joined[joined$genotype ==
    0, ]), col = "grey", lty = 2)
points(tel_content ~ age, data = joined[joined$genotype ==
    1, ], col = "dodgerblue")
abline(lm(tel_content ~ age, data = joined[joined$genotype ==
    1, ]), col = "dodgerblue", lty = 2)
legend("topleft", legend = c("normal", "POLE/D1"), col = c("grey",
    "dodgerblue"), box.lty = 0, cex = 0.8, pch = c(1, 1),
    bty = "n")
```

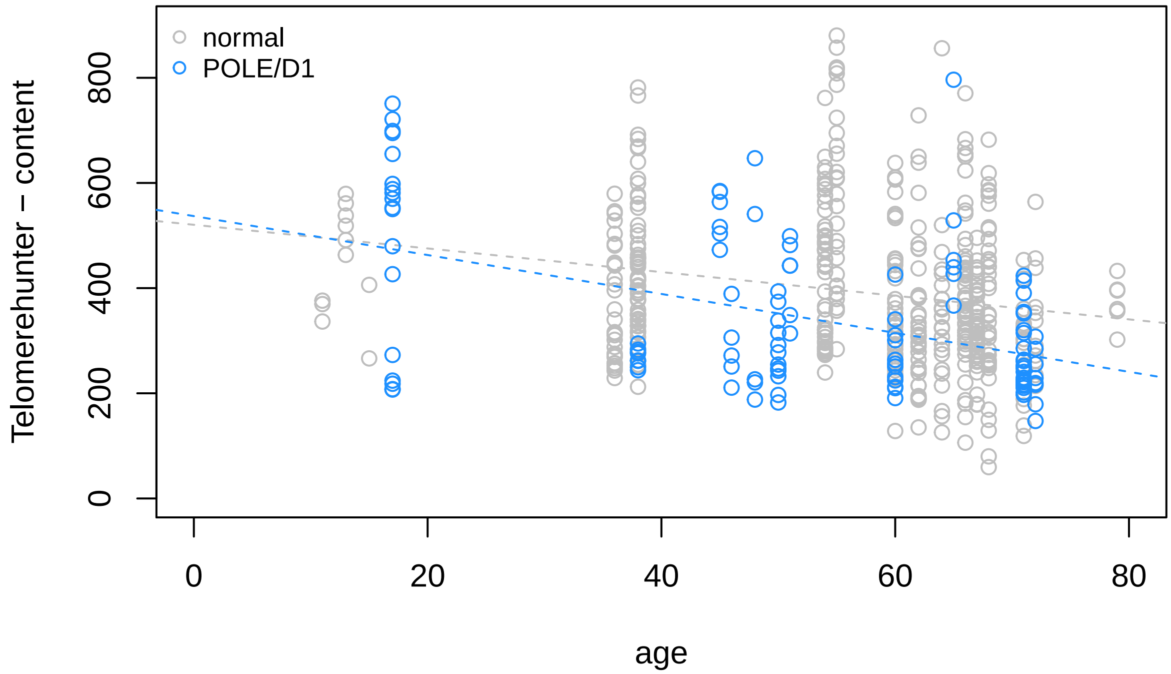


```r
# Model lmw - with genotype included as a nested effect
joined.lmw <- lme(fixed = tel_content ~ age + genotype,
    random = list(patient = pdSymm(form = ~genotype - 1)),
    weights = varIdent(form = ~1 | genotype), data = joined,
    method = "ML")


# Model lmw - with no inclusion of genotype
joined.lmwo <- lme(fixed = tel_content ~ age, random = list(patient = pdSymm(form = ~1)),
    weights = varIdent(form = ~1), data = joined, method = "ML")

summary(joined.lmwo)
```

```
## Linear mixed-effects model fit by maximum likelihood
##  Data: joined
##        AIC      BIC    logLik
##   6944.629 6961.898 -3468.314
##
## Random effects:
##  Formula: ~1 | patient
##         (Intercept) Residual
## StdDev:    78.42695 120.0295
##
## Fixed effects: tel_content ~ age
##                Value Std.Error  DF   t-value p-value
## (Intercept) 508.0369  48.78754 519 10.413250   0.000
## age          -2.6227   0.84504 519 -3.103616   0.002
##  Correlation:
##     (Intr)
## age -0.95
##
## Standardized Within-Group Residuals:
##        Min          Q1         Med          Q3         Max
```

```
## -2.4042717 -0.5982259 -0.1342493  0.5187133  3.6394728
##
## Number of Observations: 554
## Number of Groups: 34
```

```r
summary(joined.lmw)
```

```
## Linear mixed-effects model fit by maximum likelihood
##  Data: joined
##        AIC      BIC    logLik
##   7023.614 7049.517 -3505.807
##
## Random effects:
##  Formula: ~genotype - 1 | patient
##         genotype Residual
## StdDev: 86.23655  139.412
##
## Variance function:
##  Structure: Different standard deviations per stratum
##  Formula: ~1 | genotype
##  Parameter estimates:
##         0         1
## 1.0000000 0.7791055
## Fixed effects: tel_content ~ age + genotype
##                Value Std.Error  DF   t-value p-value
## (Intercept) 524.3607 27.741931 519 18.901376   0.000
## age          -2.3171  0.472966 519 -4.899075   0.000
## genotype    -52.1444 27.283302  32 -1.911222   0.065
##  Correlation:
##          (Intr) age
## age      -0.971
## genotype -0.108  0.052
##
## Standardized Within-Group Residuals:
##        Min          Q1         Med          Q3         Max
## -2.6455257 -0.6311778 -0.1437166  0.5180956  3.4675608
##
## Number of Observations: 554
## Number of Groups: 34
```

```r
intervals(joined.lmw, which = "fixed")
```

```
## Approximate 95% confidence intervals
##
##  Fixed effects:
##                   lower       est.      upper
## (Intercept)  470.008150 524.360667 578.713185
## age           -3.243737  -2.317094  -1.390451
## genotype    -107.568028 -52.144436   3.279156
## attr(,"label")
## [1] "Fixed effects:"
```

```r
intervals(joined.lmwo, which = "fixed")
```

```
## Approximate 95% confidence intervals
##
```

```
##  Fixed effects:
##                  lower       est.      upper
## (Intercept) 412.364686 508.036858 603.709029
## age          -4.279798  -2.622678  -0.965559
## attr(,"label")
## [1] "Fixed effects:"
```

```
anova(joined.lmwo, joined.lmw, test = T)
```

```
##             Model df      AIC      BIC    logLik   Test  L.Ratio p-value
## joined.lmwo     1  4 6944.629 6961.898 -3468.314
## joined.lmw      2  6 7023.614 7049.517 -3505.807 1 vs 2 74.98465  <.0001
```

Supplementary methods

Mutational signature extraction

Fourteen signature components were extracted; HDP0-HDP13 (Supplementary Information Fig. 1). To deconvolute composite signatures and to equate obtained HDP signatures to reference ones, we assessed the cosine similarity and employed an EM-algorithm to deconstruct these signatures into reference constituents (SBS1, SBS5, SBS7a, SBS7b, SBS10a, SBS10b, SBS17a, SBS17b, SBS25, SBS28, SBS31, SBS35, SBS88 and SBS89[1,2]). Deconstructed signature components are displayed in Supplementary Information Fig. 2. In this way, HDP3 was broken down into SBS1 and SBS5; HDP4 into SBS10a and SBS5; HDP5 into SBS1, SBS5, SBS10a, and SBS28; HDP9 into SBS7a, SBS7b, SBS10a, and SBS28. HDP1 was broken down into SBS10a and SBS10b, but SBS10b poorly reflected the observed C>T component of HDP1 and HDP6, which heavily impacted later fitting of signatures. Hence, we constructed our version of SBS10b by subtracting the estimated contribution of SBS10a from HDP1. In this vein, HDP1 became SBS10a and SBS10b, and HDP6 was decomposed into SBS10b, SBS1, SBS5, and SBS28.

HDP2 and 7, exclusively found in patients with a POLD1 germline mutation, were renamed as SBS10c and SBS10d, respectively, and not subjected to decomposition. HDP13 found in multiple samples from an individual with a POLE germline mutation was renamed SBS91. HDP0, 8 and 11 only had one major contributor and were replaced by the purer reference signatures SBS5, SBS88, and SBS89. HDP10 was found to be due to platinum-based chemotherapy, as it is constituted of SBS31 and SBS35. However, the report of a spectrum of signatures due to these chemotherapy agents[3] (Extended Figure 1b) and the relatively poor reconstitution using SBS31 and SBS35 only, prompted us to retain HDP10 without further decomposition hence, we name it SBS35-like. A similar approach was used for the capecitabine-related HDP12 component, which resembles SBS17b but has closer similarity to previously reported therapy-related signatures[3,4]. Hence, we renamed HDP12; SBS17b-like.

Therefore, we identify a total of 14 signatures: SBS1, SBS5, SBS7ab, SBS10a-d, SBS17b-like, SBS28, SBS35-like, SBS88, SBS89 and SBS91. These signatures were refitted to all mutation counts of branches of phylogenies using the R package sigfit (https://github.com/kgori/sigfit)[5]. To avoid overfitting, a limited subset of reference mutational signatures were included per patient corresponding to the HDP signatures that have been identified in that individual. In the case of SBS10d, it was only fitted to branches in which an exposure had originally been reported.

Validation of mutational signatures

To verify the mutational signatures extracted using HDP, we used the non-negative matrix factorisation (NMF) based algorithm SigProfiler[1] to extract SBS mutational signatures. SigProfiler reports fewer substitution signature components (7 vs 14) but the components it does extract have clearly recognisable counterparts in the compendium of HDP components (Supplementary Information Fig. 3 and Fig. 4). Additional components that are stably extracted and have close resemblance to known, reported signatures were identified by HDP but not SigProfiler.

HDP combined mutational signature extraction

To assess the association between signatures in different mutational classes and to validate the findings of previous iterations of signature extraction, combined extraction of SBS, ID and doublet based substitutions (DBS) was performed using *de novo* extraction with HDP. This leading to a direct incorporation and simultaneous extraction of SBS, ID and DBS components. Count matrices were constructed from unique mutations. For computational efficiency mutations were subsampled to a maximum of 3000 per sample. As previously, low mutation burden samples; those with fewer than 100 mutations, were excluded.

The following parameters were used:
Chains: 20 MCMC chains

Iterations: 40,000
Burn-in: 20,000
Samples: 200 / chain
Signature components identified: 11
Component Names: HDP0-HDP10

Extracted components are displayed in Supplementary Information Fig. 5. These results confirm known associations of mutational signatures across different mutation classes e.g. ubiquitous signatures: SBS1 & SBS5 & ID1, platinum therapy exposure: SBS35 & DBS5 (component 7), colibactin exposure: SBS88 & ID18 (component 5) and UV light: SBS7a & DBS1 (component 8). Furthermore they demonstrate an association between activities of polymerase signatures i.e. defective POLE proofreading: SBS10a / SBS10b & ID1 (component 1 and 4) and SBS10c & ID1 (component 2).

Validation and extension of mutational signature analysis

Of the novel mutational signatures identified, two bear similarity to published mutational signatures; SBS10c is similar to SBS56 and SBS91 to SBS52. SBS52 and SBS56 are found sporadically in a small number of cancer types and are thought to be possible artefacts[6]. The following observations are made and analyses undertaken to evaluate the evidence for the validity of SBS10c, SBS10d and SBS91.

Firstly, we manually inspected and verified a random sample of somatic mutations from each mutational signature using the JBrowse genome browser (SBS10c; 200 mutations, SBS10d; 100 mutations and SBS91; 500 mutations). Representative mutations associated with each mutational signature (SBS10c, SBS10d and SBS91) are shown in Extended Data Fig. 5, 6 and Supplementary Information Fig. 6.

1. Validation of mutations and mutational signatures SBS10c and SBS10d
    - Novel mutational signatures were identified in the following number of independently processed samples; SBS10c was identified in 52 samples and SBS10d was identified in 3 samples.
    - Using signature assignment (outlined below), mutations were attributed to each mutational signature; ~170000 mutations to SBS10c and ~220000 mutations to SBS10d.
    - SBS10c and SBS10d have not been identified in other human tissue samples processed with the same laboratory pipeline indicating that these are not systematic artefacts.
    - SBS10c and SBS10d mutational signatures have not been identified in whole-genome sequenced samples in other cohorts of normal tissues sequenced with the same Illumina Novaseq platform suggesting that they are not sequencing artefacts
    - Individual mutations assigned to each signature were independently identified in multiple samples (Extended Data Fig. 5 and 6). That means that the mutations have been independently isolated, sequenced and hence validated and that they, therefore, cannot have occurred due to a laboratory or sequencing artefact.
2. Aetiology of SBS10c and SBS10d
    - SBS10c and SBS10d mutational signatures were only identified in individuals carrying POLD1 mutations and not in individuals with POLE mutations.
    - The replication strand bias, replication timing, extended sequence context of signatures SBS10c, SBS10d were characterised and summarised (Extended Data Fig. 5 and 6). SBS10c and SBS10d show strong replication strand bias, with an excess of mutations on the lagging strand consistent with the role of POLD1 and consistent with previous characterisation of POLD1 in experimental systems. In addition to the strong trinucleotide sequence context bias, they also demonstrate strong extended sequence context bias, consistent with the polymerase signatures that are seen in POLE signatures SBS10a and SBS10b. These characteristics along with the validation of the SBS10c-d signature across multiple individuals with POLD1 mutations and multiple samples from each individual provide support

that the signatures are *bona fide* and the aetiology is the result of the defective DNA proofreading due to the inherited mutation in POLD1.

3. Validation of SBS91
    - SBS91 was identified in 6 independently isolated and sequenced tissue samples
    - 907 mutations were confidently assigned to this mutational signature
    - This signature was found in samples from one individual in this cohort. It was not identified in other individuals in this cohort, suggesting that it is unlikely to be the result of a systematic artefact
    - Furthermore, SBS91 has not been identified in other human tissue samples processed with the same laboratory pipeline and sequencing platform indicating that these are not obviously systematic or sequencing artefacts.
    - Shared mutations were observed across multiple samples taken from the sample tissue section. Some mutations were shared across samples, others were not.
    - The SBS91 mutation burden was highest in three adjacent samples (Supplementary Information Fig. 6) and lower in more distant samples indicating the presence of a clone of cells giving rise to the mutations. Such spatial clustering / arrangement would not be expected with an artefact.
    - The genome distribution of SBS91 mutations showed a strong bias toward early replicating and exonic regions in contrast with SBS10a,b,c,d which show a depletion in exons and in the case of SBS10a late replication timing bias.
    - The extended sequence context of SBS91 demonstrates a distinctive motif GGGTGGTCT<u>C</u> (mutated base underlined) and is enriched in short interspersed repeats of the Alu Family (Supplementary Information Fig. 6). Sequencing artefacts do not typically exhibit such specific traits.
    - We attempted to phase somatic SBS91 mutations to nearby heterozygous single nucleotide polymorphisms (snps). We found multiple examples of mutations in close proximity to known heterozygous snps. Artefactual mutations would be expected to be agnostic to the phasing of snps hence would occur randomly and not obey phase. We observe both mutations that obey the phasing of mutations and also those that do not.
4. Aetiology of SBS91
    - In summary, SBS91 was identified in multiple samples from an individual across at least 2 tissue types.
    - The signature demonstrates some features, outlined above which suggest it is behaving in a manner that is not typical of conventional SBS signatures.
    - Since SBS91 behaves in an atypical manner we have less confidence in attributing it to the defective DNA polymerases, it may indeed arise due to a different aetiology.
4. Summary for SBS91
    - There are many features compatible with SBS91 being a genuine biological signature.
    - A degree of non-phasing with local SNPs is, however, consistent with it being artefact. Nevertheless, the strong and distinctive local sequence context may mean that SBS91, if biological, hypermutates certain sequences and this could also potentially explain non-phasing with local SNPs.
    - The targeting to Alu sequences is dissimilar from other Polymerase deficiency signatures and thus, even if biological, its origin is unclear.
    - Because of multiple uncertainties concerning this signature we have not discussed it in the main text and have not discussed samples that carry it.
    - However, further details are in Supplementary Information Fig. 6.

Mutational signature assignment

To assess the likely causative mutational signatures for individual mutations, all SBS mutations that mapped to branches of the phylogenetic trees were assigned signature probabilities. These assigned probabilities were used to subset mutations for further analysis i.e. replication strand and extended sequence context biases as displayed in Extended Data Fig. 7.

Mutational signature assignment was performed for all SBS mutations assigned to phylogenetic trees treating each branch / edge as a unique sample, hence ensuring that mutations were not double counted. HDP mutational signature extraction with deconvolution into reference signatures was undertaken to define the proportion of each mutational signature in each sample. Deconvoluted signatures were used to define the relative probability of each trinucleotide context per signature. Mutational assignment probability was defined as the probability P that, that a particular mutation, i, could be assigned to a given signature, j, in genome k was calculated as follows:

$$P_{i,j,k} \frac{w_{j,k} \cdot f_{i,j}}{\sum_i w_{j,k} \cdot f_{i,j}}$$

where $w_{j,k}$ is the proportion of mutations assigned to signature j in genome k and $f_{i,j}$ is the fraction of mutations in signature j that are the same substitution type and occur at the same trinucleotide context as mutation i.

References

1 Alexandrov, L. B. *et al.* The repertoire of mutational signatures in human cancer. *Nature* **578**, 94-101, doi:10.1038/s41586-020-1943-3 (2020).

2 Lee-Six, H. *et al.* The landscape of somatic mutation in normal colorectal epithelial cells. *Nature* **574**, 532-537, doi:10.1038/s41586-019-1672-7 (2019).

3 Pich, O. *et al.* The mutational footprints of cancer therapies. *Nature Genetics* **51**, 1732-+, doi:10.1038/s41588-019-0525-5 (2019).

4 Christensen, S. *et al.* 5-Fluorouracil treatment induces characteristic T>G mutations in human cancer. *Nature Communications* **10**, 4571, doi:10.1038/s41467-019-12594-8 (2019).

5 Gori, K. & Baez-Ortega, A. sigfit: flexible Bayesian inference of mutational signatures. *bioRxiv* (2020).

6 Sondka, Z. *et al.* The COSMIC Cancer Gene Census: describing genetic dysfunction across all human cancers. *Nat Rev Cancer* **18**, 696-705, doi:10.1038/s41568-018-0060-1 (2018).

Supplementary Information Figure 1| HDP signature components

Fourteen signature components were extracted; HDP0-HDP13. Plots show mutational spectra of extracted components. Trinucleotide context (x-axis) and mean proportion (y-axis). Credibility intervals (95%) are displayed as a thin black line above and below each bar. Trinucleotide contexts which are not statistically significant are shown in light grey. A subset of these 14 signatures appeared to be combinations of previously reported reference signatures[1].

Component 0

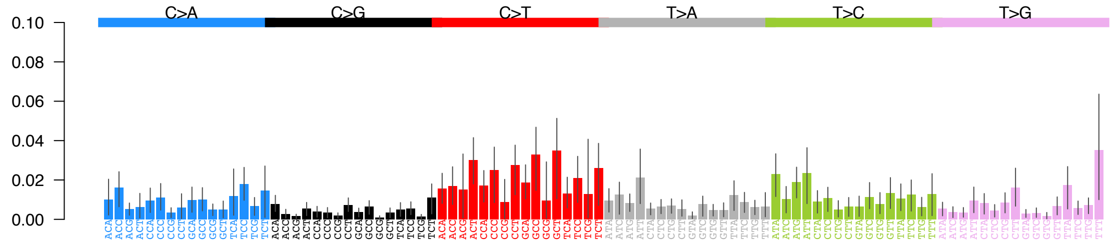

Component 1

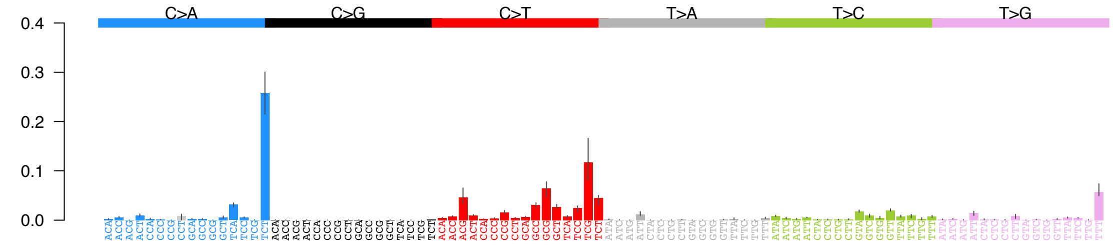

Component 2

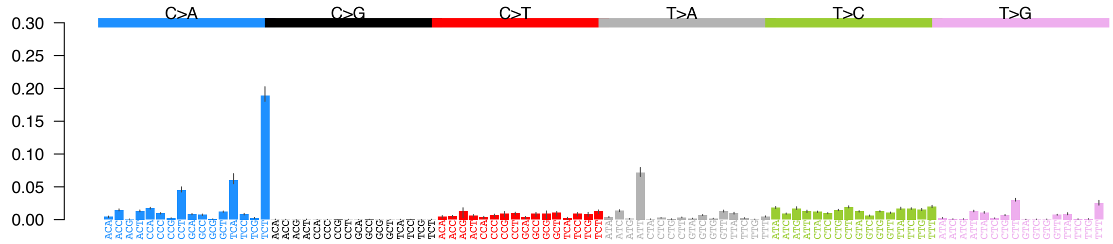

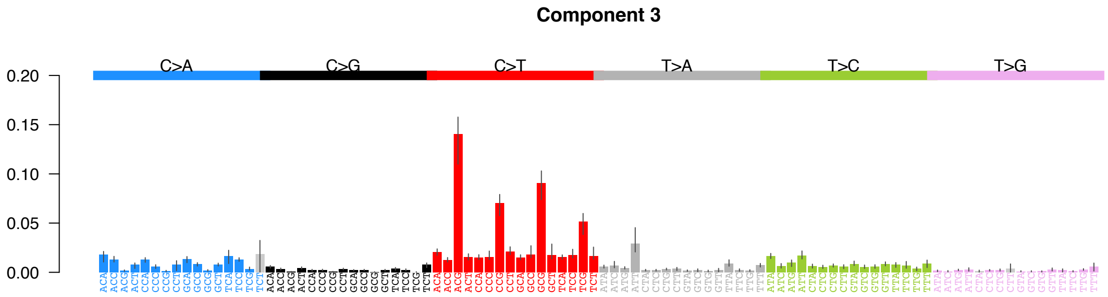
Component 3
C>A
C>G
C>T
T>A
T>C
T>G
0.20
0.15
0.10
0.05
0.00

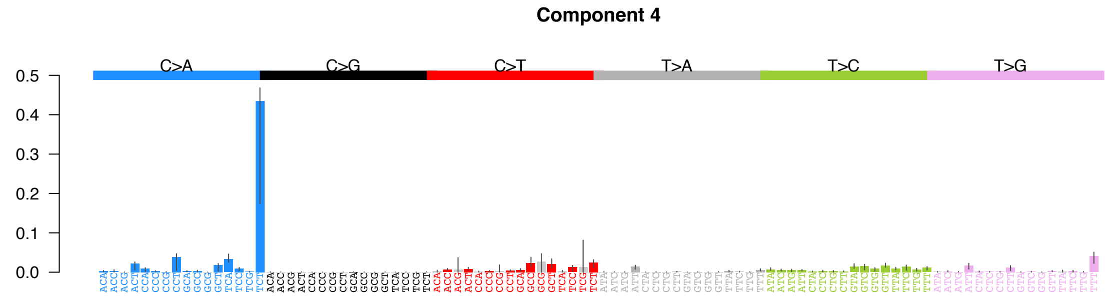
Component 4
C>A
C>G
C>T
T>A
T>C
T>G
0.5
0.4
0.3
0.2
0.1
0.0

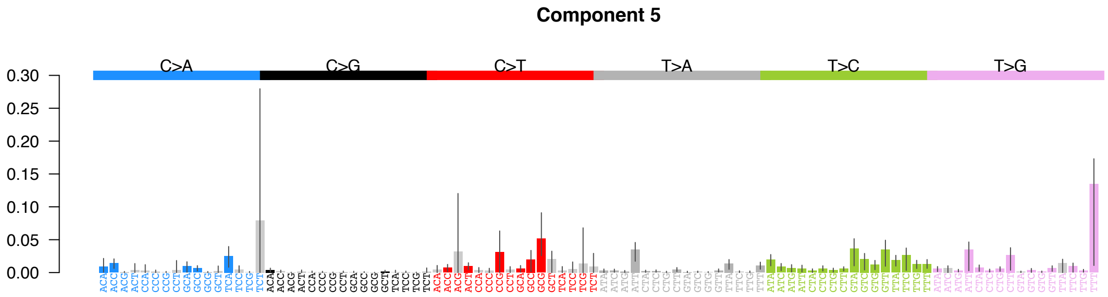
Component 5
C>A
C>G
C>T
T>A
T>C
T>G
0.30
0.25
0.20
0.15
0.10
0.05
0.00

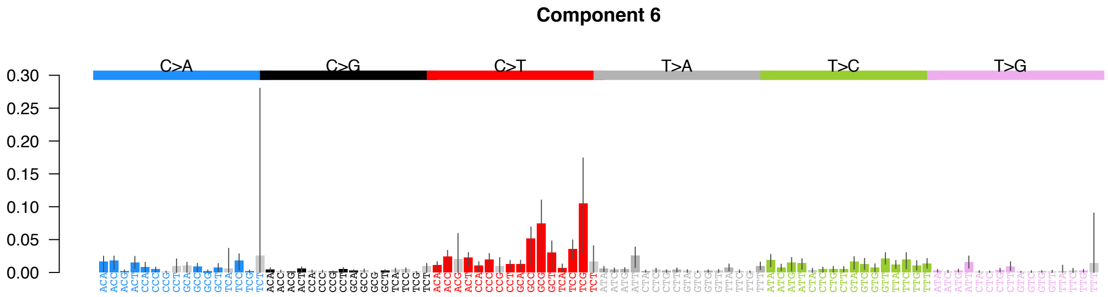
Component 6
C>A
C>G
C>T
T>A
T>C
T>G
0.30
0.25
0.20
0.15
0.10
0.05
0.00

Component 7

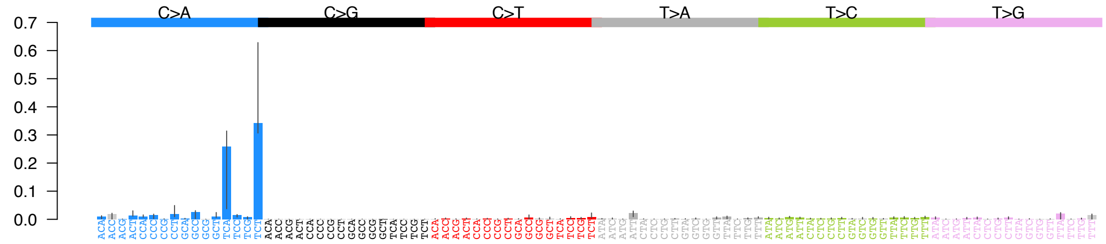

Component 8

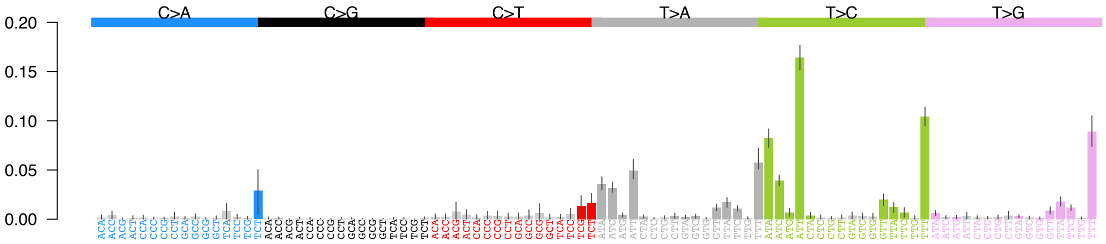

Component 9

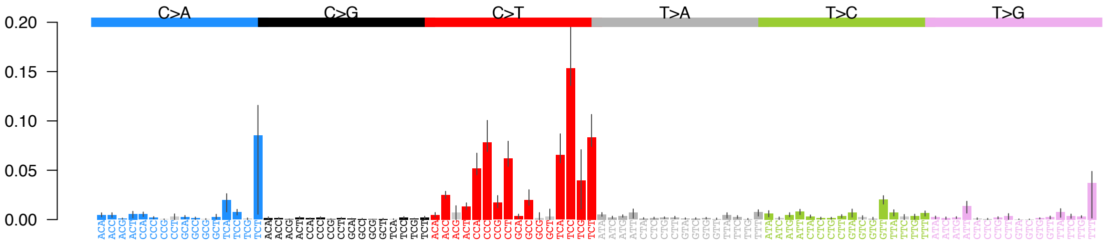

Component 10

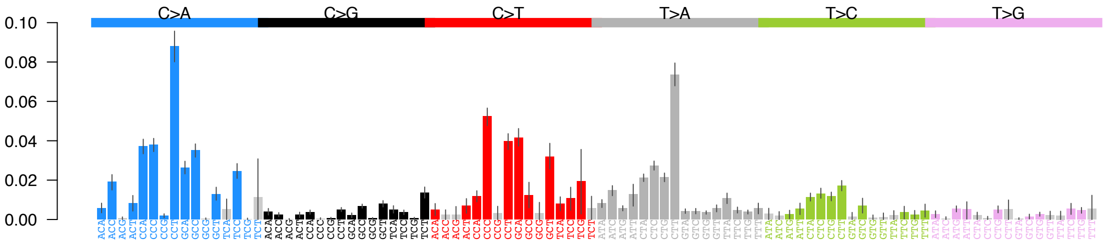

Component 11

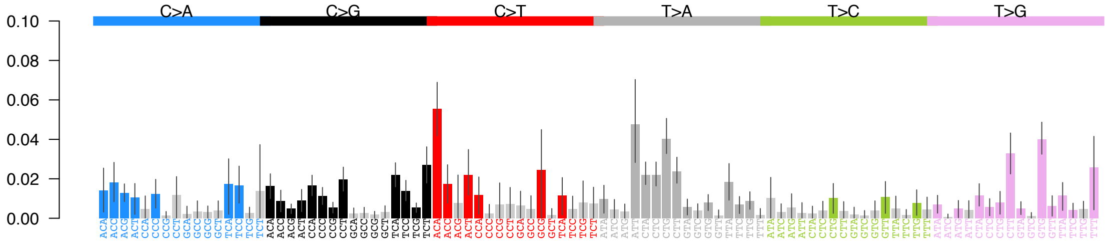

Component 12

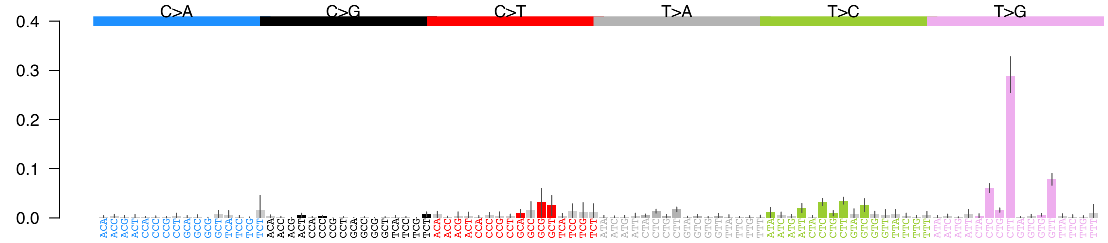

Component 13

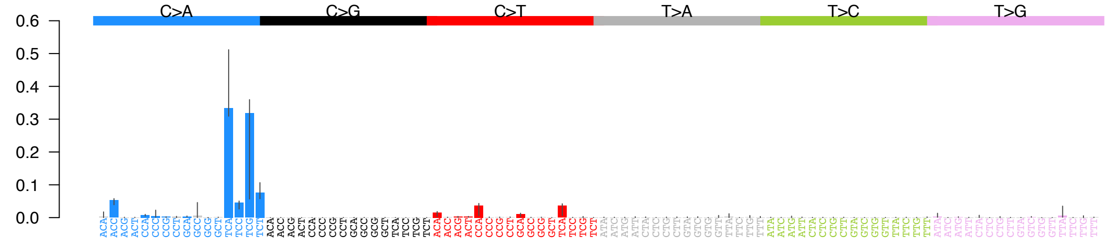

Supplementary Information Figure 2| HDP signature component deconvolution

Plots show mutational spectra of extracted component; trinucleotide context (x-axis) and mean proportion (y-axis). Each HDP component is displayed in the uppermost panel followed by the reconstituted component after deconvolution and its cosine similarity to the original HDP component. COSMIC reference signatures and their relative contributions are displayed on the lower panels. Relative contribution is displayed in the title of the plot.

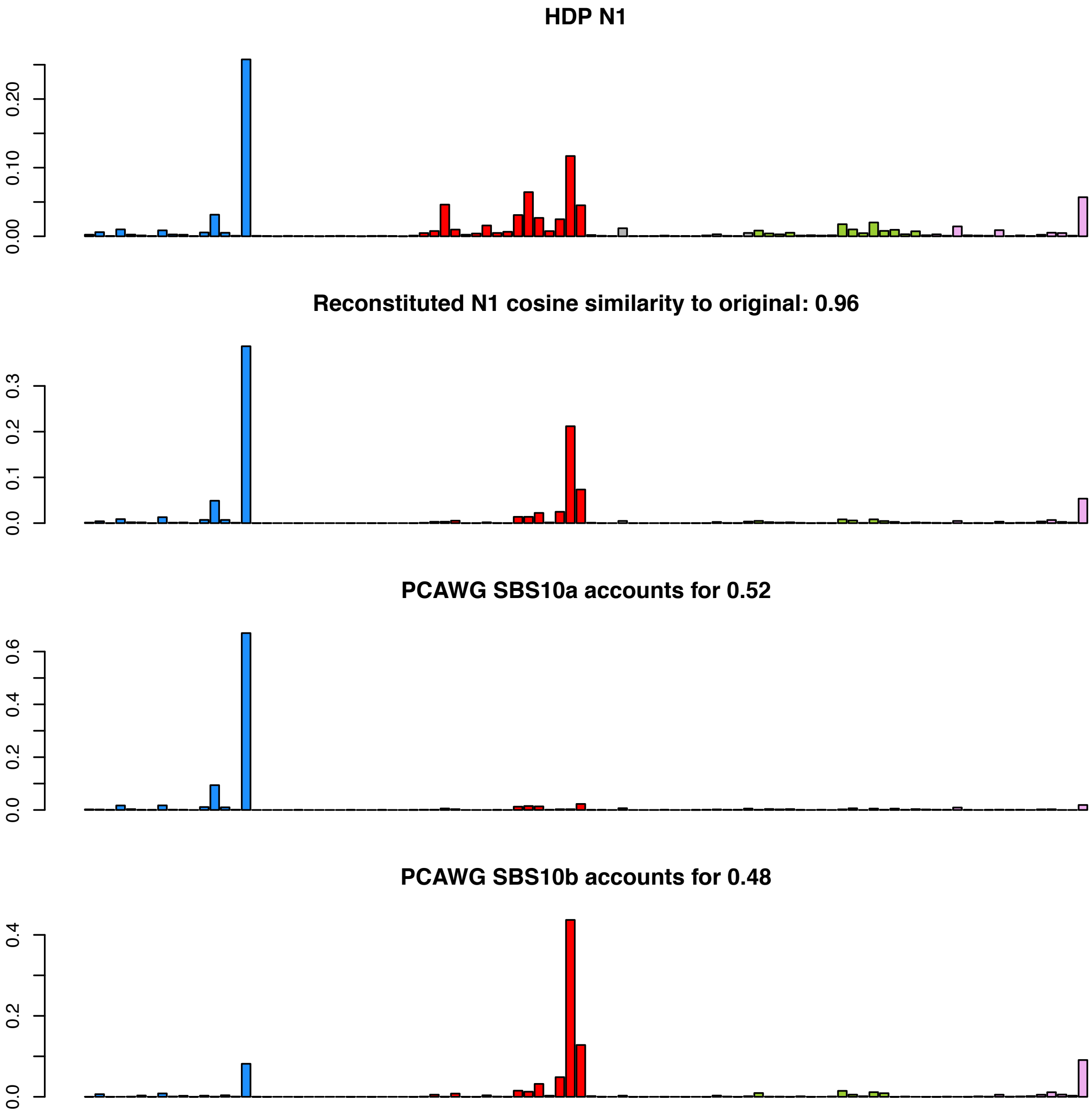
HDP N1
0.00
0.10
0.20
Reconstituted N1 cosine similarity to original: 0.96
0.0
0.1
0.2
0.3
PCAWG SBS10a accounts for 0.52
0.0
0.2
0.4
0.6
PCAWG SBS10b accounts for 0.48
0.0
0.2
0.4

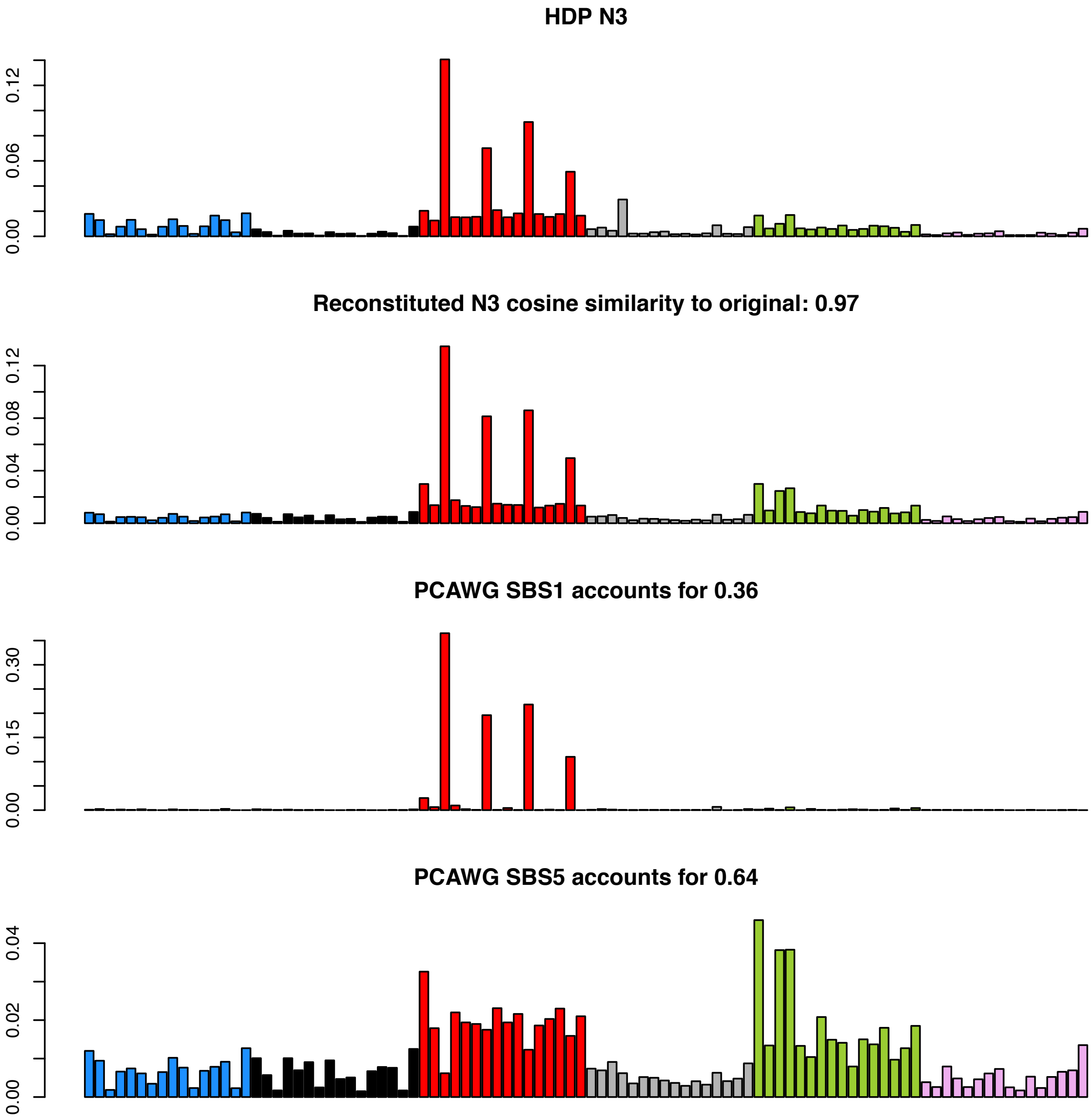
HDP N3
0.00
0.06
0.12
Reconstituted N3 cosine similarity to original: 0.97
0.00
0.04
0.08
0.12
PCAWG SBS1 accounts for 0.36
0.00
0.15
0.30
PCAWG SBS5 accounts for 0.64
0.00
0.02
0.04

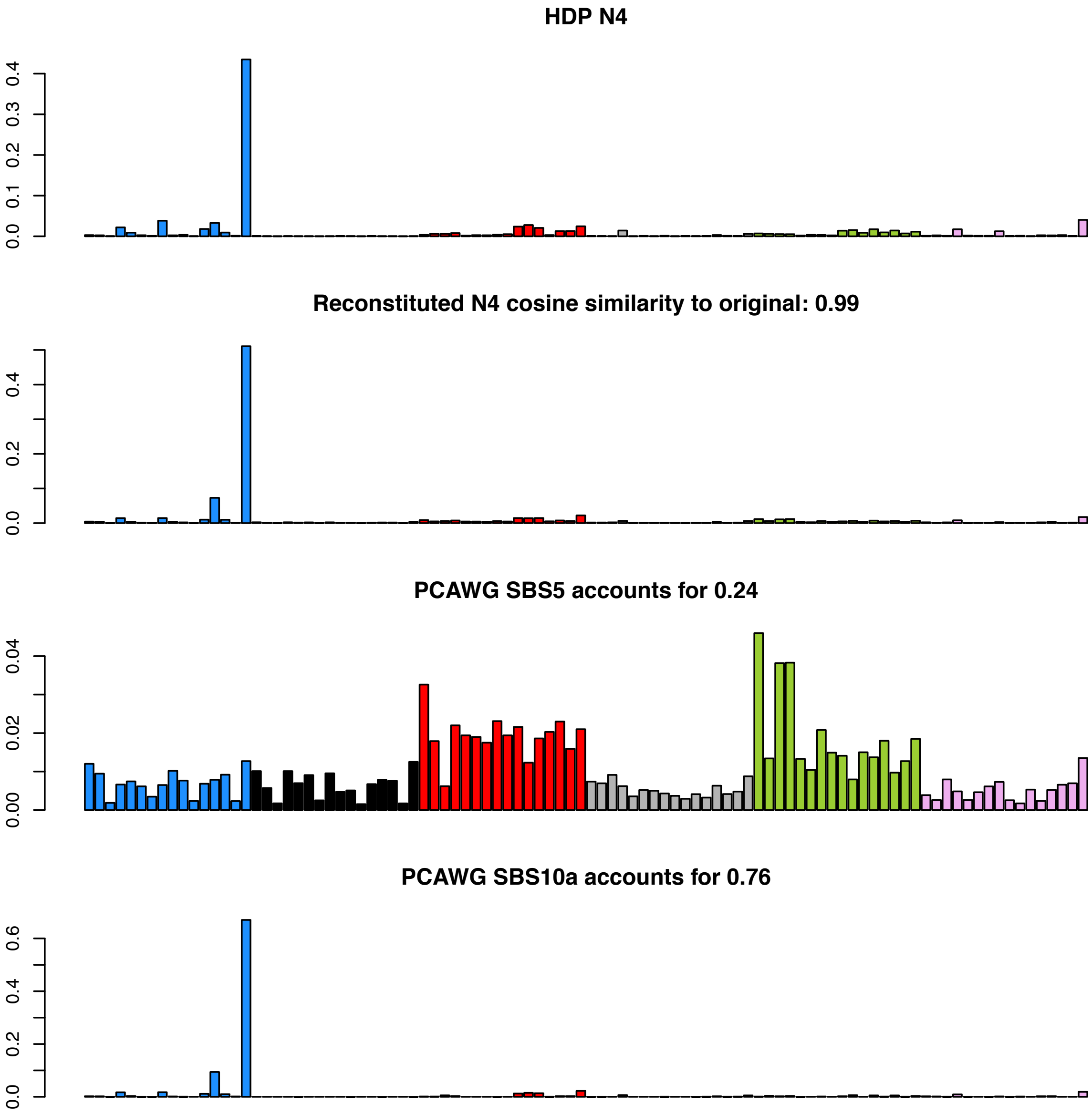
HDP N4
Reconstituted N4 cosine similarity to original: 0.99
PCAWG SBS5 accounts for 0.24
PCAWG SBS10a accounts for 0.76

HDP N5

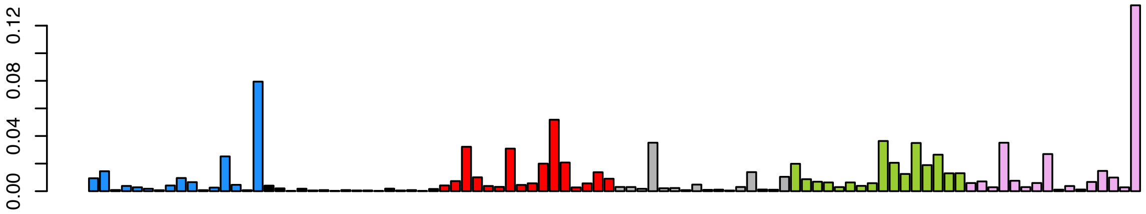

Reconstituted N5 cosine similarity to original: 0.81

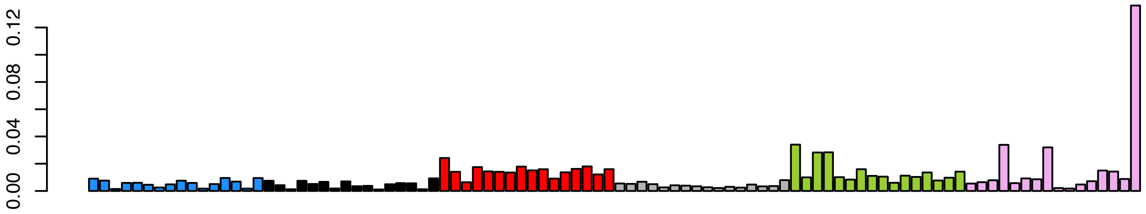

PCAWG SBS5 accounts for 0.74

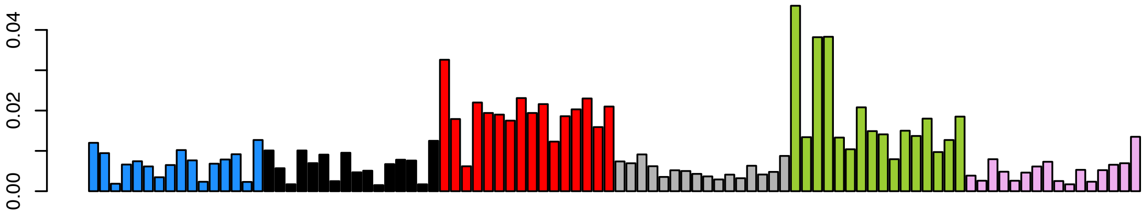

PCAWG SBS28 accounts for 0.26

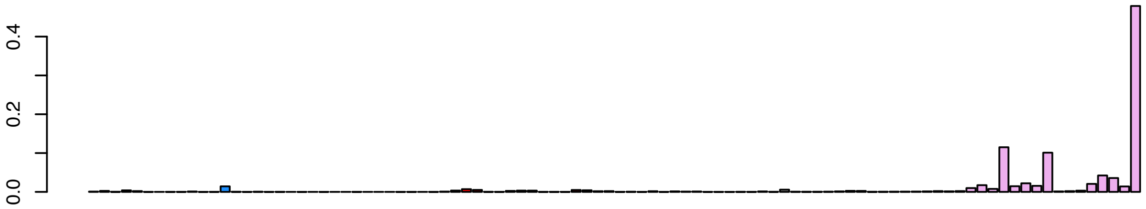

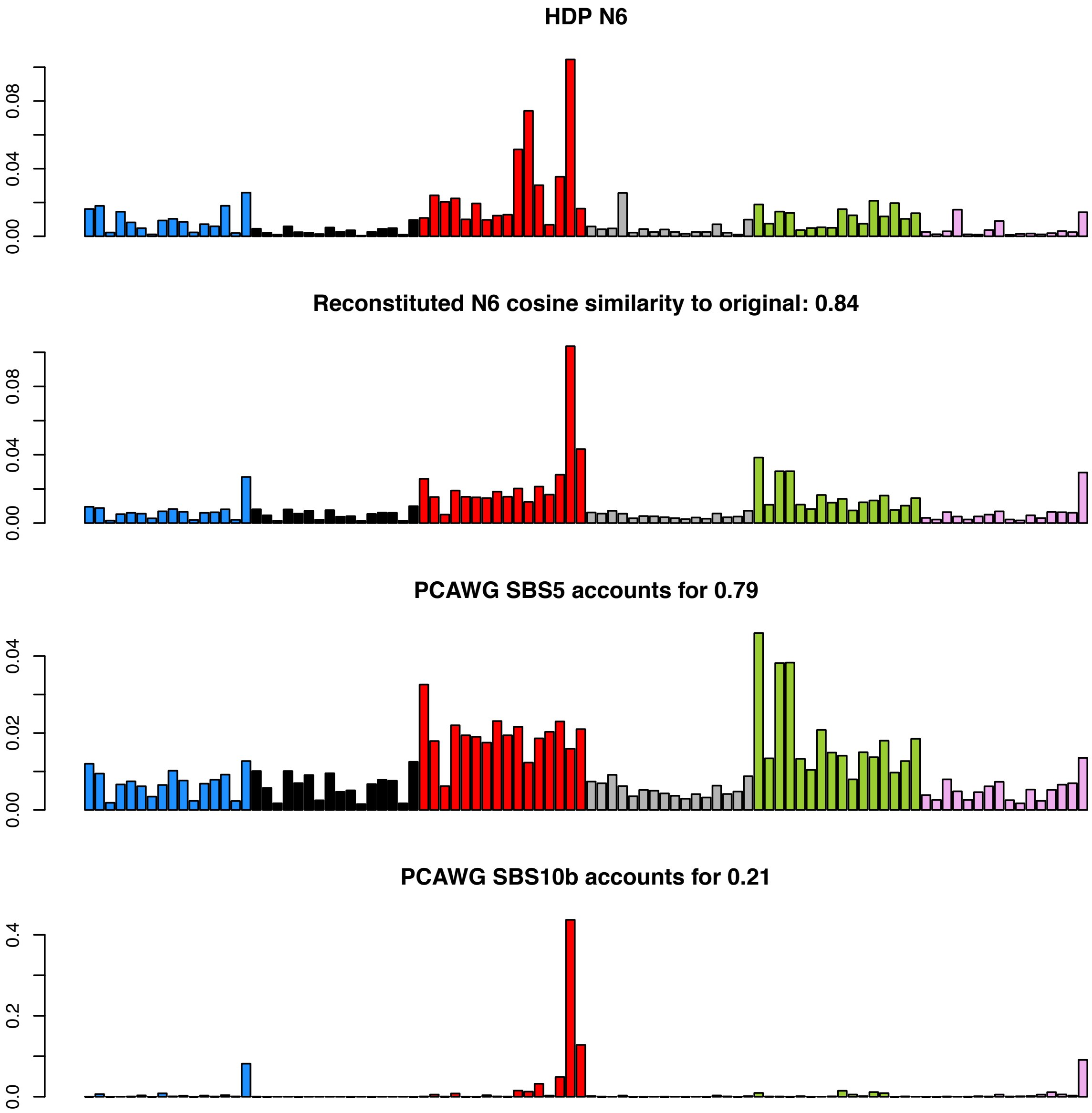
HDP N6
0.00
0.04
0.08
Reconstituted N6 cosine similarity to original: 0.84
0.00
0.04
0.08
PCAWG SBS5 accounts for 0.79
0.00
0.02
0.04
PCAWG SBS10b accounts for 0.21
0.0
0.2
0.4

HDP N9

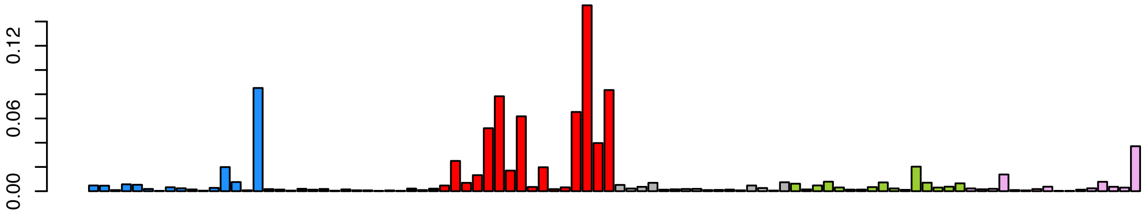

Reconstituted N9 cosine similarity to original: 0.89

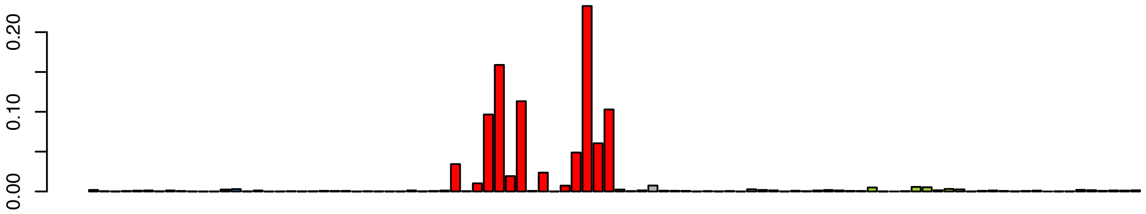

PCAWG SBS7a accounts for 0.18

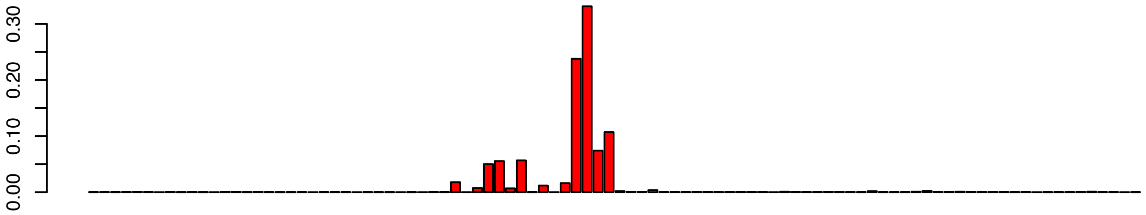

PCAWG SBS7b accounts for 0.82

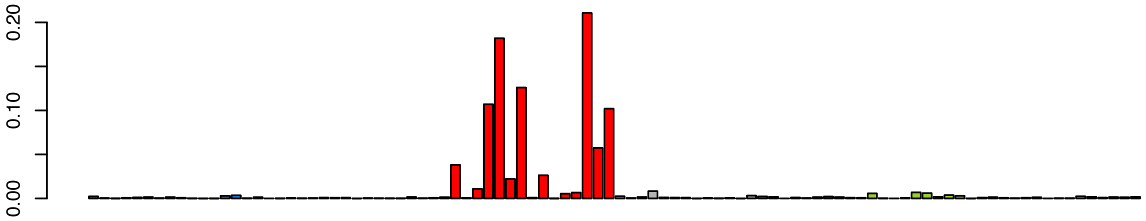

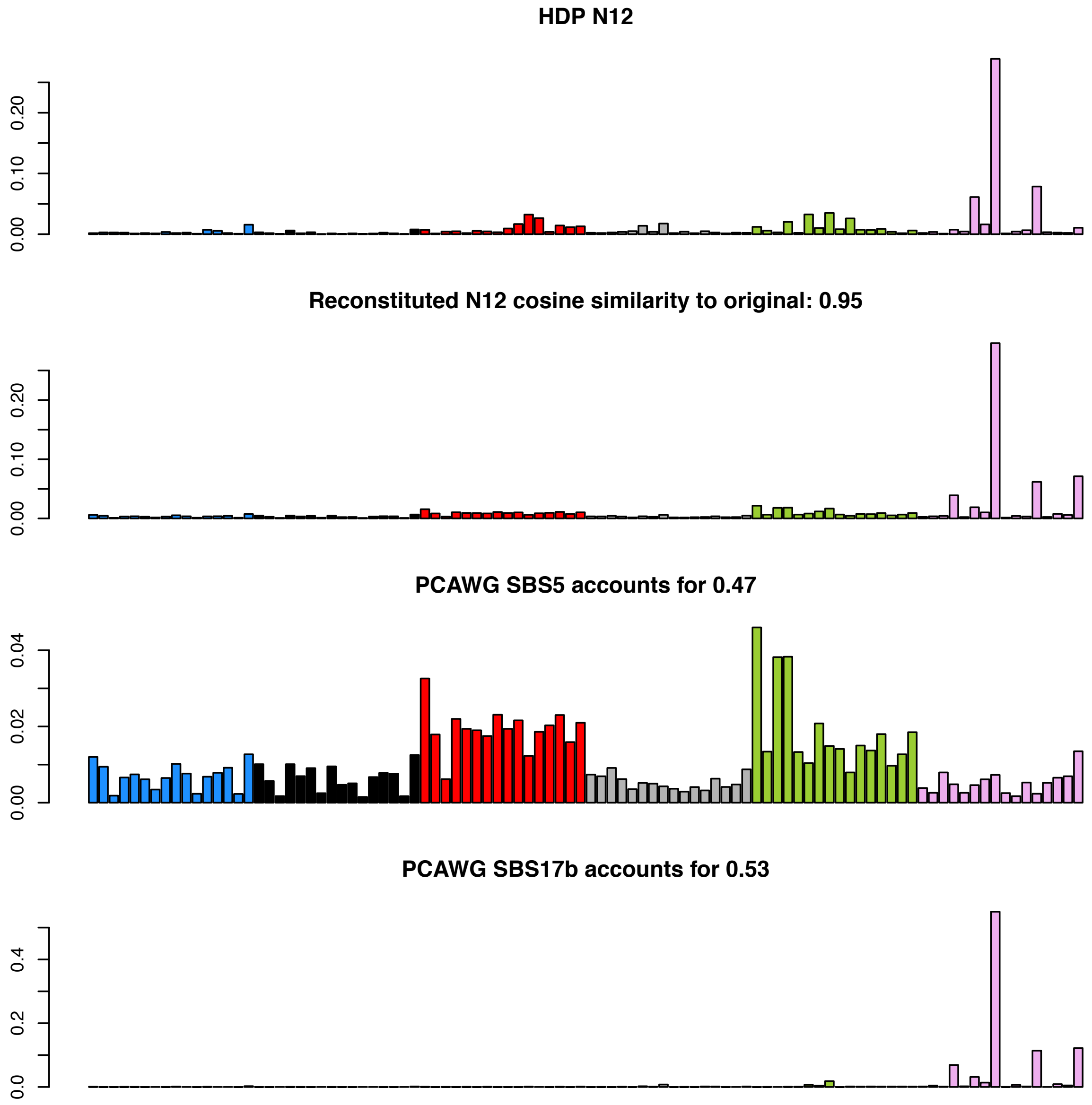
HDP N12
0.00
0.10
0.20
Reconstituted N12 cosine similarity to original: 0.95
0.00
0.10
0.20
PCAWG SBS5 accounts for 0.47
0.00
0.02
0.04
PCAWG SBS17b accounts for 0.53
0.0
0.2
0.4

Supplementary Information Figure 3 | Comparison of HDP and SigProfiler signature components

Comparison of signature extraction with HDP and SigProfiler was performed (methods). No new signatures were identified using SigProfiler and more mixing between signature components was observed using Sigprofiler compared with HDP.

Plots show mutational spectra of extracted components showing trinucleotide context (x-axis) and mean proportion (y-axis). Credibility intervals (95%) are displayed as a thin black line above and below each bar. Trinucleotide contexts whose contribution is not statistically significant are shown as light grey bars.

HDP components

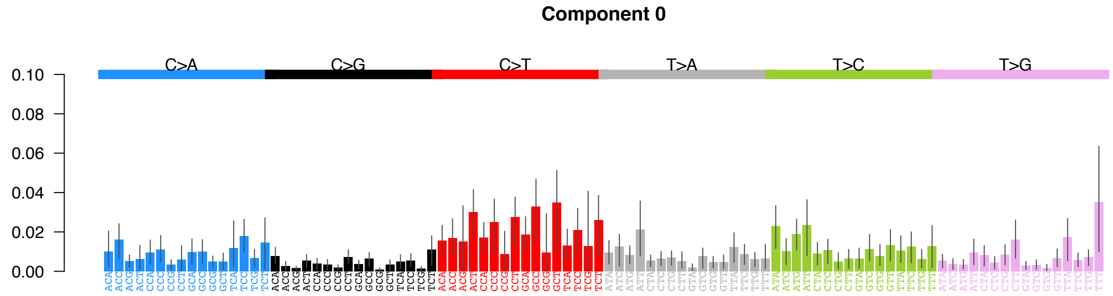

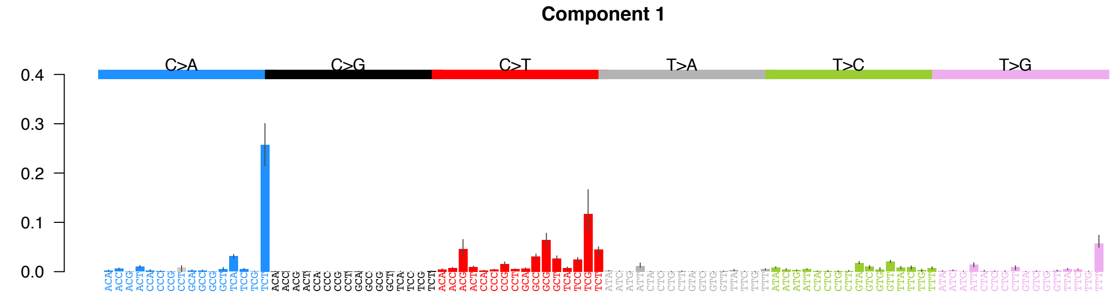

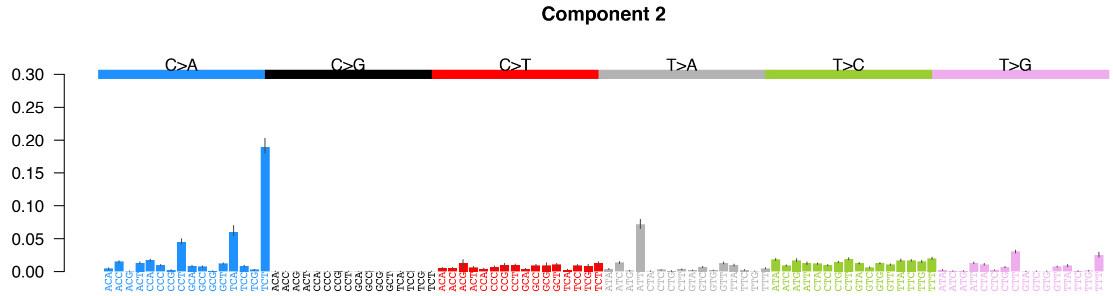

Component 3

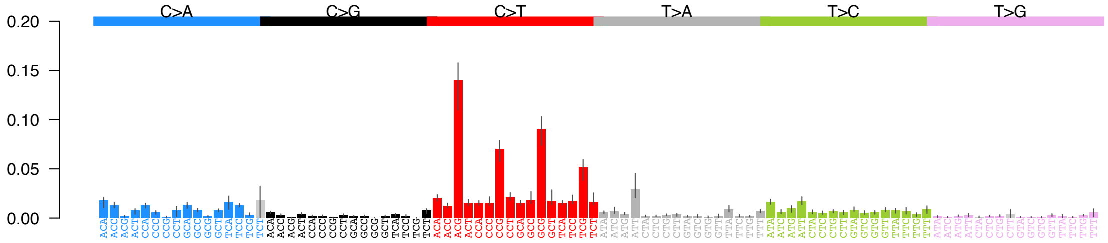

Component 4

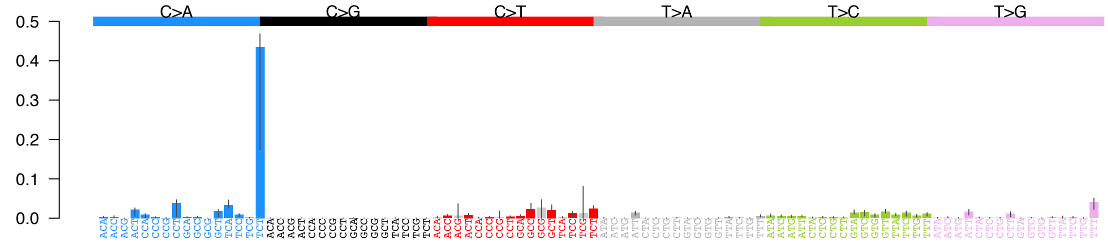

Component 5

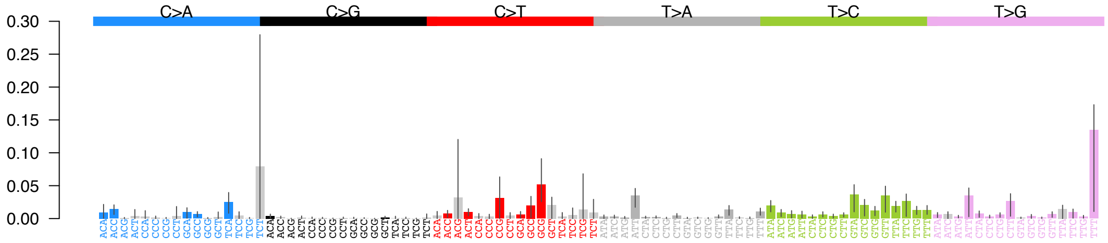

Component 6

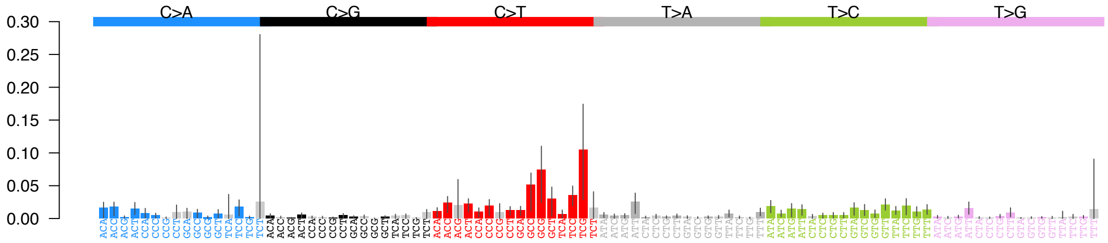

Component 7

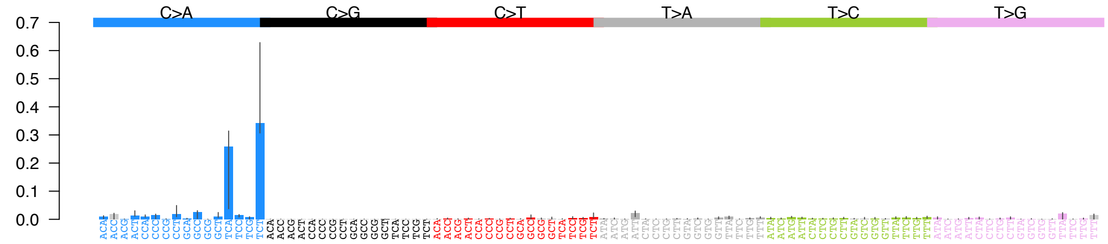

Component 8

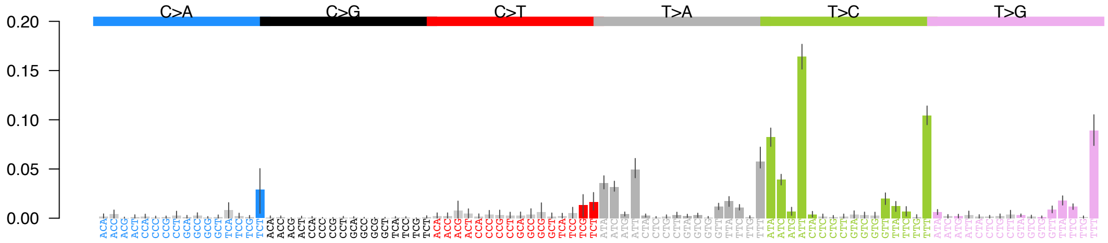

Component 9

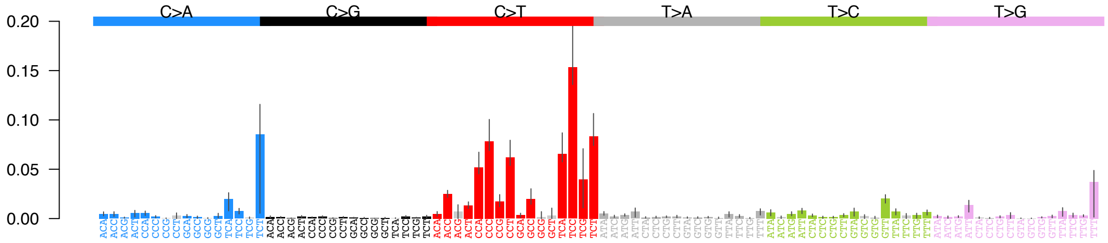

Component 10

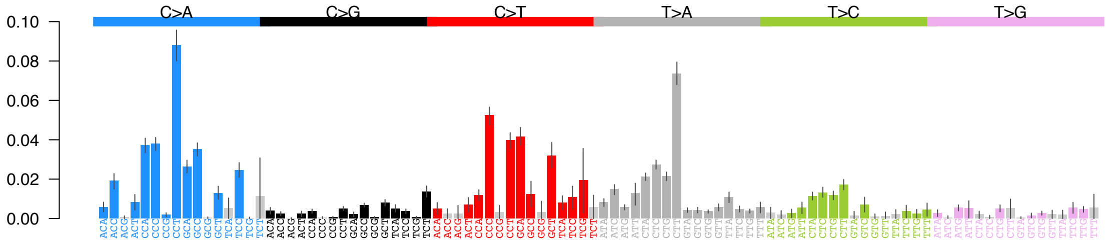

Component 11

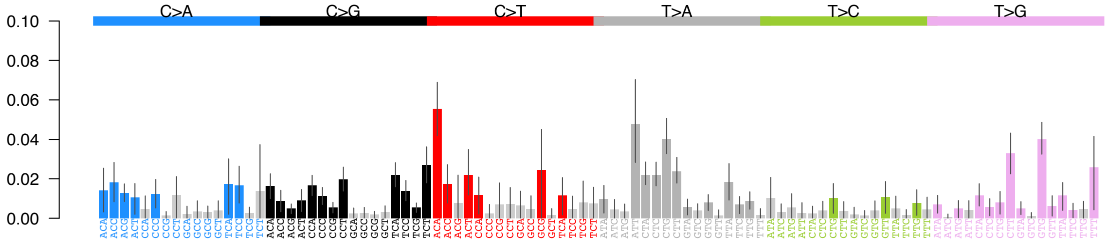

Component 12

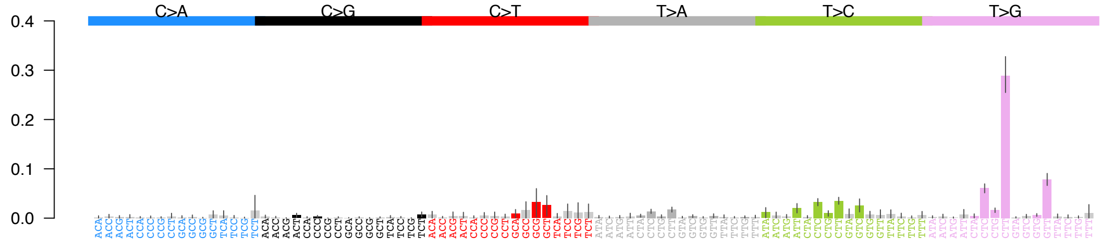

Component 13

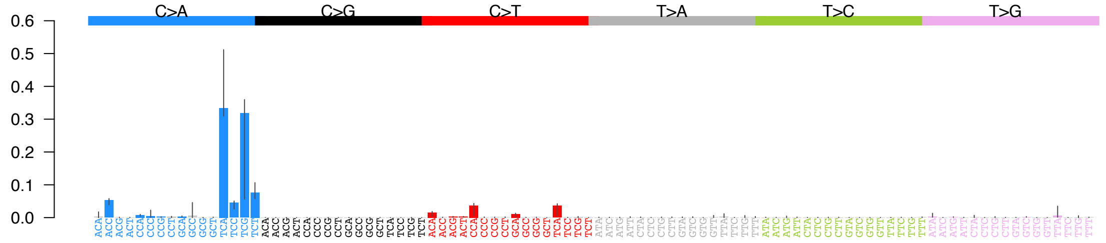

SigProfiler Components

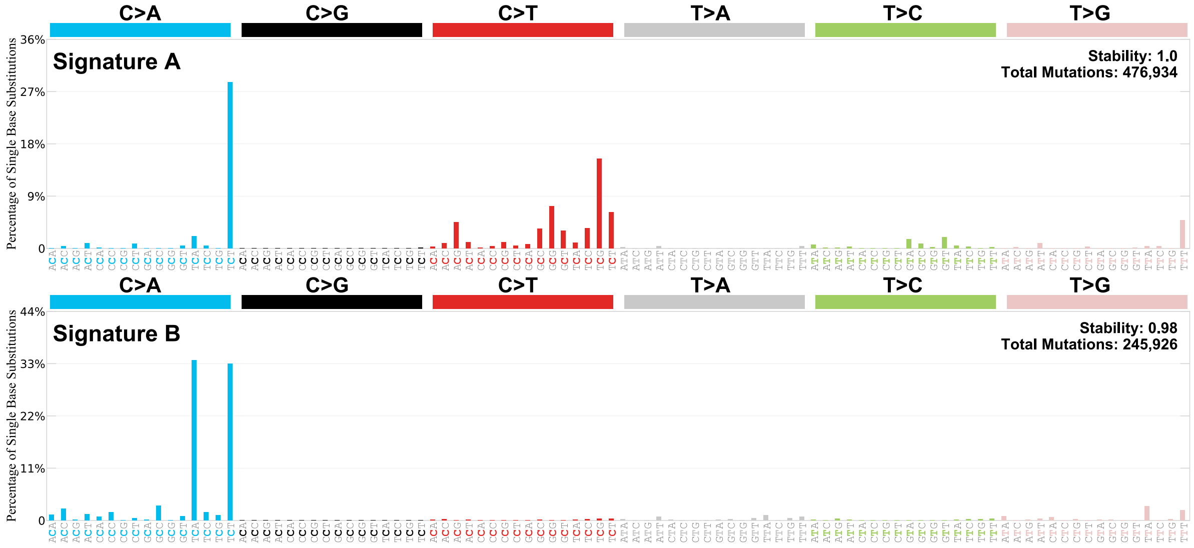

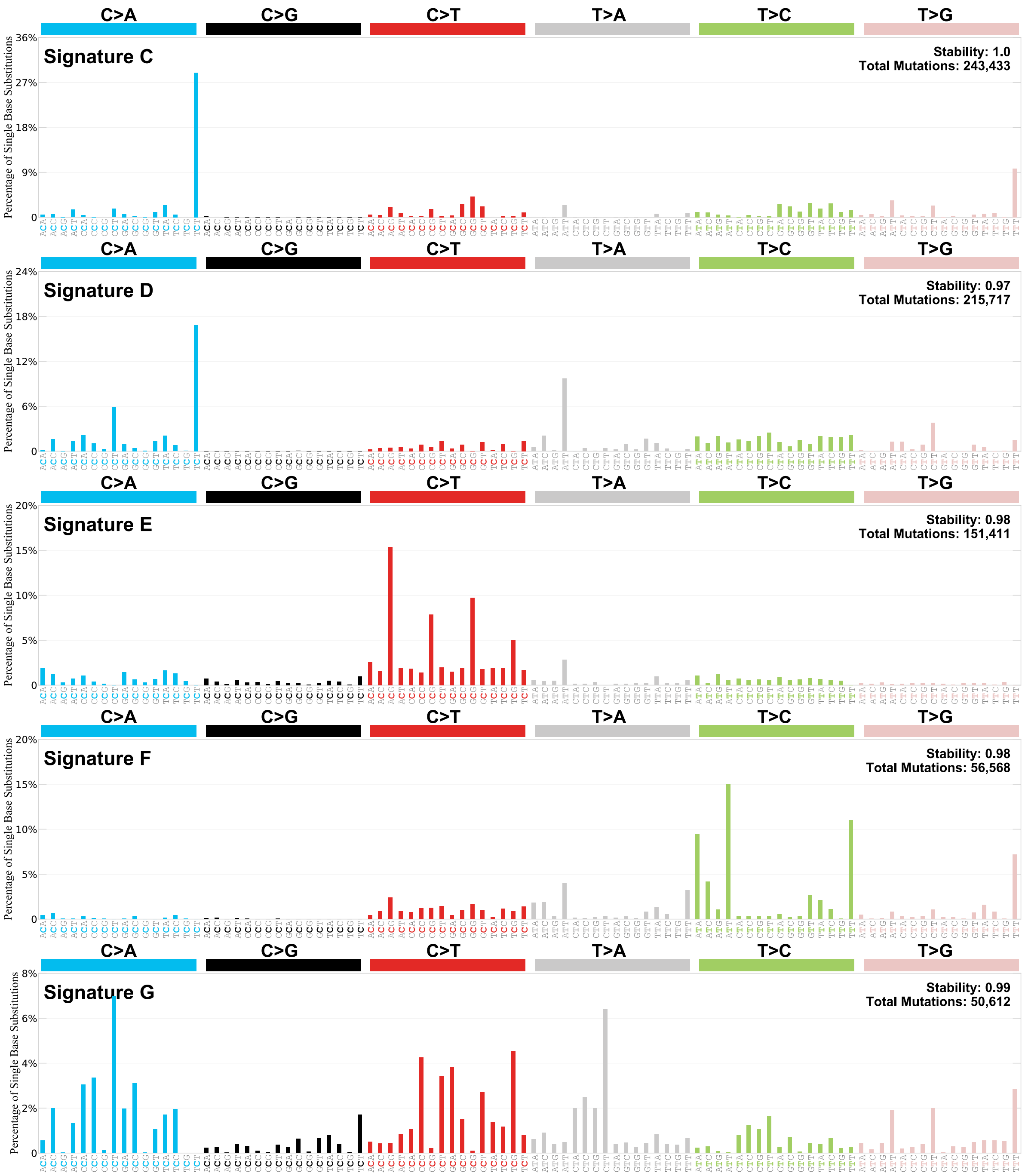
C>A
C>G
C>T
T>A
T>C
T>G
Percentage of Single Base Substitutions
36%
27%
18%
9%
0
Signature C
Stability: 1.0
Total Mutations: 243,433
24%
18%
12%
6%
0
Signature D
Stability: 0.97
Total Mutations: 215,717
20%
15%
10%
5%
0
Signature E
Stability: 0.98
Total Mutations: 151,411
20%
15%
10%
5%
0
Signature F
Stability: 0.98
Total Mutations: 56,568
8%
6%
4%
2%
0
Signature G
Stability: 0.99
Total Mutations: 50,612

Supplementary Information Figure 4 | Cosine similarity of HDP and SigProfiler signature components
Heatmap showing the cosine similarity between components extracted using the HDP algorithm and SigProfiler. SigProfiler recommends an optimum solution based on stability, this solution was used for the comparison.

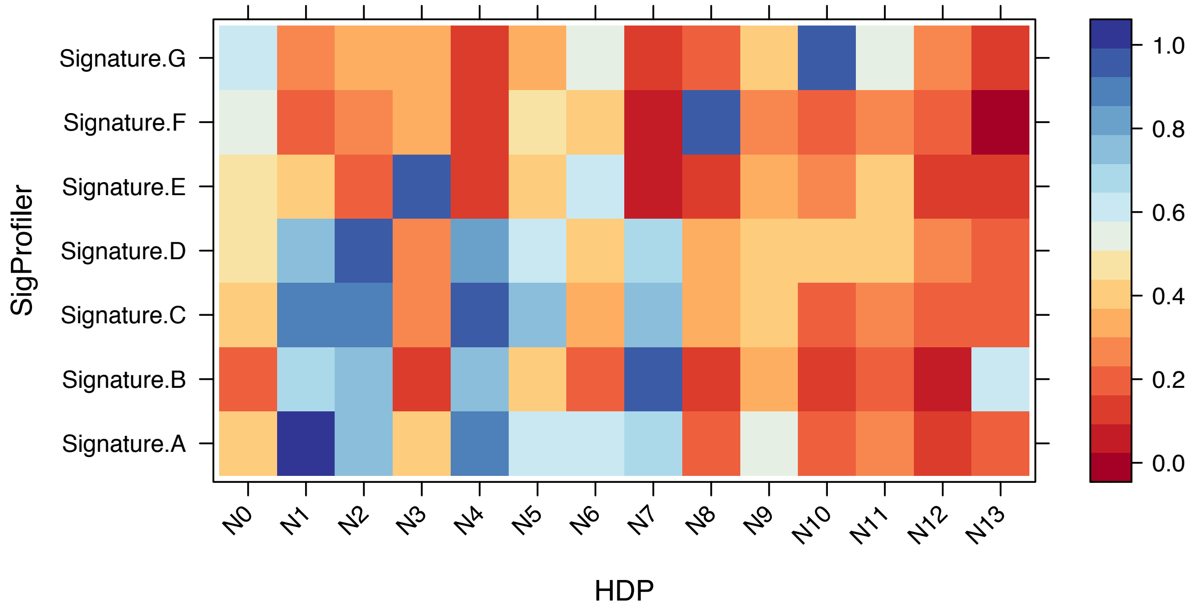

Supplementary Information Figure 5 | HDP combined signature extraction

Components are shown in full below. Plots show mutational spectra of extracted components showing trinucleotide context (x-axis) and proportion (y-axis). Credibility intervals (95%) are displayed as a thin black line above and below each bar. Trinucleotide contexts whose contribution is not statistically significant are shown as light grey bars.

Component 0 (SNV:89.2%)

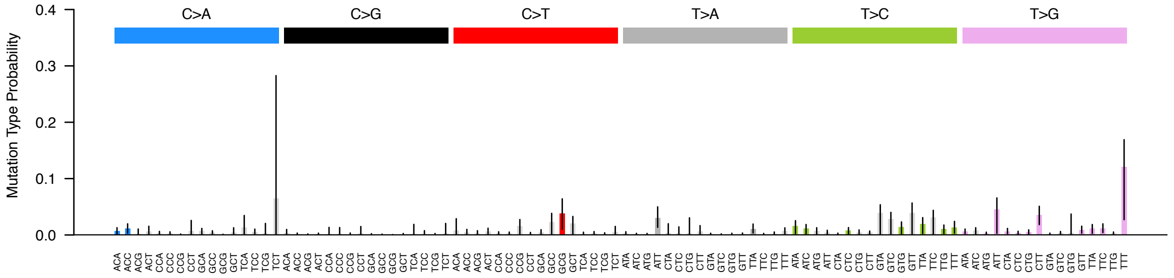

Component 0 (Indel:10.5%)

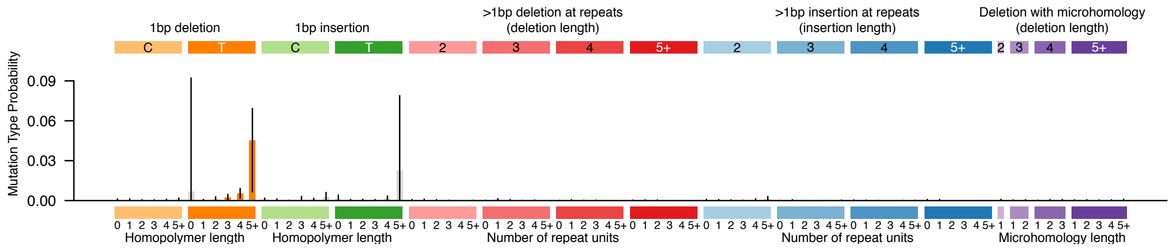

Component 0 (DNV:0.3%)

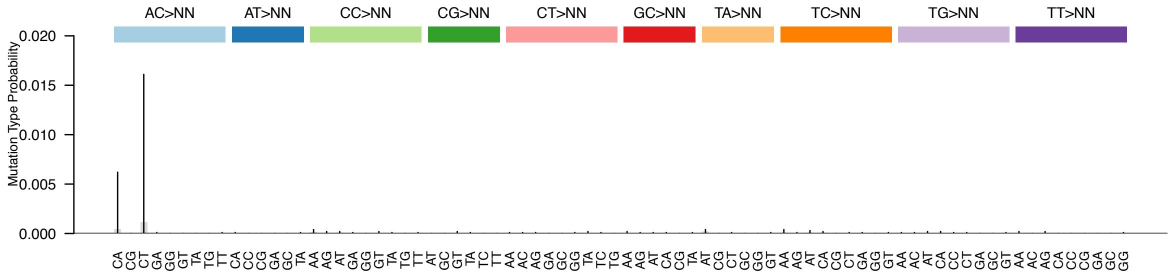

Component 1 (SNV:97.4%)

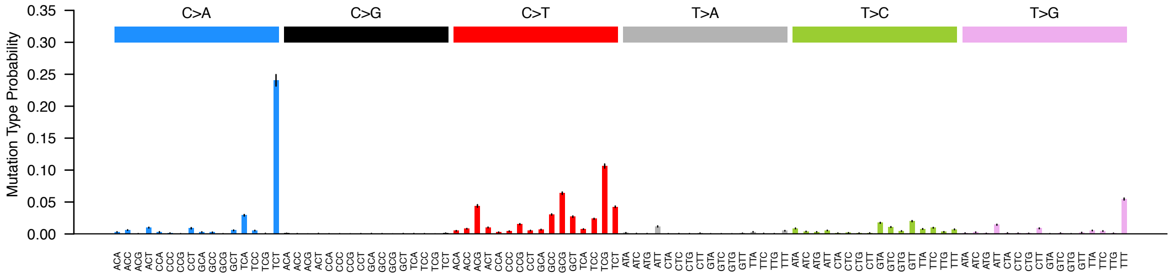

Component 1 (Indel:2.6%)

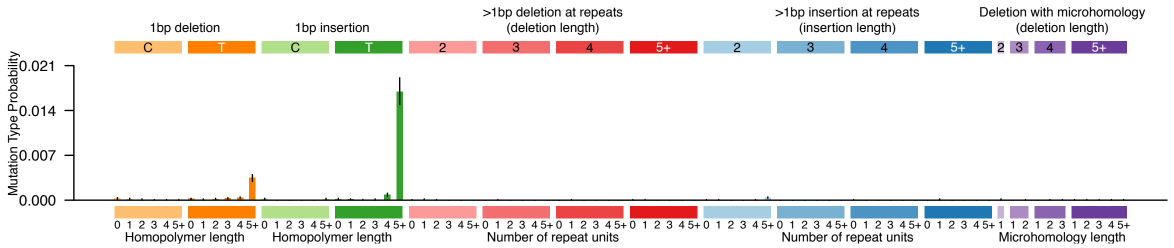

Component 1 (DNV:0%)

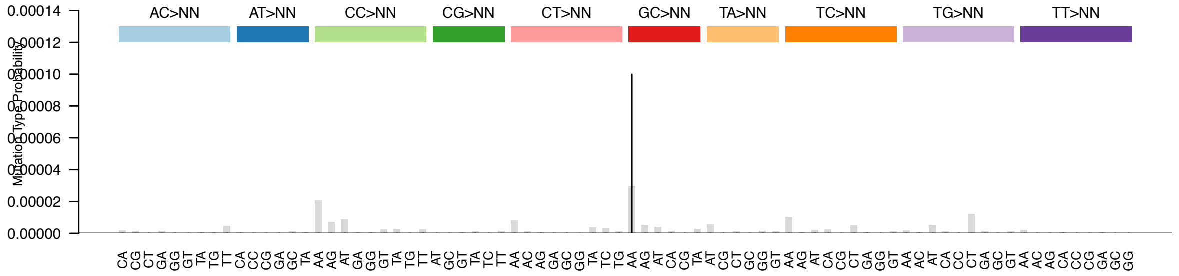

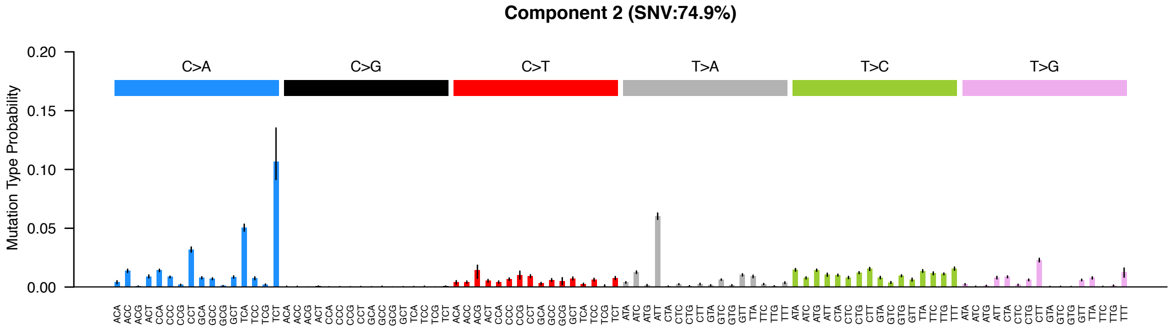
Component 2 (SNV:74.9%)
Mutation Type Probability
0.20
0.15
0.10
0.05
0.00
C>A
C>G
C>T
T>A
T>C
T>G

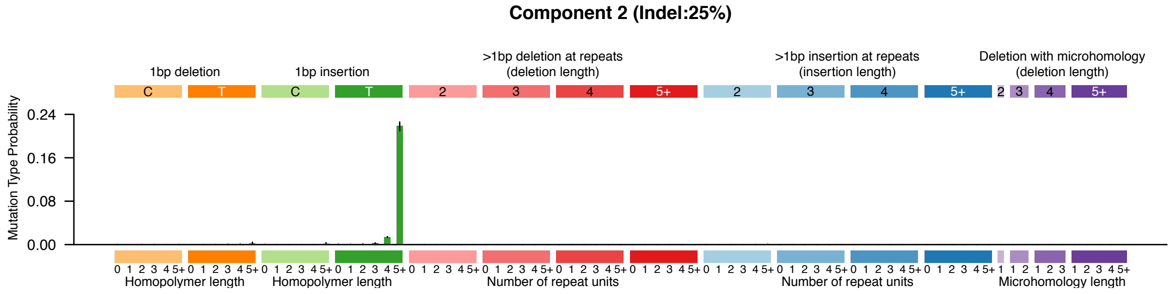
Component 2 (Indel:25%)
1bp deletion
1bp insertion
>1bp deletion at repeats (deletion length)
>1bp insertion at repeats (insertion length)
Deletion with microhomology (deletion length)
Mutation Type Probability
0.24
0.16
0.08
0.00
Homopolymer length
Homopolymer length
Number of repeat units
Number of repeat units
Microhomology length

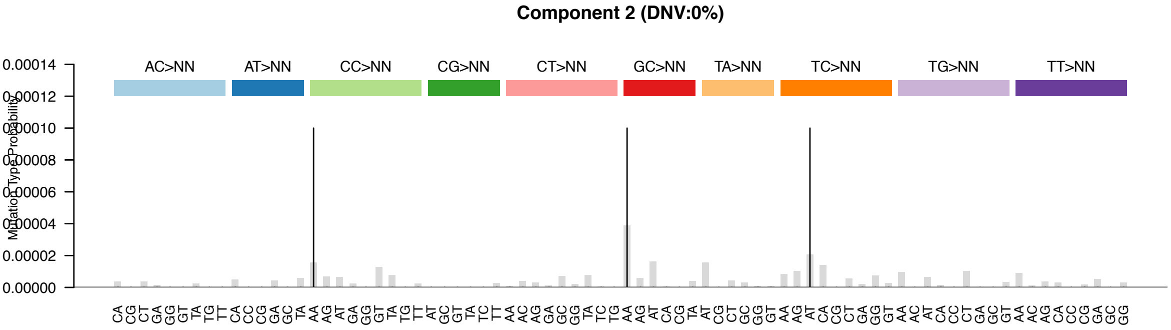
Component 2 (DNV:0%)
Mutation Type Probability
0.00014
0.00012
0.00010
0.00008
0.00006
0.00004
0.00002
0.00000
AC>NN
AT>NN
CC>NN
CG>NN
CT>NN
GC>NN
TA>NN
TC>NN
TG>NN
TT>NN

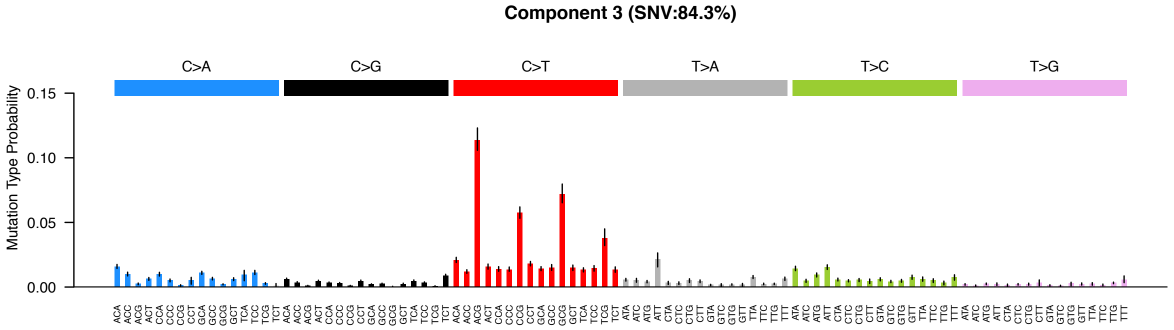
Component 3 (SNV:84.3%)
C>A
C>G
C>T
T>A
T>C
T>G
Mutation Type Probability
0.15
0.10
0.05
0.00

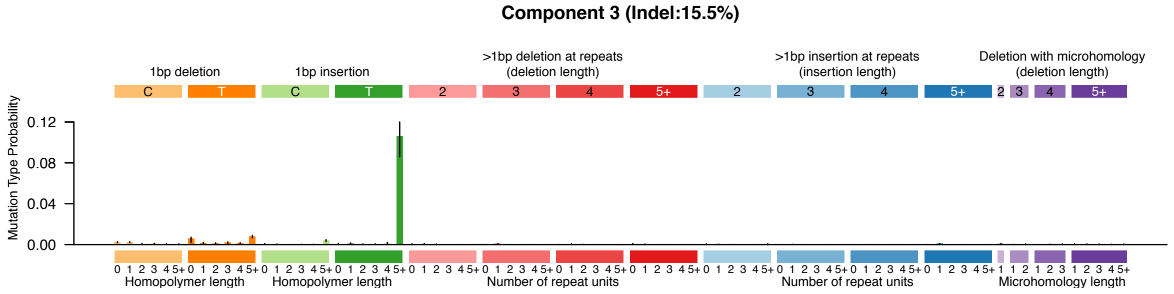
Component 3 (Indel:15.5%)
1bp deletion
1bp insertion
>1bp deletion at repeats (deletion length)
>1bp insertion at repeats (insertion length)
Deletion with microhomology (deletion length)
Mutation Type Probability
0.12
0.08
0.04
0.00
Homopolymer length
Homopolymer length
Number of repeat units
Number of repeat units
Microhomology length

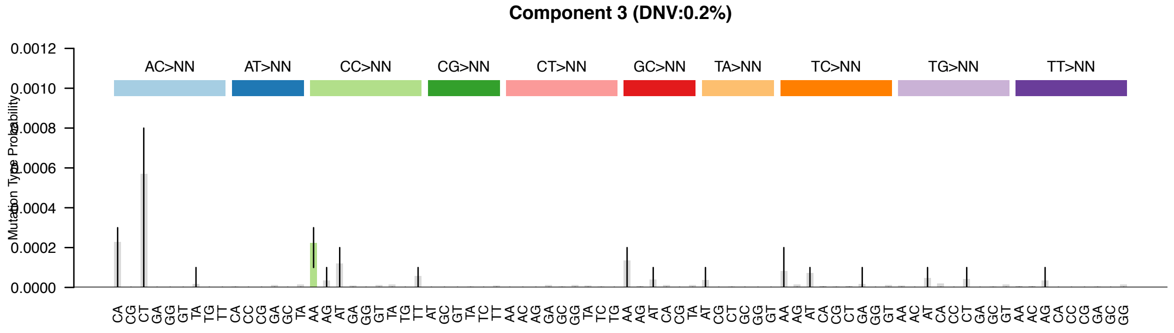
Component 3 (DNV:0.2%)
AC>NN
AT>NN
CC>NN
CG>NN
CT>NN
GC>NN
TA>NN
TC>NN
TG>NN
TT>NN
Mutation Type Probability
0.0012
0.0010
0.0008
0.0006
0.0004
0.0002
0.0000

Component 4 (SNV:96.8%)

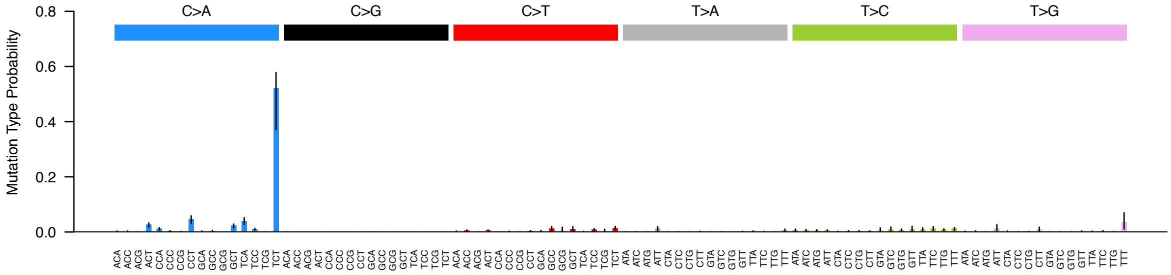

Component 4 (Indel:3.2%)

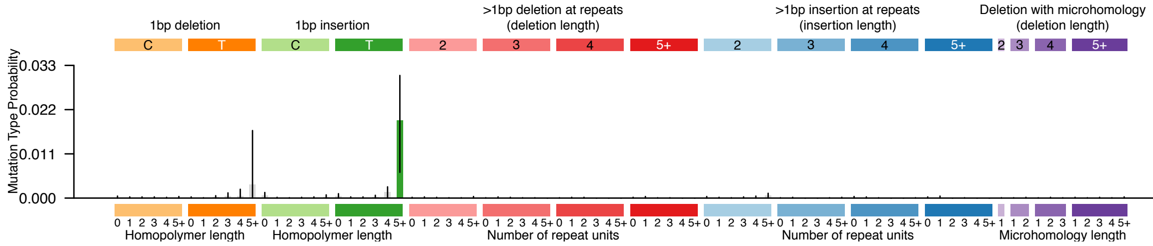

Component 4 (DNV:0%)

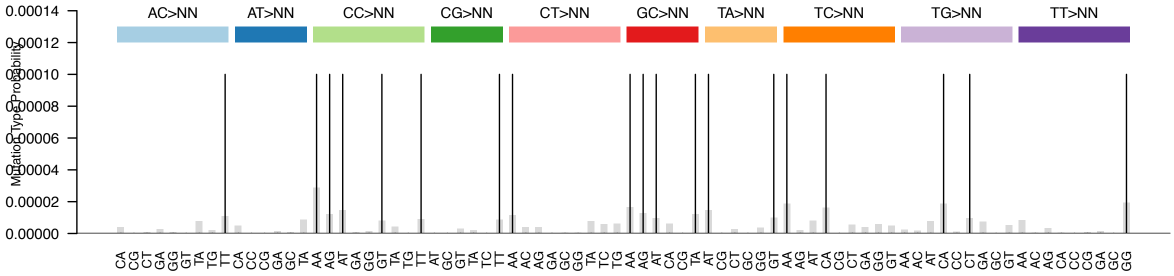

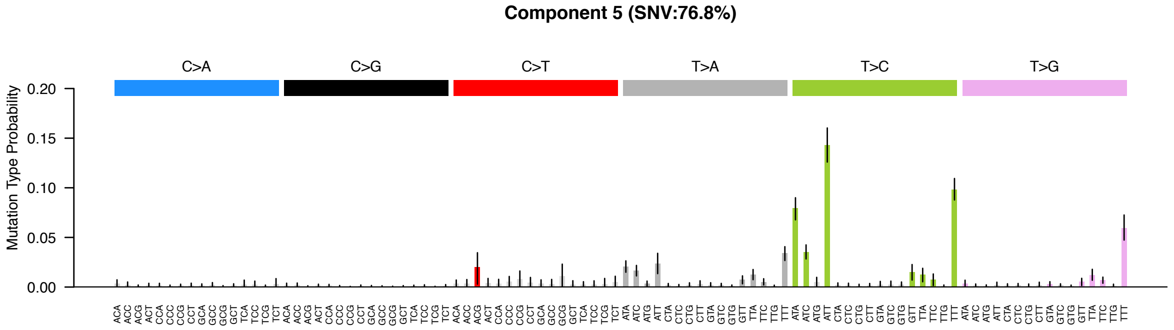
Component 5 (SNV:76.8%)
C>A
C>G
C>T
T>A
T>C
T>G
Mutation Type Probability
0.00
0.05
0.10
0.15
0.20

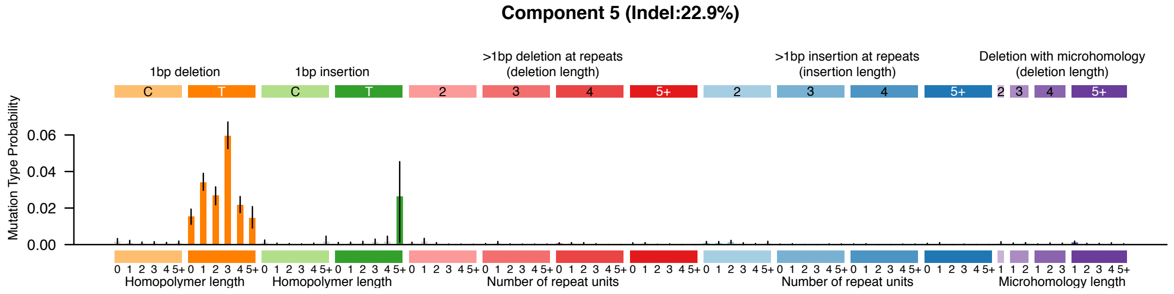
Component 5 (Indel:22.9%)
1bp deletion
1bp insertion
>1bp deletion at repeats (deletion length)
>1bp insertion at repeats (insertion length)
Deletion with microhomology (deletion length)
Mutation Type Probability
0.00
0.02
0.04
0.06
Homopolymer length
Homopolymer length
Number of repeat units
Number of repeat units
Microhomology length

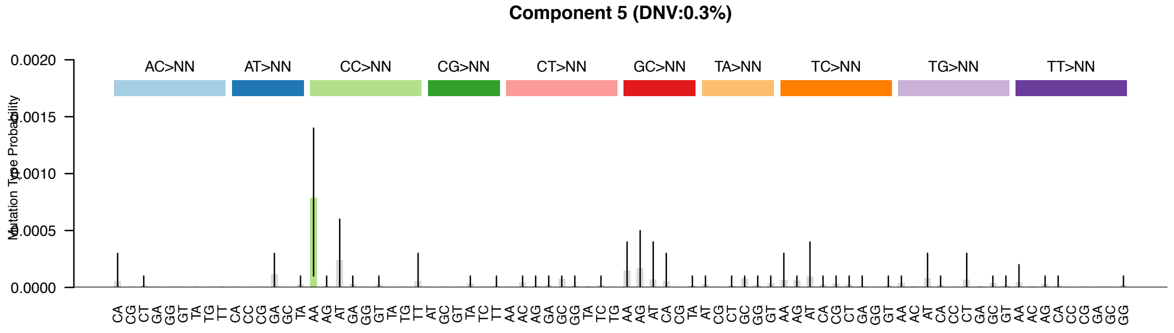
Component 5 (DNV:0.3%)
AC>NN
AT>NN
CC>NN
CG>NN
CT>NN
GC>NN
TA>NN
TC>NN
TG>NN
TT>NN
Mutation Type Probability
0.0000
0.0005
0.0010
0.0015
0.0020

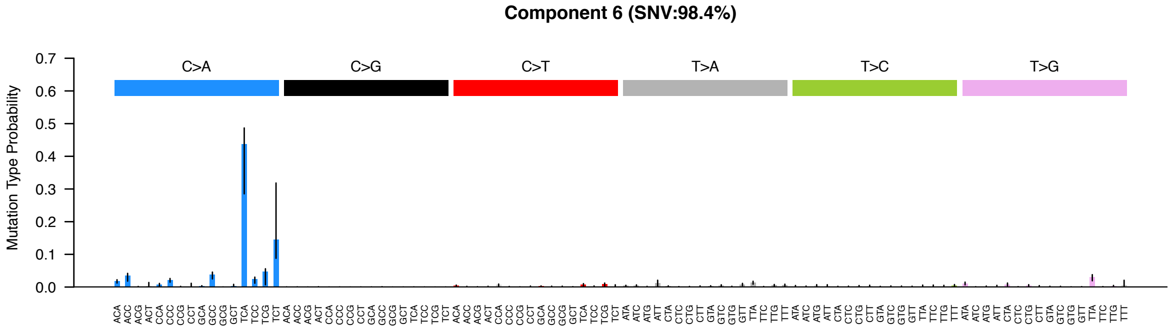
Component 6 (SNV:98.4%)
Mutation Type Probability
C>A
C>G
C>T
T>A
T>C
T>G

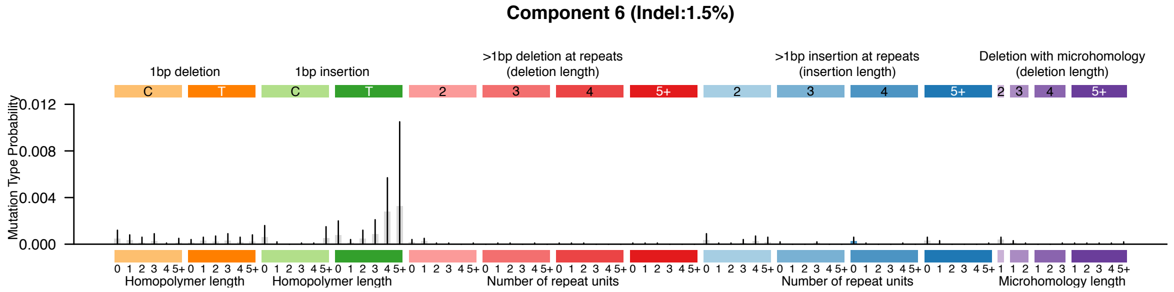
Component 6 (Indel:1.5%)
Mutation Type Probability
1bp deletion
1bp insertion
>1bp deletion at repeats (deletion length)
>1bp insertion at repeats (insertion length)
Deletion with microhomology (deletion length)
Homopolymer length
Homopolymer length
Number of repeat units
Number of repeat units
Microhomology length

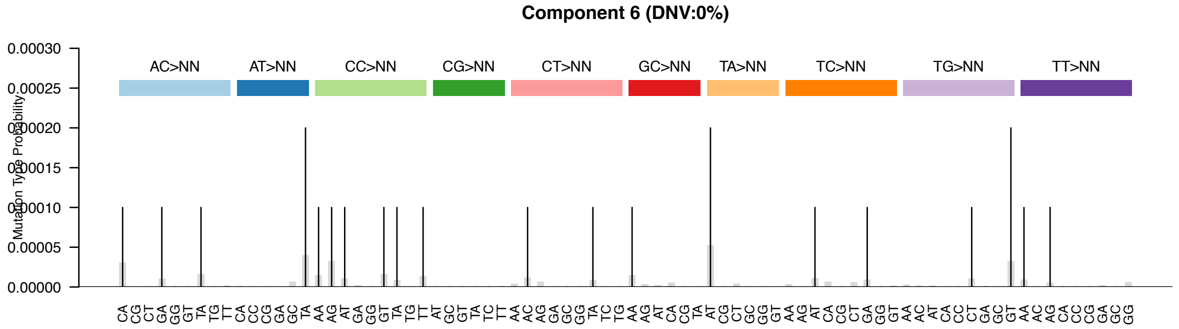
Component 6 (DNV:0%)
Mutation Type Probability
AC>NN
AT>NN
CC>NN
CG>NN
CT>NN
GC>NN
TA>NN
TC>NN
TG>NN
TT>NN

Component 7 (SNV:90.1%)

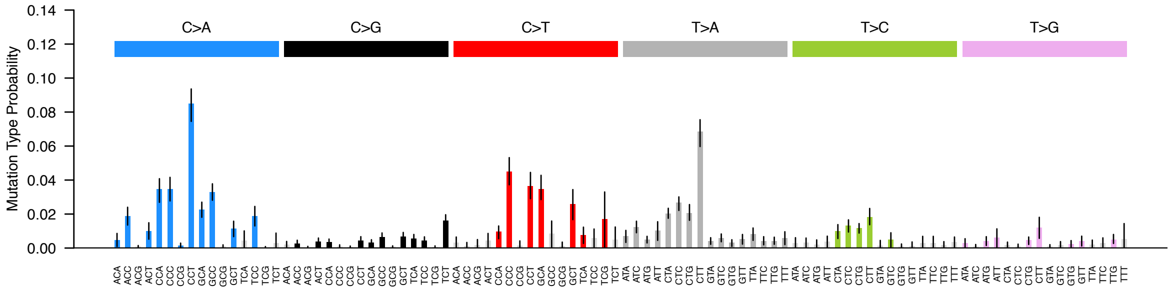

Component 7 (Indel:4.5%)

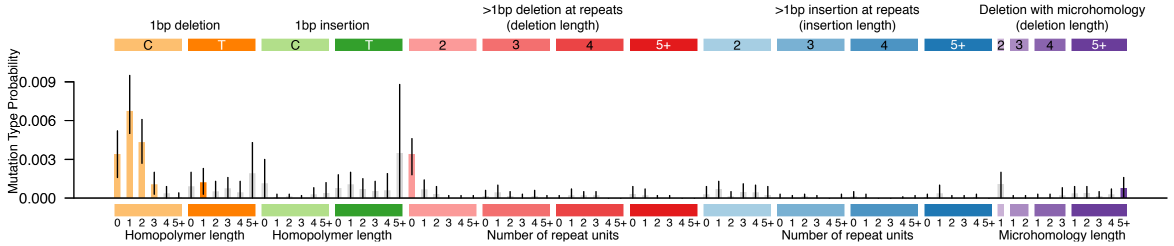

Component 7 (DNV:5.4%)

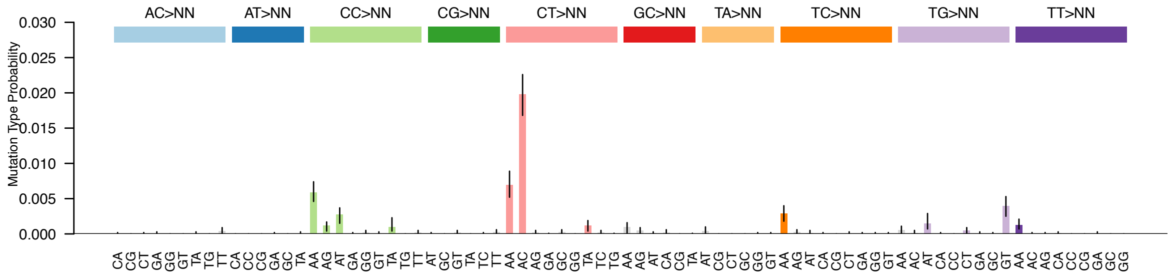

Component 8 (SNV:92.4%)

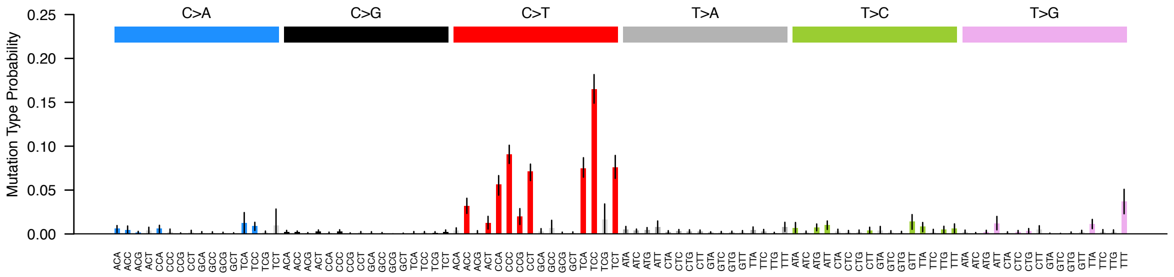

Component 8 (Indel:1.2%)

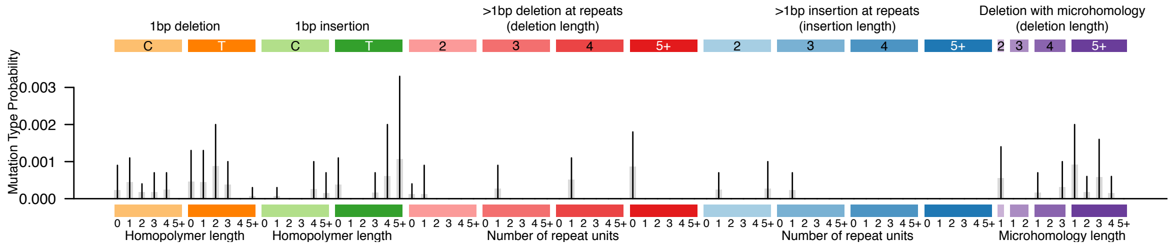

Component 8 (DNV:6.4%)

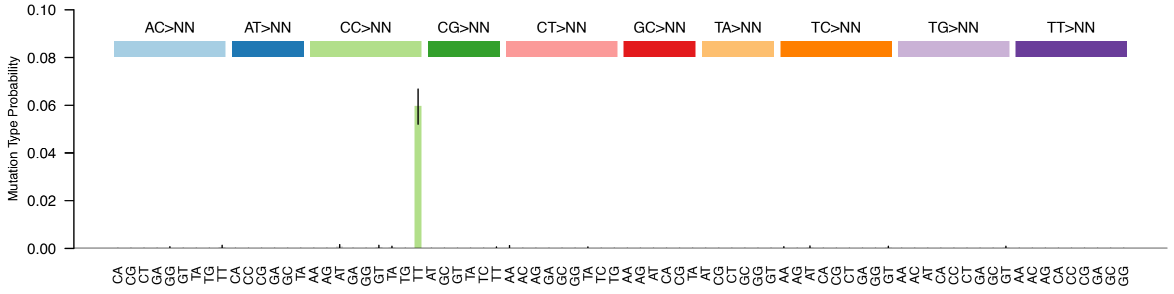

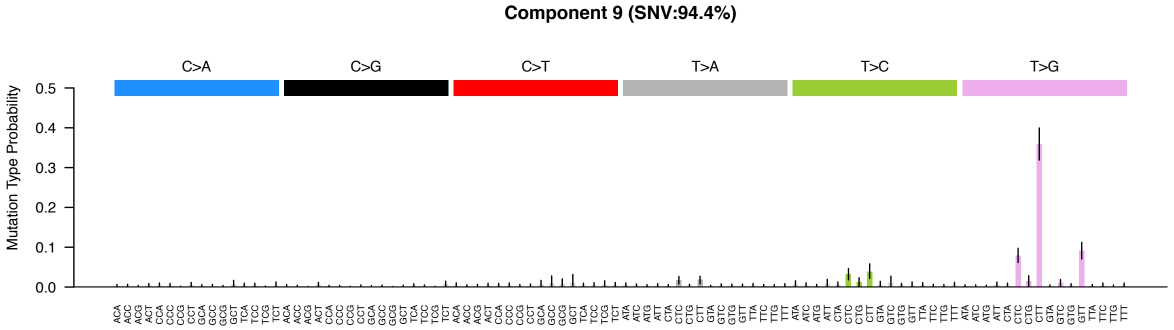
Component 9 (SNV:94.4%)
C>A
C>G
C>T
T>A
T>C
T>G
Mutation Type Probability

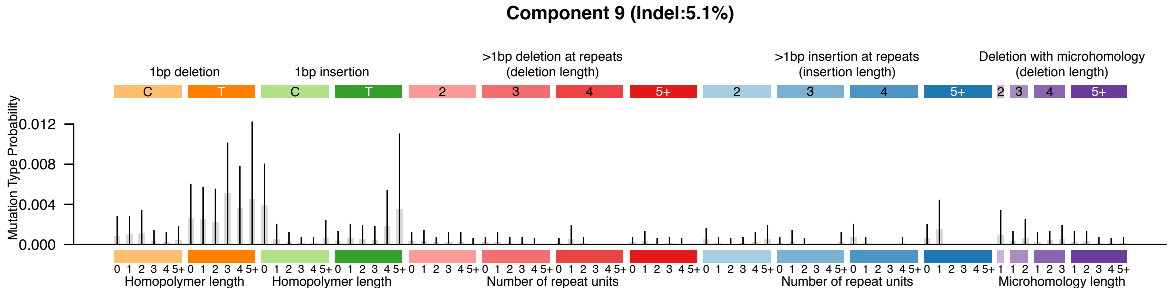
Component 9 (Indel:5.1%)
1bp deletion
1bp insertion
>1bp deletion at repeats (deletion length)
>1bp insertion at repeats (insertion length)
Deletion with microhomology (deletion length)
Mutation Type Probability
Homopolymer length
Homopolymer length
Number of repeat units
Number of repeat units
Microhomology length

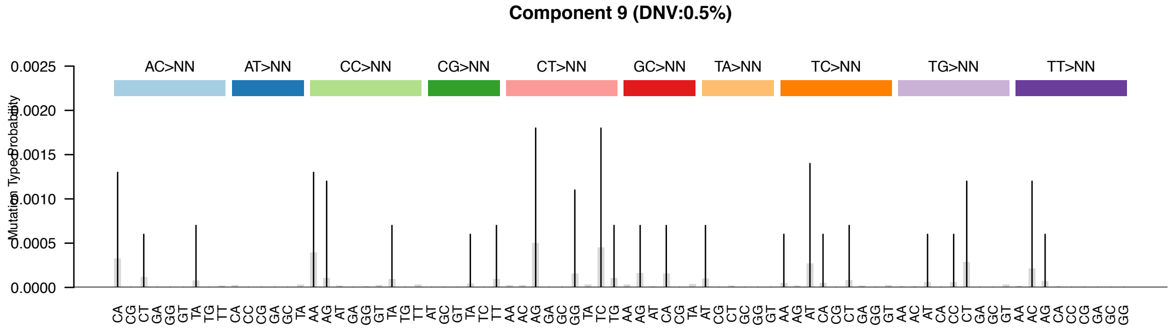
Component 9 (DNV:0.5%)
AC>NN
AT>NN
CC>NN
CG>NN
CT>NN
GC>NN
TA>NN
TC>NN
TG>NN
TT>NN
Mutation Type Probability

Component 10 (SNV:14%)

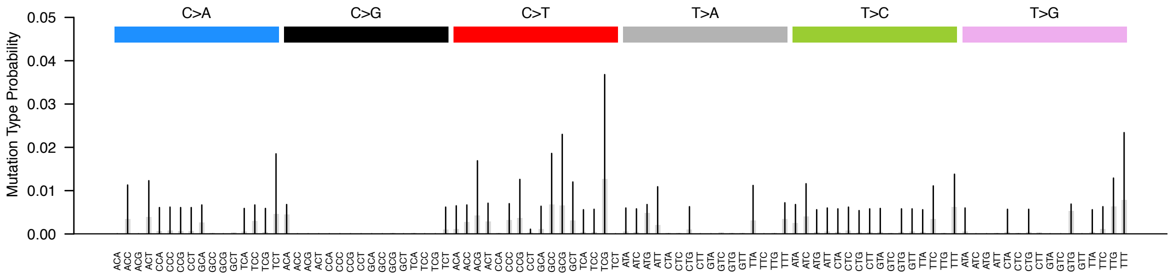

Component 10 (Indel:85.3%)

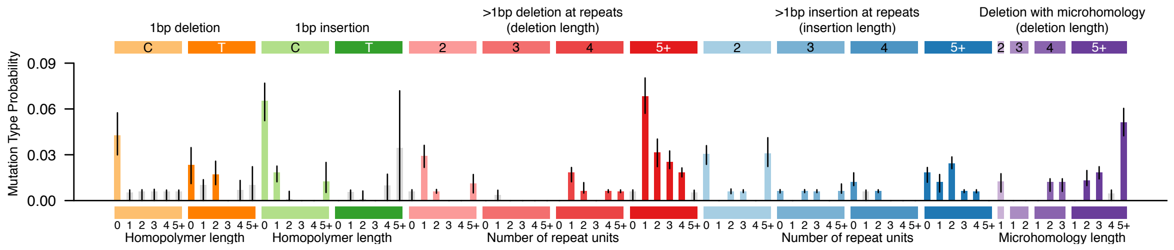

Component 10 (DNV:0.7%)

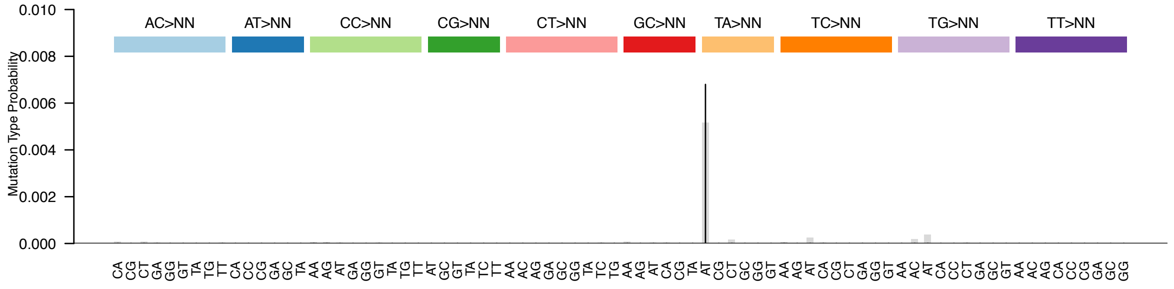

Supplementary Information Fig. 6 | Characterisation of mutational signature SBS91

(a) Mutational profiles of samples with predominant SBS91 exposure; 3 representative cerebral cortex samples (PD44594c_lo0005, PD44594c_lo0001 and PD44594c_lo0007) and an oesophagus sample (PD44594h_lo0001). **(b)** Histograms of Variant Allele Fraction (VAF) for SBS mutations corresponding to each sample. **(c)** Mutational profile showing replication strand bias of SBS91 mutations. Coloured bars indicate leading strand and light grey bars indicate lagging strand. No statistically significant strand asymmetry is seen in SBS91 **(d)** Barplot of mutations on the transcribed (T) and un-transcribed (U) strands arranged according to mutation type. No statistically significant transcriptional strand bias was observed. **(e)** Histogram showing SBS91 mutation count across replication timing bins **(f)** JBrowse images showing SBS91 somatic mutations validated across multiple independent samples. **(g)** Histology image of cerebral cortex displayed in perspective view. Tissue microbiopsies are indicated by shapes with black outlines and annotated with numbers 1,2,5,6,7 corresponding to biopsies PD44594c_lo0001, PD44594c_lo0002, PD44594c_lo0005, PD44594c_lo0006 and PD44594c_lo0007. Phylogeny of SBS mutations is overlaid to illustrate the region of highest mutation density (biopsies 5,6 and 7). **(h)** Extended sequence context of mutations assigned to signature SBS91 displayed with pyrimidine annotation **(i)** Proportion of somatic mutations assigned to each mutational signature that are in Alu repeats. **(j)** Barplot of mutations assigned to SBS91 that are in Alu repeats (Alu) and those that are elsewhere in the genome (non-Alu). Mutations occurring with the canonical extended sequence context GGGTGGTCTC or its reverse complement GAGACCACCC (mutant base underlined) are represented by the green bars and those occurring outside of the canonical repeat are purple. P-value calculated with the two-sided Pearson's Chi-squared test.

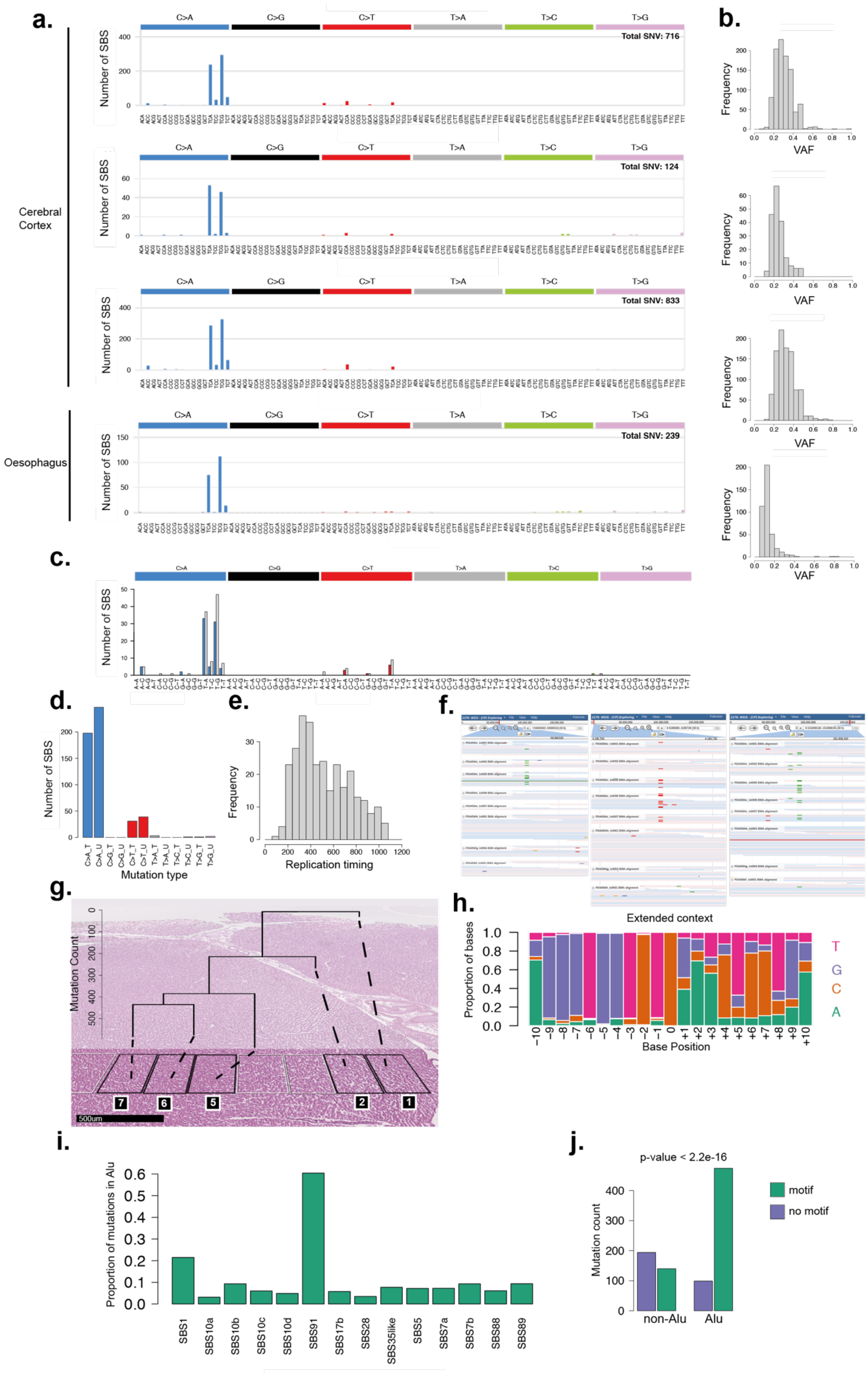
a.
b.
Cerebral Cortex
Oesophagus
C>A
C>G
C>T
T>A
T>C
T>G
Total SNV: 716
Total SNV: 124
Total SNV: 833
Total SNV: 239
Number of SBS
Frequency
VAF
c.
d.
Mutation type
e.
Replication timing
f.
g.
Mutation Count
500um
h.
Extended context
Proportion of bases
Base Position
T
G
C
A
i.
Proportion of mutations in Alu
SBS1
SBS10a
SBS10b
SBS10c
SBS10d
SBS91
SBS17b
SBS28
SBS35like
SBS5
SBS7a
SBS7b
SBS88
SBS89
j.
p-value < 2.2e-16
Mutation count
motif
no motif
non-Alu
Alu

Supplementary Information Fig. 7 | Protein structure and mutation site of POLD1 and POLE polymerases

Mutations carried by individuals included in this study mapped onto the Saccharomyces cerevisiae DNA polymerase δ and DNA polymerase ε

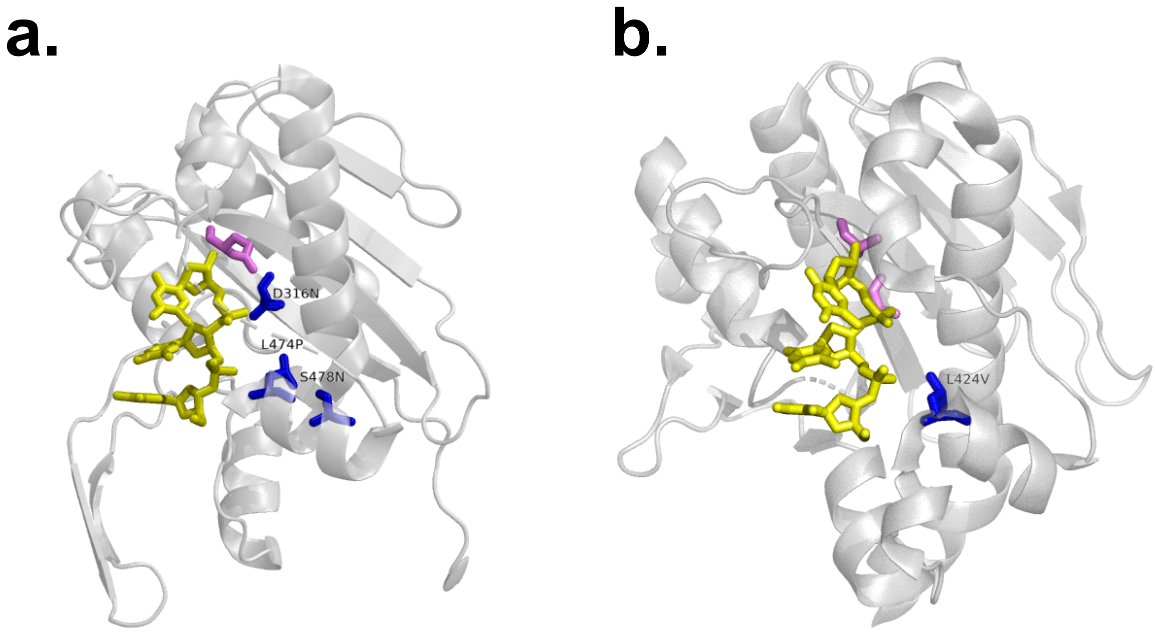

Site of germline DNA polymerase mutations in this cohort mapped (blue) onto the protein structure of the exonuclease domains (grey) of **(a)** Saccharomyces cerevisiae Pol δ (Protein Data Bank identifier (PDB ID) 3IAY) and **(b)** Pol ε (PBD ID 4M8O). Single-stranded DNA from the aligned bacteriophage T4 polymerase complex (PDB ID 1NOY117) is shown in yellow. The exo I motif active site residues are highlighted in magenta (with the exception of mutated Pol δ active site residue D316, which is also the site of one of the germline mutations carried by an individual in this study).
