# Supplementary material for: Increased somatic mutation burdens in normal human cells due to defective DNA polymerases
Source: Nat Genet. 2021 Sep 30;53(10):1434–42. doi: 10.1038/s41588-021-00930-y (PMC8492474; doi:10.1038/s41588-021-00930-y)
Supplement: Supplementary file 2 — Reporting Summary [file 41588_2021_930_MOESM2_ESM.pdf]

nature research

Corresponding author(s): Michael R. Stratton
Ian Tomlinson

Last updated by author(s): 23/03/2021

Reporting Summary

Nature Research wishes to improve the reproducibility of the work that we publish. This form provides structure for consistency and transparency in reporting. For further information on Nature Research policies, see our Editorial Policies and the Editorial Policy Checklist.

Statistics

For all statistical analyses, confirm that the following items are present in the figure legend, table legend, main text, or Methods section.

n/a	Confirmed	
☐	☒	The exact sample size (*n*) for each experimental group/condition, given as a discrete number and unit of measurement
☐	☒	A statement on whether measurements were taken from distinct samples or whether the same sample was measured repeatedly
☐	☒	The statistical test(s) used AND whether they are one- or two-sided<br>*Only common tests should be described solely by name; describe more complex techniques in the Methods section.*
☐	☒	A description of all covariates tested
☐	☒	A description of any assumptions or corrections, such as tests of normality and adjustment for multiple comparisons
☐	☒	A full description of the statistical parameters including central tendency (e.g. means) or other basic estimates (e.g. regression coefficient) AND variation (e.g. standard deviation) or associated estimates of uncertainty (e.g. confidence intervals)
☐	☒	For null hypothesis testing, the test statistic (e.g. F, t, r) with confidence intervals, effect sizes, degrees of freedom and P value noted<br>*Give P values as exact values whenever suitable.*
☐	☒	For Bayesian analysis, information on the choice of priors and Markov chain Monte Carlo settings
☒	☐	For hierarchical and complex designs, identification of the appropriate level for tests and full reporting of outcomes
☒	☐	Estimates of effect sizes (e.g. Cohen's *d*, Pearson's *r*), indicating how they were calculated

Our web collection on statistics for biologists contains articles on many of the points above.

Software and code

Policy information about availability of computer code

Data collection	No software was used in data collection
Data analysis	Somatic mutation calling was performed using the Wellcome Sanger Institute's mutation calling algorithms as part of a bespoke pipeline. Single Base Substitutions were called using CaVEMan (cancer variants through expectation maximization) algorithm (1.13.14 and 1.14.1). Small insertions and deletions (ID) were called using Pindel algorithm (3.3.0). Rearrangements were called using BRASS (breakpoint via assembly) algorithm (6.3.0). Algorithms are publicly available through the Cancer IT / CASM GitHub page https://github.com/cancerit.<br><br>Custom algorithms used in filtering somatic mutations are available from https://github.com/TimCoorens<br><br>Data analysis was performed in R (3.4.0 and 3.4.1). Code for statistical modeling is available in the supplementary material.<br>Additional code is available on request form the authors.<br>Open source R packages used include:<br>Package Version repo<br>ape 5.3 CRAN<br>ASCAT 2.5 GitHub<br>BiocManager 1.30.10 CRAN<br>BiocStyle 2.6.1 Bioconductor<br>BSgenome 1.46.0 Bioconductor<br>BSgenome.Hsapiens.UCSC.hg19 1.4.0 Bioconductor<br>data.table 1.12.8 CRAN<br>devtools 2.2.2 CRAN<br>dndscv 0.0.1.0 GitHub<br>dplyr 0.8.5 CRAN

GenomicRanges 1.30.3 Bioconductor
ggtree 1.10.5 Bioconductor
hdp 0.1.5 GitHub
Rsamtools 1.30.0 Bioconductor
seqinr 3.6-1 CRAN
sigfit 2.0.0 GitHub
stringr 1.4.0 CRAN
tidyr 1.0.2 CRAN
VGAM 1.1-2 CRAN
xlsx 0.6.3 CRAN
BSgenome 1.46.0 Bioconductor
BSgenome.Hsapiens.1000genomes.hs37d5 0.99.1 Bioconductor
data.table 1.10.4-3 CRAN
dbplyr 1.2.1 CRAN
forcats 0.3.0 CRAN
GenomicAlignments 1.14.2 Bioconductor
GenomicFeatures 1.30.3 Bioconductor
GenomicRanges 1.30.3 Bioconductor
ggdendro 0.1-20 CRAN
ggplot2 2.2.1 CRAN
IRanges 2.12.0 Bioconductor
knitr 1.2 CRAN
RColorBrewer 1.1-2 CRAN
Rsamtools 1.30.0 Bioconductor
tidyverse 1.2.1 CRAN
viridis 0.5.1 CRAN
viridisLite 0.3.0 CRAN

Other software:
Telomerehunter 1.1.0 (2015) PIP in a Python 2.7 virtual environment

SigProfiler was installed in a conda3 environment
The following versions of SigProfiler dependencies were installed:
Python Version: 3.7.4
Sigproextractor Version: 0.0.5.76
SigprofilerPlotting Version: 1.0.8
SigprofilerMatrixGenerator Version: 1.0.21
Pandas version: 0.25.1
Numpy version: 1.17.2
Scipy version: 1.3.1
Scikit-learn version: 0.21.3
Nimfa version: 1.4.0

For manuscripts utilizing custom algorithms or software that are central to the research but not yet described in published literature, software must be made available to editors and reviewers. We strongly encourage code deposition in a community repository (e.g. GitHub). See the Nature Research guidelines for submitting code & software for further information.

Data

Policy information about availability of data

All manuscripts must include a data availability statement. This statement should provide the following information, where applicable:

- Accession codes, unique identifiers, or web links for publicly available datasets
- A list of figures that have associated raw data
- A description of any restrictions on data availability

DNA sequencing data are deposited in the European Genome-Phenome Archive (EGA) with accession code: EGAD00001006212
DNA sequencing data from the modified duplex sequencing are deposited in the EGA with accession code: EGAS00001004066
Somatic mutations and mutational signature data from this cohort are available online (https://github.com/TimCoorens/Polymerase)
All other data are available from the authors on request.
The cBioPortal MutationMapper database was accessed at: https://www.cbioportal.org/mutation_mapper?standaloneMutationMapperGeneTab=ATM

Field-specific reporting

Please select the one below that is the best fit for your research. If you are not sure, read the appropriate sections before making your selection.

☒ Life sciences ☐ Behavioural & social sciences ☐ Ecological, evolutionary & environmental sciences

For a reference copy of the document with all sections, see nature.com/documents/nr-reporting-summary-flat.pdf

Life sciences study design

All studies must disclose on these points even when the disclosure is negative.

Sample size	No formal sample size calculations were performed. A limited number of cases /individuals were available given the rarity of the germline mutation. A diverse selection of individuals carrying mutations across the two main DNA polymerase genes were selected.
Data exclusions	One endometrial gland (PD44589f_lo0008) was removed from analyses of SBS mutation rate and from the endometrial gland phylogenetic tree as it demonstrated clear evidence of clonal mixing.
Replication	This study and its experiments were designed to ensure that each germline mutation / gene studied was represented by multiple samples per patient and where possible multiple patients per affected germline gene / genotype. Validation of the DNA isolation, library preparation and sequencing of low-DNA input samples has been undertaken for intestinal crypts and endometrial glands as part of the studies published in Ellis et al 2020, Lee Six et al 2019 and Moore et al 2020. In these experiments biological replicates were obtained for individuals crypts (serial sections from n=17 crypts) and endometrial glands (serial sections from n=18 glands). These replicates were independently isolated and processed and demonstrated good concordance. Further detail of the low-input method are detailed in Ellis et al Nature protocols 2021. Replication of the modified duplex sequencing protocol used to sequence blood and sperm were performed as part of its development and are detailed in its manuscript (Abascal et al 2021).
Randomization	Not applicable - this study did not involve an intervention and as such no randomization was undertaken. Covariates such as age, germline mutation in the DNA polymerase genes and sequencing parameters were controlled for with statistical modeling (Supplementary Note).
Blinding	Not applicable - this study did not involve the allocation to groups and hence blinding was not performed.

Reporting for specific materials, systems and methods

We require information from authors about some types of materials, experimental systems and methods used in many studies. Here, indicate whether each material, system or method listed is relevant to your study. If you are not sure if a list item applies to your research, read the appropriate section before selecting a response.

Materials & experimental systems

n/a	Involved in the study	
☒	☐	Antibodies
☒	☐	Eukaryotic cell lines
☒	☐	Palaeontology and archaeology
☒	☐	Animals and other organisms
☐	☒	Human research participants
☒	☐	Clinical data
☒	☐	Dual use research of concern

Methods

n/a	Involved in the study	
☒	☐	ChIP-seq
☒	☐	Flow cytometry
☒	☐	MRI-based neuroimaging

Human research participants

Policy information about studies involving human research participants

Population characteristics	This study involves analysis of samples from individuals aged 17-72 years who have germline DNA polymerase exonuclease domain mutations. Analysis of other conditions and treatments was not performed.
Recruitment	Individuals were recruited to the CORGI studies under United Kingdom Research Ethics Committee approval 17/SC/0079.<br><br>1. Endoscopy samples<br>Additional gastrointestinal tissue biopsies were collected for the purposes of research during the course of routine clinical endoscopy under informed consent.<br><br>2. Surgical resection samples<br>Tissue samples for research were isolated from the resection margins of tissue specimens that were removed during surgery. These samples were taken from samples that would have otherwise gone into clinical waste. Sampling of tissue from the surgically resected tissue was undertaken by a trained specialist to ensure that samples taken for the purposes of research did not in any way affect routine histopathology assessment of the resection samples. Informed consent was obtained prior to collection.<br><br>3. Autopsy<br>Multiple tissues were collected from a single individual following autopsy under informed consent according to the protocol outlined in the REC approval (cited above). This collection allowed for study of tissues that could not be collected by other means (e.g. cerebral cortex) and allowed for multiple samples to be collected from a single individual which would not be possible with the other methods of sample collection outlined above.<br><br>4. Additional samples

Blood and sperm samples were using routine methods according the the protocol submitted to the REC.

Individuals recruited to this study were initially identified in the CORGI study. They presented in a clinical setting with intestinal neoplasia. The study population comprises individuals identified through CORGI and their relatives who were subsequently confirmed as carrying the respective DNA polymerase germline mutation. Linkage analysis was performed as part of the first study which confirms the association between the germline mutation and the cancer predisposition which is the main feature of this syndrome (Palles et al 2013). Whilst it might be plausible that there is a selection bias attributable to the identification of individuals with intestinal neoplasia, we can confirm that all individuals who have confirmed pathogenic variants in POLE and POLD1 have a substantially elevated risk of cancer whether they had intestinal neoplasia at their initial presentation or not. There is a substantial life time risk of intestinal neoplasia in 'asymptomatic' individuals identified via family tree linkage.

Ethics oversight

This research complies with all relevant ethical regulations. Patients were recruited as part of the CORGI-2 study United Kingdom Research Ethics Committee (REC) 17/SC/0079. Additional sample collection was undertaken under approval from the following committees; London – Westminster, North East-Newcastle and North Tyneside 1 and NRES Committee East of England - Cambridge South (REC references: EC04/015, 16/NE/003 and 07-MRE05-44 respectively). Informed consent was obtained from all participants and no monetary compensation was offered for their participation.

Note that full information on the approval of the study protocol must also be provided in the manuscript.
