# Supplementary material for: Increased somatic mutation burdens in normal human cells due to defective DNA polymerases
Source: Nat Genet. 2021 Sep 30;53(10):1434–42. doi: 10.1038/s41588-021-00930-y (PMC8492474; doi:10.1038/s41588-021-00930-y)
Supplement: Supplementary file 4 — Peer Review Information [file 41588_2021_930_MOESM4_ESM.pdf]

natureresearch

Peer Review Information

Journal: Nature Genetics
Manuscript Title: Increased somatic mutation burdens in normal human cells due to defective DNA polymerases
Corresponding author name(s): Professor Michael Stratton

Editorial Notes:

**Transferred manuscripts**	This manuscript has been previously reviewed at another journal that is not operating a transparent peer review scheme. This document only contains reviewer comments, rebuttal and decision letters for versions considered at Nature Genetics.

Reviewer Comments & Decisions:

Decision Letter, initial version:

Our ref: NG-A56992

17th Mar 2021

Dear Mike,

I hope you're well.

First of all, please accept my apologies for the delay in returning this decision to you. Your patience is appreciated.

Thank you for submitting your revised manuscript "Elevated somatic mutation burdens in normal human cells due to defective DNA polymerases" (NG-A56992). It has now been seen by the original referees and their comments are below. The reviewers find that the paper has improved in revision, and therefore we'll be happy in principle to publish it in Nature Genetics, pending revisions to satisfy Reviewer #2's final requests (these are minor) and to comply with our editorial and formatting guidelines.

If the current version of your manuscript is in a PDF format, please email us a copy of the file in an

editable format (Microsoft Word or LaTex)-- we can not proceed with PDFs at this stage.

We are now performing detailed checks on your paper and will send you a checklist detailing our editorial and formatting requirements in about a week. Please do not upload the final materials and make any revisions until you receive this additional information from us.

Thank you again for your interest in Nature Genetics. It's a lovely paper and I look forward to seeing it online and in print.

Kind regards,

Safia

Safia Danovi
Senior Editor
Nature Genetics

[Ed; please note that we have used the same numbering of reviewers as in your previous round. Note also that Reviewer #1 has signed their report]

Reviewer #1 (Remarks to the Author):

The authors have made a good job in addressing the comments by the reviewers and the new version of the manuscript has improved. I am satisfied with their answers to comments and no further changes are requested from my side.

Nuria Lopez-Bigas

Reviewer #2 (Remarks to the Author):

In their manuscript, Robinson et al. described a landscape of somatic mutations caused by defective DNA polymerase in patients with germline mutations in POLE and POLD1. The authors fully address the concerns raised by the reviewers for their original submission. The manuscript has now been substantially improved. This reviewer has only a few minor comments.

- Lines 219-222: No data is presented for ID in Fig. 3a.
- Labels in Fig. 3 should be corrected: Fig 3b (L244), Fig. 3d (L274), and Fig. 3e (L275) should be Fig. 3c, Fig. 3c, and Fig. 3d, respectively. No Fig. 3e.
- As for the discussion on the distinction between SBS56 and SBC10c, authors are requested to confirm replication strand bias of SBS56.

Reviewer #3 (Remarks to the Author):

I feel that the authors have addressed all my concerns.
I think that this is an important paper and data that should be published.

Final Decision Letter:

In reply please quote: NG-A56992R Stratton

28th Jul 2021

Dear Mike,

How are you?

I am delighted to say that your manuscript "Increased somatic mutation burdens in normal human cells due to defective DNA polymerases" has been accepted for publication in an upcoming issue of Nature Genetics.

Prior to setting your manuscript, we may make minor changes to enhance the lucidity of the text and with reference to our house style. We therefore ask that you examine the proofs most carefully to ensure that we have not inadvertently altered the sense of your text in any way.

Once your manuscript is typeset and you have completed the appropriate grant of rights, you will receive a link to your electronic proof via email with a request to make any corrections within 48 hours. If, when you receive your proof, you cannot meet this deadline, please inform us at rjsproduction@springernature.com immediately.

Your paper will be published online after we receive your corrections and will appear in print in the next available issue. You can find out your date of online publication by contacting the Nature Press Office (press@nature.com) after sending your e-proof corrections. Now is the time to inform your Public Relations or Press Office about your paper, as they might be interested in promoting its publication. This will allow them time to prepare an accurate and satisfactory press release. Include your manuscript tracking number (NG-A56992R) and the name of the journal, which they will need when they contact our Press Office.

Before your paper is published online, we shall be distributing a press release to news organizations worldwide, which may very well include details of your work. We are happy for your institution or funding agency to prepare its own press release, but it must mention the embargo date and Nature Genetics. Our Press Office may contact you closer to the time of publication, but if you or your Press Office have any enquiries in the meantime, please contact press@nature.com.

Acceptance is conditional on the data in the manuscript not being published elsewhere, or announced in the print or electronic media, until the embargo/publication date. These restrictions are not intended to deter you from presenting your data at academic meetings and conferences, but any enquiries from the media about papers not yet scheduled for publication should be referred to us.

Please note that <i>Nature Genetics</i> is a Transformative Journal (TJ). Authors may publish their research with us through the traditional subscription access route or make their paper immediately

open access through payment of an article-processing charge (APC). Authors will not be required to make a final decision about access to their article until it has been accepted. Find out more about Transformative Journals

<B>Authors may need to take specific actions to achieve compliance with funder and institutional open access mandates. For submissions from January 2021, if your research is supported by a funder that requires immediate open access (e.g. according to Plan S principles) then you should select the gold OA route, and we will direct you to the compliant route where possible. For authors selecting the subscription publication route our standard licensing terms will need to be accepted, including our self-archiving policies. Those standard licensing terms will supersede any other terms that the author or any third party may assert apply to any version of the manuscript.

Please note that Nature Research offers an immediate open access option only for papers that were first submitted after 1 January, 2021.

You will not receive your proofs until the publishing agreement has been received through our system.

If you have any questions about our publishing options, costs, Open Access requirements, or our legal forms, please contact ASJournals@springernature.com

If you have posted a preprint on any preprint server, please ensure that the preprint details are updated with a publication reference, including the DOI and a URL to the published version of the article on the journal website.

To assist our authors in disseminating their research to the broader community, our SharedIt initiative provides you with a unique shareable link that will allow anyone (with or without a subscription) to read the published article. Recipients of the link with a subscription will also be able to download and print the PDF.

As soon as your article is published, you will receive an automated email with your shareable link.

You can now use a single sign-on for all your accounts, view the status of all your manuscript submissions and reviews, access usage statistics for your published articles and download a record of your refereeing activity for the Nature journals.

An online order form for reprints of your paper is available at https://www.nature.com/reprints/author-reprints.html. Please let your coauthors and your institutions' public affairs office know that they are also welcome to order reprints by this method.

If you have not already done so, we invite you to upload the step-by-step protocols used in this manuscript to the Protocols Exchange, part of our on-line web resource, natureprotocols.com. If you

complete the upload by the time you receive your manuscript proofs, we can insert links in your article that lead directly to the protocol details. Your protocol will be made freely available upon publication of your paper. By participating in natureprotocols.com, you are enabling researchers to more readily reproduce or adapt the methodology you use. Natureprotocols.com is fully searchable, providing your protocols and paper with increased utility and visibility. Please submit your protocol to https://protocolexchange.researchsquare.com/. After entering your nature.com username and password you will need to enter your manuscript number (NG-A56992R). Further information can be found at https://www.nature.com/nprot/.

I'll just close by saying how pleased I am that you sent this paper to Nature Genetics - we are excited to be publishing it.

With best wishes,

Safia

Safia Danovi
Senior Editor
Nature Genetics
